# YAP delactylation enhances therapy resistance in renal cell carcinoma

**DOI:** 10.1038/s41419-026-09204-z

**Published:** 2026-08-15

**Authors:** Mingjie Lin, Xiaohan Jin, Minyu Chen, Liangmin Fu, Kun Ye, Yong Huang, Peixian Liang, Lei Tan, Xinwei Zhou, Jinchen Luo, Guannan Shu, Yuhang Chen, Gaosheng Yao, Hansen Lin, Zhu Wang, Qiong Deng, Wei Chen, Junhang Luo, Jiefeng Yang, Hui Liang, Jinhuan Wei

**Affiliations:** 1 Department of Urology, The First Affiliated Hospital, Sun Yat-sen University, Guangzhou, China; 2 State Key Laboratory of Respiratory Disease, Institute of Pulmonary Diseases, Department of Oncology, Guangzhou Chest Hospital, Guangzhou Medical University, Guangzhou, P. R. China; 3 Department of Urology, The Second Xiangya Hospital of Central South University, Changsha, Hunan China; 4 Key Laboratory of Diabetes Immunology (Central South University), Ministry of Education, National Clinical Research Center for Metabolic Disease, Changsha, China; 5 Guangxi Hospital Division of The First Affiliated Hospital, Sun Yat-sen University, Nanning, China; 6 Department of Ultrasound, Guangdong Provincial Hospital of Chinese Medicine, The Second Affiliated Hospital of Guangzhou University of Chinese Medicine, Guangzhou, China; 7 The Fourth Affiliated Hospital of Guangzhou Medical University, Urology Department, Guangzhou, China; 8 Department of Urology, Nanfang Hospital, Southern Medical University, Guangzhou, China; 9 Department of Urology, Guangzhou Women and Children’s Medical Center, Guangzhou Medical University, Guangdong Provincial Clinical Research Center for Child Health, Guangzhou, China; 10 Department of Genitourinary Oncology, Tianjin Medical University Cancer Institute and Hospital, National Clinical Research Center for Cancer, Key Laboratory of Cancer Prevention and Therapy, Tianjin’s Clinical Research Center for Cancer, Tianjin, China; 11 Department of Urology, The People’s Hospital of Longhua, Shenzhen, Guangdong China; 12 Institute of Precision Medicine, First Affiliated Hospital, Sun Yat-sen University, Guangzhou, China; 13 Digestive Diseases Center, Guangdong Provincial Key Laboratory of Digestive Cancer Research, The Seventh Affiliated Hospital, Sun Yat-Sen University, Shenzhen, China

**Keywords:** Cancer therapeutic resistance, Post-translational modifications, Cancer metabolism

## Abstract

Lactylation is a novel lysine acylation modification, and its function and exact mechanisms remain unclear. The responsiveness to combined targeted and immunotherapy continues to be a major challenge in cancer treatment. Here, we discovered that lysine lactylation (Kla) is specifically downregulated in targeted therapy-resistant renal cancer cells and tissues. The lactylation status of YAP at lysine 90 (K90) regulates its nuclear translocation, which is critical for the expression of epithelial-mesenchymal transition (EMT)-related genes and the PD-L1 gene. Furthermore, we identified HDAC1 as the “eraser” of YAP-K90 lactylation. By transfecting YAP-K90T (a lactylation mimic), we enhanced antitumor immune activity. In xenograft models established in huCD34^+^HSC NCG mice, we consistently found that HDAC1 inhibition enhances the response to combination therapy by increasing YAP lactylation and promoting CD8⁺ T cell tumor infiltration. These findings reveal lactylation as a key mechanism in targeted drug response and identify inhibiting YAP delactylation as a promising strategy for renal cell carcinoma treatment.

## Introduction

The incidence of kidney cancer has been steadily increasing over the past few decades [1], with over 85% diagnosed as renal cell carcinoma (RCC) [2]. Surgery is the primary treatment modality. However, approximately 30% of patients have already experienced metastasis at the time of diagnosis [3]. Sunitinib is an oral multitarget receptor tyrosine kinase inhibitor (TKI) and is a first-line targeted therapy for patients with RCC [4]. However, some RCC patients demonstrate resistance to TKI treatment, and even those who respond may experience relapse shortly after starting therapy [5]. Subsequently, the development of targeted drugs combined with immune checkpoint inhibitors (ICIs) changed the management of advanced RCC. However, most RCC patients still do not achieve lasting clinical benefits [6]. Currently, the biological characteristics and potential mechanisms of resistance to combined targeted and immune therapy remain unclear.

Tumors often exhibit metabolic reprogramming that promotes cancer progression. The Warburg effect posits that a high lactate microenvironment generated by glycolysis can facilitate tumorigenesis [7]. However, there is heterogeneity and flexibility in metabolism between different tumors and even within various regions of solid tumors [8]. Currently, the mechanisms underlying cancer development remain unclear, particularly regarding their relationship with the Warburg effect, which is still in its early stages of research. Recently, Zhang and colleagues discovered that, beyond its metabolic roles, lactate induces a novel post-translational modification (PTM) known as lactylation. They found that lactylation of histone lysine residues acts as an epigenetic modification that regulates gene transcription [9]. Evidence indicates that lactylation is involved in various biological processes, such as tumor resistance, immune cell suppression, fibrosis, and vascular injury [9–14]. However, the mechanism by which lactylation regulates resistance to combined TKI targeted and immune therapy remains largely unclear.

Herein, we demonstrated that lactylation regulates the response of RCC to combined targeted and immune therapy. Mechanistically, YAP delactylation promotes resistance to targeted therapy. HDAC1-mediated delactylation at YAP-K90 suppresses YAP phosphorylation and regulates YAP’s subcellular localization and transcriptional activity. On the other hand, YAP enhances resistance to combined targeted and immune therapy at the transcriptional level by upregulating epithelial-mesenchymal transition (EMT) related genes and programmed death-ligand 1 (PD-L1). Therefore, this study reveals how lactylation signaling modulates Hippo-YAP from a molecular perspective, suggesting that targeting YAP delactylation may be an effective strategy to overcome resistance to combined targeted and immune therapy in RCC.

## Results

### Lactylation inhibits TKI targeted treatment resistance in renal cancer

**Fig. 1:**
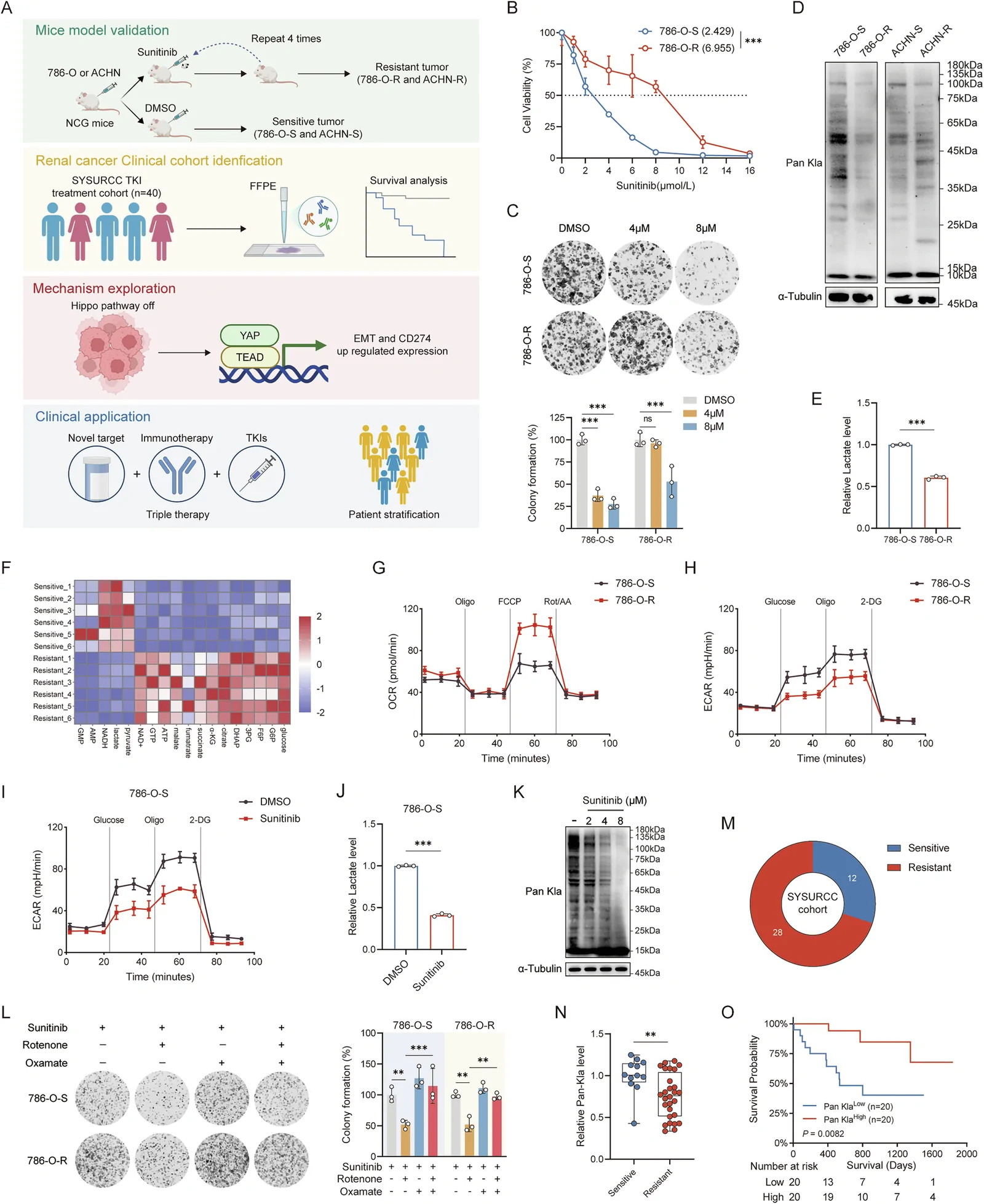
Lactylation inhibits TKI targeted treatment resistance in renal cancer. **A** Schematic overview of the workflow performed in this study. Created with BioRender.com. **B** Relative cell viability of 786-O cells calculated by CCK8. **C** Above: colony formation assay treating 786-O cells (1,000 cells per well in a 6-well plate) with sunitinib for 7 days; Below: Quantification of the data. **D** Immunoblot analysis of pan-lactylation levels in 786-O and ACHN cells. **E** Lactate level measurement in 786-O cells. **F** The heatmap of metabolomic analysis between sunitinib sensitive and resistant cells. OCR (**G**) and ECAR (**H**) measurement in 786-O cells. ECAR (**I**) and lactate level (**J**) were measured in 786-O-S cells treated with 4 μmol/L sunitinib or DMSO. **K** The pan-lactylation level was measured by Immunoblot analysis in 786-O-S cells treated with sunitinib or DMSO. **L** 786-O cells (1000 cells per well in a 6-well plate) were treated with 4 μmol/L sunitinib, 20 mmol/L Oxamate, and 50 nmol/L Rotenone, respectively, for colony formation assays (left) and data quantification (right). **M** Proportion of sensitive and resistant tumors in the SYSURCC cohort. **N** Comparison of Pan-Kla levels between sensitive and resistant tumors in the SYSURCC cohort. **O** Kaplan-Meier analysis of overall survival in the SYSURCC cohort stratified by Pan-Kla levels. The results shown are representative images from three independent experiments (*n* = 3). The statistical data in **B**, **E**, **J**, and **N** are analyzed using unpaired two-tailed Student’s *t*-test. The statistical data in **C** and **L** are analyzed using two-way ANOVA followed by multiple comparisons test. The Log-rank test is used for panels O. Data are presented as mean ± SD. ns, not significant; **p < 0.01, ***p < 0.001.

Increasing evidence suggests that lactylation is involved in tumor progression, metastasis, and resistance [15–17]. To identify key proteins potentially undergoing lactylation modification in RCC and to investigate their role in resistance to TKI targeted therapy, we divided the study into four distinct phases. First, we established two types of sunitinib-resistant RCC cell lines in vivo, designated as 786-O-R and ACHN-R. We also collected a clinical cohort from the First Affiliated Hospital of Sun Yat-sen University, comprising RCC patients treated with sunitinib. These patients were categorized into treatment-sensitive and treatment-resistant groups to validate the impact of lactylation levels on treatment response. Furthermore, we investigated the underlying mechanisms driving therapy resistance and ultimately proposed potential translational strategies (Fig. 1A). Compared with the primary generation, third-generation RCC cells were significantly more resistant to sunitinib in mice (Fig. S1A). After isolation from third-generation xenograft tumors, the resistant RCC cells were cultured in vitro in the absence of sunitinib. The IC50 values measured after 1 and 20 passages under drug-free conditions showed no significant difference (Fig. S1B), indicating that the acquired sunitinib-resistant phenotype was stably maintained in vitro without continuous drug selection pressure. Compared to the corresponding parental cells (786-O-S and ACHN-S), 786-O-R and ACHN-R cells exhibited reduced sensitivity to sunitinib, as indicated by an increased half maximal inhibitory concentration (IC50) (Fig. 1B and S1C) and enhanced colony formation ability (Fig. 1C and S1D) following sunitinib treatment.

Next, to investigate the significance of PTMs in RCC TKI resistance, pan-antibodies for four newly identified lysine acylation modifications were used to assess the PTM profiles of sunitinib-sensitive and resistant RCC cell lines in vitro [9, 18–20]. We observed that, compared to the corresponding sensitive cells, only Kla was significantly reduced in the sunitinib-resistant strains (Fig. 1D), while no significant changes were detected for other lysine acylation modifications, including lysine crotonylation (Kcr), lysine malonylation (Kma), and lysine succinylation (Ksu) (Figs. S1E–S1G). We measured the intracellular lactate levels in RCC sensitive and resistant cells, and the results indicated that the lactate levels were relatively lower in the resistant cells (Fig. 1E and S1H). Metabolomic analysis revealed a metabolic shift from glycolysis to oxidative phosphorylation in resistant cells (Fig. 1F), as evidenced by increased glucose levels and decreased lactate levels (Figs. S1I, S1J). Furthermore, measurements of oxygen consumption rate (OCR) and extracellular acidification rate (ECAR) in both sensitive and resistant cells supported this finding (Fig. 1G, H and S1K, S1L). We next investigated whether sunitinib treatment directly suppresses glycolysis and lactate production in RCC cells. Therefore, we treated the sensitive cells (786-O-S and ACHN-S) with sunitinib. The results showed that after sunitinib treatment, the ECAR (Fig. 1I and S1M), intracellular lactate levels (Fig. 1J and S1N), and pan-Kla modification levels (Fig. 1K and S1O) in the cells all decreased. In addition, treatment with other TKI drugs such as cabozantinib or lenvatinib also reduced the lactylation levels in renal cancer cells (Fig. S1P). Treatment with the oxidative phosphorylation inhibitor rotenone increased lactylation levels, whereas the lactate dehydrogenase A (LDHA) inhibitor oxamate reduced lactylation (Figs. S1Q–S1R). Moreover, oxamate promoted sunitinib resistance, while rotenone had the opposite effect (Fig. 1L and S1S). This suggests that the decrease in lactate may promote RCC targeted therapy resistance through the lactylation of certain specific proteins. We also collected samples from 40 patients treated with targeted therapy, comprising 12 treatment-sensitive and 28 treatment-resistant tumor samples (Fig. 1M). Compared to sensitive tumors, resistant tumors exhibited reduced pan-Kla levels (Fig. 1N), and lower pan-Kla levels were associated with poorer prognosis (Fig. 1O). Taken together, these results suggest that protein delactylation may play a key role in promoting RCC TKI targeted treatment resistance.

### Lactate induces YAP-K90 lactylation

**Fig. 2:**
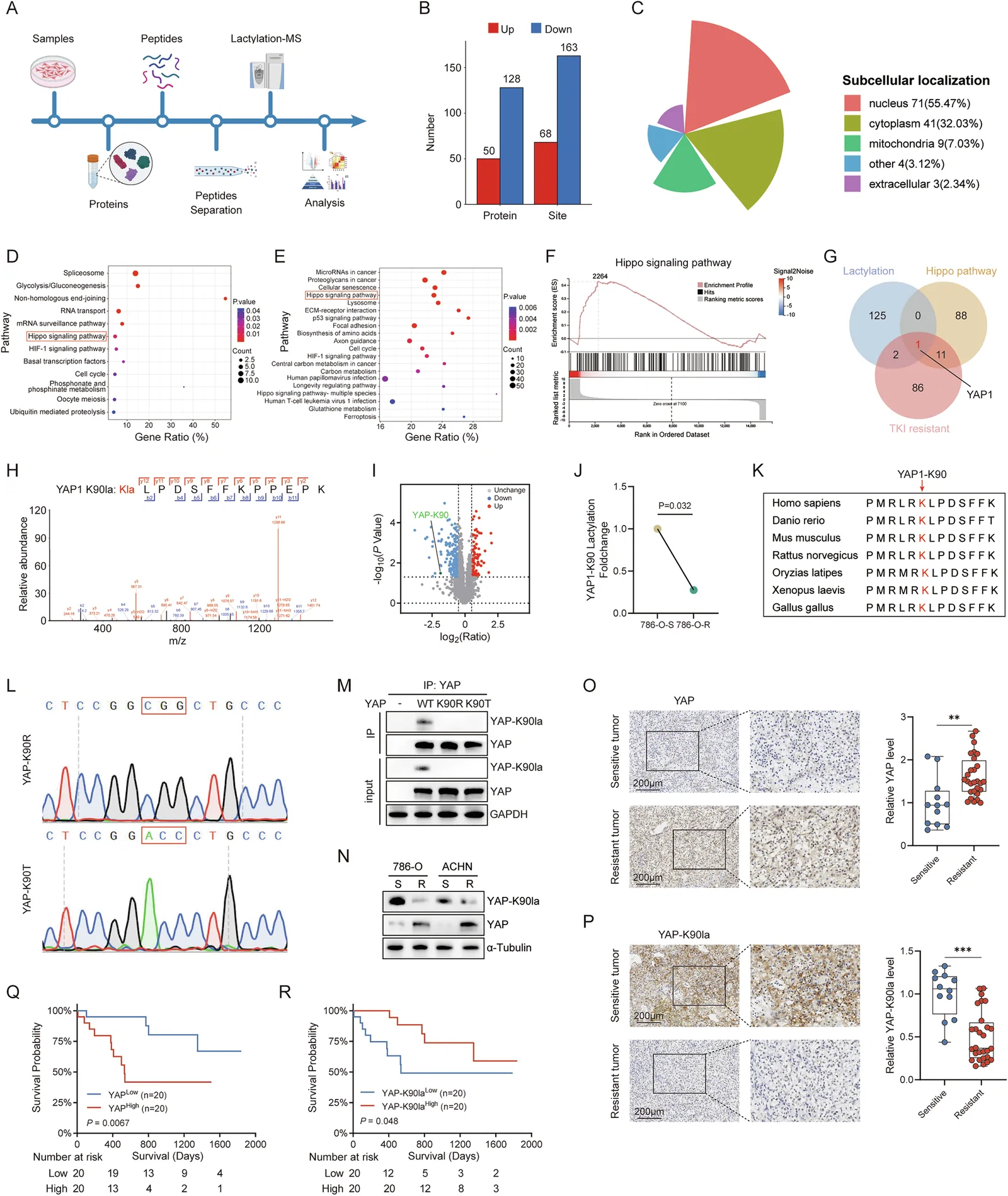
Lactate induces YAP-K90 lactylation. **A** The lysine lactylation proteomics scheme for 786-O-R and 786-O-S cells. Created with BioRender.com. **B** DEKPs and DEKSs identified by lactylation proteomics of 786-O-R and 786-O-S cells. **C** Subcellular distribution of DEKPs in 786-O-R and 786-O-S cells. **D** KEGG enrichment analysis of down-regulated DEKPs. **E** KEGG enrichment analysis of transcriptomics DEGs. **F** GSEA analysis of transcriptomics DEGs. **G** Venn diagram showing the overlap among downregulated Kla proteins, Hippo pathway proteins, and TKI resistance proteins. **H** Identification and quantification of YAP K90 lactylation. LC-MS/MS analysis of modified (Kla)LPDSFFKPPEPK is shown. **I** The volcano plot displays the differentially expressed Kla sites (DEKSs). **J** YAP-K90 lactylation relative ratio. **K** Sequence alignment of the predicted lactylation site K90 in YAP from the specified species. **L** Sanger sequencing chromatograms of YAP target sites from 786-O YAP-K90R and 786-O YAP-K90T cell genomic DNA. **M** Immunoblot analysis of YAP-K90la in 786-O YAP-KO cells re-expressing YAP-WT, YAP-K90R, or YAP-K90T, demonstrating the specificity of the YAP-K90la monoclonal antibody. **N** Immunoblot analysis of YAP-K90 lactylation levels in 786-O and ACHN cells. **O** Representative images (left) and quantification (right) of YAP levels in sensitive versus resistant tumors from the SYSURCC cohort. **P** Representative images (left) and quantification (right) of YAP-K90la levels in sensitive versus resistant tumors from the SYSURCC cohort. **Q** Kaplan-Meier analysis of overall survival in the SYSURCC cohort stratified by YAP levels. **R** Kaplan-Meier analysis of overall survival in the SYSURCC cohort stratified by YAP-K90la levels. The results shown are representative images from three independent experiments (*n* = 3). The statistical data in **J**, **O**, and **P** are analyzed using unpaired two-tailed Student’s t-test. The Log-rank test is used for panels Q and R. Data are presented as mean ± SD. ***p < 0.001.

The functional role of Kla in cancer is currently unclear. To map the lactylation modification profile in sunitinib-resistant cells, we used Kla pan-antibody-conjugated beads to enrich modified peptides prior to high-resolution liquid chromatography-tandem mass spectrometry (LC-MS/MS) analysis (Fig. 2A). Principal component analysis (PCA) showed good reproducibility among the samples (Fig. S2A). Overall, compared to sensitive cells, we identified 231 differentially expressed Kla sites (DEKSs) among 178 differentially expressed Kla proteins (DEKPs) in sunitinib-resistant cells (Fig. 2B). Notably, the overall level of lactylation in resistant cells was lower than that in sensitive cells (Figs. S2B, S2C). Subcellular localization analysis revealed that over half (55.47%) of the DEKPs were localized in the nucleus (Fig. 2C). Next, by conducting KEGG enrichment analysis on the downregulated proteins with lactylation modifications, we found that they were enriched in the Hippo signaling pathway (Fig. 2D). More importantly, we also performed transcriptome sequencing on both sensitive cells and sunitinib-resistant cells. KEGG enrichment analysis indicated enrichment in the Hippo signaling pathway (Fig. 2E). GSEA analysis results showed that genes associated with Hippo signaling were significantly enriched in resistant cells (Fig. 2F). Subsequently, took the intersection of the downregulated lactylated proteins in the resistant cells with key proteins in the Hippo signaling pathway and those known to be critical for TKI resistance, which identified YAP1 (Fig. 2G). Secondly, LC-MS/MS analysis revealed lactylation at the K90 residue of YAP (Fig. 2H), and the lactylation level of YAP K90 was decreased in resistant cells (Figs. 2I, J). YAP-K90 is an evolutionarily conserved lysine residue (Fig. 2K). Immunoprecipitation data also indicated that YAP undergoes lactylation(Fig. S2D). To validate lactylation at the YAP-K90 site, we generated a rabbit monoclonal antibody specifically recognizing YAP lactylated at lysine 90 (Fig. S2E). We also constructed mutants in which the YAP-K90 site was replaced with arginine (YAP-K90R) or threonine (YAP-K90T) (Fig. 2L). Next, we first constructed YAP-KO RCC cells using CRISPR/Cas9 technology (Fig.S2F). Using this specific anti-YAP-K90la antibody, we readily detected endogenous YAP lactylation at K90 (Fig. 2M). Compared to sunitinib-sensitive cells, resistant cells exhibited reduced YAP-K90 lactylation levels (Fig. 2N). Notably, overexpression of YAP increased the IC50 for sunitinib in sensitive cells and promoted their proliferation (Figs. S2G–S2I). Conversely, knockdown of YAP produced the opposite effects (Figs. S2J–S2L). This suggests that increased YAP expression promotes RCC sunitinib resistance, although the role of lactylation in this process remains unclear. Therefore, YAP was chosen as the focus of our study.

YAP is a key component of the Hippo pathway. When the Hippo pathway is activated, a series of cascading kinase reactions activate LATS1/2. Activated LATS1/2 then phosphorylate and inhibit YAP, preventing it from translocating to the nucleus and interacting with transcriptional enhanced associate domain (TEAD) [21]. Next, we treated the sensitive cells with rotenone and oxamate. We found that rotenone significantly reduced the non-phosphorylated YAP in a dose-dependent manner, while increasing the levels of phosphorylated YAP and YAP K90 lactylation (Fig. S2M). In contrast, oxamate treatment had the opposite effect (Fig. S2N). These findings suggest that YAP-K90 lactylation is negatively associated with YAP activation. To investigate the clinical relevance of YAP in targeted therapy resistance in RCC, we analyzed patient samples. We found that resistant tumors exhibited significantly increased YAP expression but decreased lactylation at the K90 site (Fig. 2O, P). Clinically, high YAP expression and low YAP-K90la levels were both associated with poorer patient prognosis (Fig. 2Q, R). These findings identify YAP-K90 lactylation as a clinically relevant modification associated with YAP activation and targeted therapy resistance in RCC.

### YAP K90 is delactylated by HDAC1

**Fig. 3:**
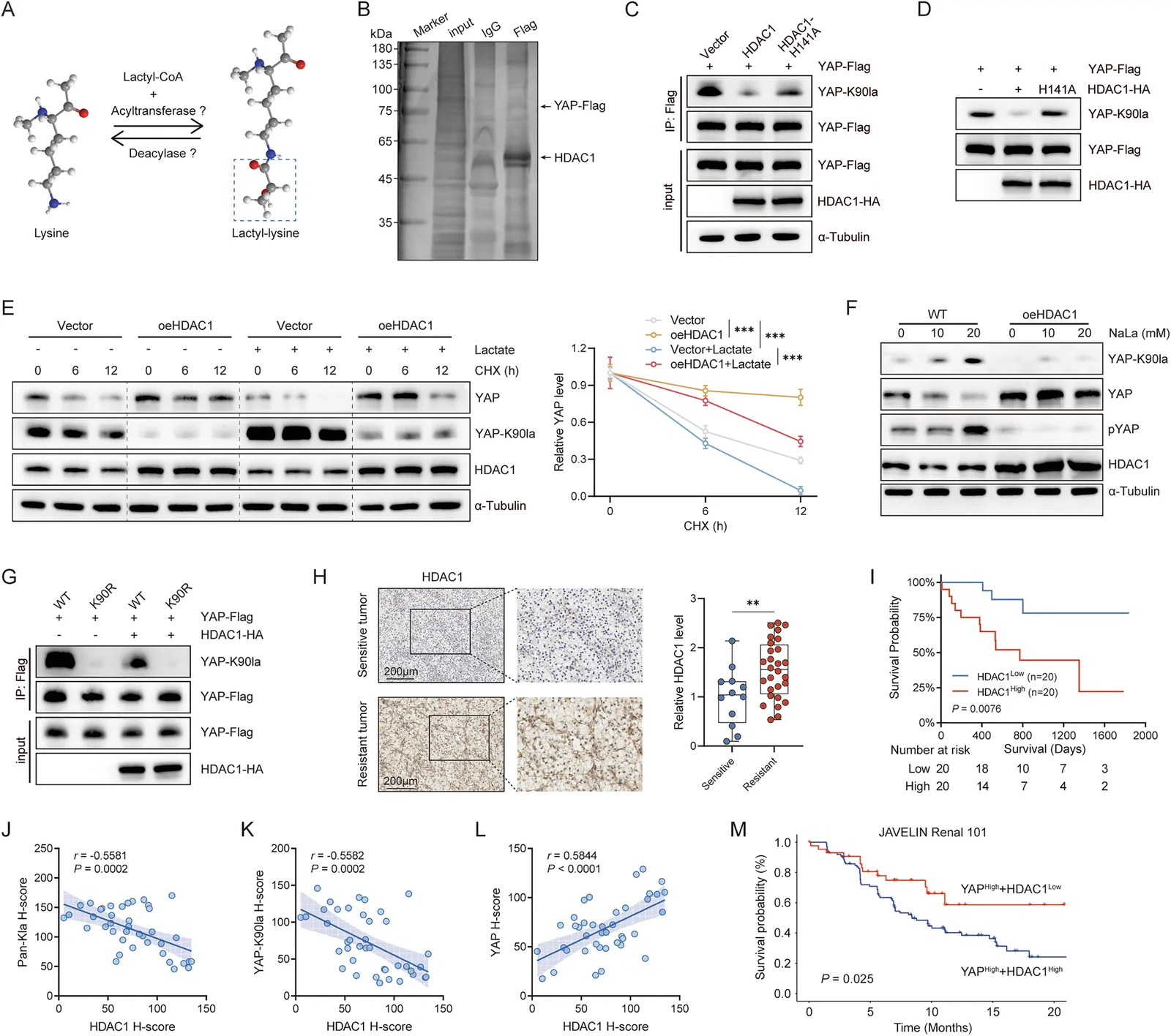
YAP K90 is delactylated by HDAC1. **A** Hypothesized mechanism of lactyl-lysine. **B** Silver staining of the gel revealed a specific protein band (indicated by the arrow) present in the Flag-YAP pulldown sample in 786-O cells. **C** Effect of the HDAC1-H141A mutant on YAP-K90la levels in 786-O cells. **D** YAP-K90la levels were determined in vitro in the presence of wild-type HDAC1 or HDAC1 catalytic inactivation mutant (HDAC1- H141A). **E** Immunoblot analysis (left) and quantification (right) of protein stability in 786-O cells overexpressing HDAC1 with or without lactate treatment following CHX exposure. **F** Immunoblot analysis of 786-O cells, either wild-type or overexpressing HDAC1, treated with 10 mmol/L sodium lactate (NaLa) for 24 h. **G** Immunoblot analysis of YAP-K90la levels in YAP-WT or K90R co-transfected with HDAC1 cells. **H** Representative images (left) and quantification (right) of HDAC1 levels in sensitive versus resistant tumors from the SYSURCC cohort. **I** Kaplan-Meier analysis of overall survival in the SYSURCC cohort stratified by HDAC1 levels. **J** Correlation analysis of HDAC1 and Pan-Kla levels in the SYSURCC cohort. **K** Correlation analysis of HDAC1 and YAP-K90la levels in the SYSURCC cohort. **L** Correlation analysis of HDAC1 and YAP levels in the SYSURCC cohort. **M** Kaplan-Meier analysis of Progression-free survival in patients from the JAVELIN Renal 101 Phase 3 Trial, stratified by tumor YAP and HDAC1 level. The results shown are representative images from three independent experiments (*n* = 3). The statistical data in E are analyzed using one-way ANOVA followed by multiple comparisons test. The statistical data in H are analyzed using unpaired two-tailed Student’s *t*-test. The Log-rank test is used for panels I and M. Spearman correlation analysis is used for panels **J**, **K**, and **L**. Data are presented as mean ± SD. ***p < 0.001.

Identifying the enzymes responsible for acetylation and deacetylation related to Kla modification is important, but relevant studies remain unclear. There is evidence suggesting that some newly discovered PTMs are regulated by enzymes previously classified as histone acetyltransferases (HATs) and histone deacetylases (HDACs) [22]. To investigate this, we performed immunoprecipitation assays to detect the interaction between YAP and HATs or HDACs (Fig. 3A). Among the proteins in the HDAC superfamily, including HDAC1-11 and sirtuins (SIRT1-7), we found that HDAC1, HDAC11 and SIRT3 can regulate the lactylation state of YAP-K90 (Fig. S3A). Further experiments showed that only HDAC1, and not HDAC11 or SIRT3, could directly interact with YAP (Fig. S3B). In addition, silver staining and co-immunoprecipitation (CO-IP) experiments confirmed the binding of HDAC1 to YAP in RCC cells (Fig. 3B and S3C). The catalytically inactive HDAC1 mutant (HDAC1-H141A) [23] reduced the ability to regulate the YAP Kla levels (Fig. 3C). Consistently, when wild-type HDAC1 was added, Kla levels decreased, whereas the addition of the mutant HDAC1 had no such effect, indicating that HDAC1 acts as a de-lactylase for YAP-Kla in vitro (Fig. 3D). Furthermore, we found that HDAC1-mediated modulation of YAP-K90 lactylation was associated with changes in YAP protein stability (Fig. 3E). Overexpression of HDAC1 significantly reduced sodium lactate-induced K90 lactylation of endogenous YAP (Fig. 3F). Notably, alterations in YAP-K90 lactylation closely paralleled changes in YAP phosphorylation, supporting a functional link between K90 lactylation and Hippo pathway-mediated YAP inhibition. Importantly, overexpression of HDAC1 increased the resistance of renal cancer cells to sunitinib (Fig. S3D), while sunitinib treatment elevated the protein levels of HDAC1 (Fig. S3E). These results suggest that HDAC1 is a de-lactylase capable of catalyzing the removal of YAP-K90la. This is consistent with the previous study by Zhao and his colleagues [24].

To further validate our conclusions, we generated two YAP mutants: the K90R mutant [25], which mimics delactylation, and the K90T mutant [26], which mimics lactylation. Consistent with the site specificity of the YAP-K90la antibody, neither mutant was recognized by the YAP-K90la antibody (Fig. S3F). Importantly, co-immunoprecipitation analyses demonstrated that HDAC1 reduced the lactylation level of YAP-WT, supporting its role as a YAP K90 de-lactylase (Fig. 3G). Furthermore, both YAP-K90R and YAP-K90T retained interaction with HDAC1, indicating that mutation of the K90 residue does not disrupt the YAP–HDAC1 association (Fig. S3G). Together, these findings suggest that the loss of YAP-K90la signal observed in the K90R and K90T mutants is attributable to the lack of antibody recognition rather than impaired HDAC1 binding. Moreover, re-expression of YAP-K90R in YAP-KO cells restored resistance to sunitinib, whereas re-expression of YAP-K90T did not (Fig. S3H). We next assessed the clinical significance of HDAC1. Its expression was significantly upregulated in resistant tumors compared to sensitive ones (Fig. 3H). Consistent with a potential role in driving resistance, high HDAC1 expression was associated with an unfavorable patient prognosis (Fig. 3I). We first observed a significant negative correlation between HDAC1 expression and the levels of both pan-Kla and YAP-K90la in clinical samples (Fig. 3J, K), alongside a positive correlation with total YAP protein (Fig. 3L). To further investigate the clinical interplay between YAP and HDAC1, we analyzed data from the JAVELIN Renal 101 trial [27]. This analysis revealed that among patients with high YAP expression, those with concurrently high HDAC1 levels had significantly shorter progression-free survival (PFS) (Fig. 3M). Notably, in patients with low YAP expression, HDAC1 levels were not associated with PFS (Fig. S3I). Collectively, these results underscore the clinical importance of HDAC1 and YAP-K90 lactylation in RCC progression, particularly in the development of targeted therapy resistance. The inverse relationship between elevated HDAC1 and diminished YAP-K90la in resistant tumors suggests their functional interplay in driving the resistant phenotype.

### Delactylation of YAP disrupts its interaction with LATS1, leading to reduced phosphorylation, enhanced nuclear localization, and ultimately, transcriptional activation of TEAD

**Fig. 4:**
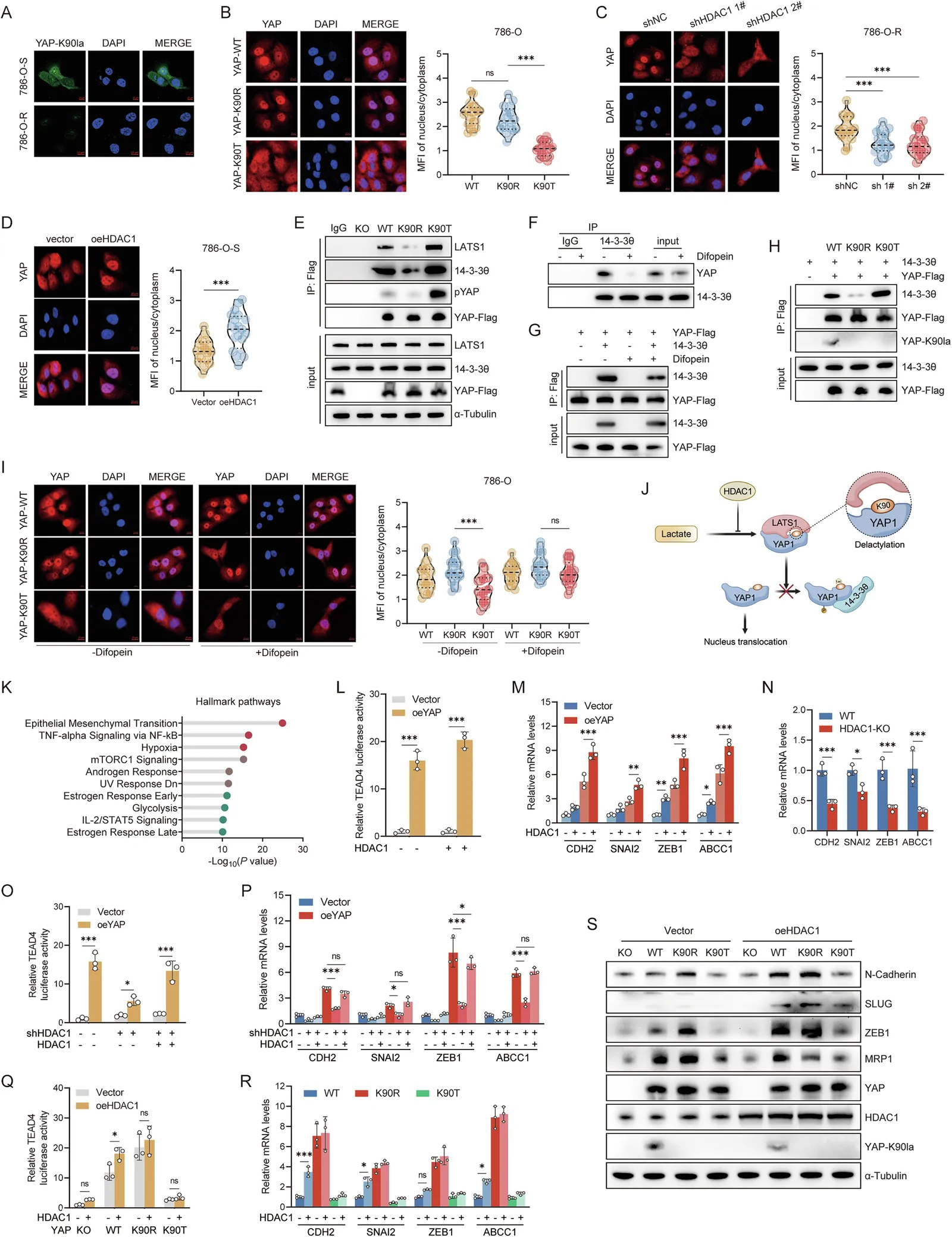
Delactylation of YAP disrupts its interaction with LATS1, leading to reduced phosphorylation, enhanced nuclear localization, and ultimately, transcriptional activation of TEAD. **A** Immunofluorescence analysis (left) and quantitative data (right) of YAP-Kla and in 786-O-S and 786-O-R cells. **B** Immunofluorescence analysis (left) and quantitative data (right) of YAP-WT, YAP-K90R and YAP-K90T cell localization in 786-O cells. **C** Immunofluorescence analysis (left) and data quantification (right) of changes in YAP cellular localization in HDAC1-knockdown 786-O-R cells. **D** Immunofluorescence analysis (left) and data quantification (right) of changes in YAP cellular localization in HDAC1-overexpressing 786-O-S cells. **E** Immunoblot analysis of the interaction of YAP with LATS1 and 14-3-3θ in YAP-KO 786-O cells reconstituted with YAP-WT, YAP-K90R, or YAP-K90T. **F** Immunoblot analysis of YAP interaction with 14-3-3θ in difopein-treated 786-O cells. **G** Immunoblot analysis of 14-3-3θ and YAP interactions with and without difopein treatment. **H** Immunoblot analysis of the interaction between 14-3-3θ and YAP mutations. **I** Immunofluorescence analysis (left) and data quantification (right) of difopein-treated 786-O cells with anti-YAP antibody and detection of changes in cellular localization of YAP-WT, K90R and K90T. **J** Pattern diagram of HDAC1-mediated K90 delactylation blocking YAP interaction with 14-3-3θ to promote YAP nuclear localization. Created with BioRender.com. **K** Hallmark pathways enrichment analysis of transcriptomics DEGs. **L** Relative TEAD4-dependent luciferase reporter activity in 786-O cells co-transfected with YAP and HDAC1. **M** mRNA expression levels of YAP target genes in 786-O cells after overexpressing HDAC1. **N** mRNA expression levels of YAP target genes in WT and HDAC1 KO 786-O cells. **O** Re-expression of HDAC1 rescues the suppression of TEAD4 luciferase activity caused by HDAC1 knockdown in 786-O cells. **P** Re-expression of HDAC1 rescues the suppression of YAP target gene mRNA expression caused by HDAC1 knockdown in 786-O cells. **Q** Relative luciferase activity of TEAD4 in YAP-KO 786-O cells reconstituted with YAP-WT, YAP-K90R, or YAP-K90T and co-transfected with an HDAC1 overexpression plasmid. **R** mRNA expression levels of YAP target genes in YAP-KO 786-O cells reconstituted with YAP-WT, YAP-K90R, or YAP-K90T and co-transfected with an HDAC1 overexpression plasmid. **S** Immunoblot analysis of the indicated proteins in YAP-KO 786-O cells reconstituted with YAP-WT, YAP-K90R, or YAP-K90T and co-transfected with an HDAC1 overexpression plasmid. The results shown are representative images from three independent experiments (*n* = 3). Nuclear localization was quantified using MFI, and all images were acquired as single-plane images. The statistical data in **B**, **C**, and **I** are analyzed using one-way ANOVA followed by multiple comparisons test. The statistical data in **D** are analyzed using unpaired two-tailed Student’s *t*-test. The statistical data in **L**–**R** are analyzed using two-way ANOVA followed by multiple comparisons test. ns, not significant; *p < 0.05, **p < 0.01, ***p < 0.001.

YAP is a transcriptional co-activator of the Hippo signaling pathway. YAP is localized in the nucleus, where they bind to TEAD transcription factors and promote the expression of target genes [21, 28]. Given that the transcriptional activity of YAP is closely associated with its nuclear localization, we investigated whether K90 lactylation affects the subcellular distribution of YAP. Immunofluorescence analysis showed that, compared to sensitive cells, most YAP protein in resistant cells was localized in the nucleus (Fig. S4A, S4B). Nuclear localization of K90-lactylated YAP was significantly reduced (Fig. 4A). YAP-K90R was predominantly located in the nucleus, whereas YAP-K90T had significantly reduced nuclear localization (Fig. 4B and S4C–S4D). In summary, these findings suggest that the lactylation state at K90 may regulate YAP’s subcellular localization. Next, we investigated whether HDAC1 influences YAP localization. We found that HDAC1 knockdown facilitated YAP’s translocation from the nucleus to the cytoplasm (Fig. 4C and S4E). Conversely, HDAC1 overexpression promoted the translocation of YAP from the cytoplasm to the nucleus (Fig. 4D and S4F). Consistently, in HDAC1 knockout cells, YAP was primarily located in the cytoplasm compared to wild-type cells (Fig. S4G). These results indicate that HDAC1 regulates the K90 lactylation state to mediate YAP nuclear localization.

HDAC1, as a histone deacetylase, seems unlikely to directly regulate YAP’s nuclear import. Previously, it was mentioned that phosphorylated YAP binds to 14-3-3θ and is retained in the cytoplasm [21]. Our data demonstrate that YAP interacts with 14-3-3θ and LATS1 (Fig. S4H). We then investigated whether HDAC1 affects the nuclear localization of YAP. Our results showed that HDAC1 overexpression reduced the interaction of YAP with 14-3-3θ and LATS1 induced by sodium lactate (Figs. S4I, S4J). Conversely, HDAC1 knockdown increased YAP’s interaction with 14-3-3θ (Figs. S4K, S4L). Consistently, the K90R mutant, but not K90T, significantly reduced the ability to interact with 14-3-3θ and LATS1 (Fig. 4E). Subsequently, we treated cells with difopein, a specific inhibitor of 14-3-3, to block its interaction with target proteins [29]. Difopein treatment reduced its interaction with 14-3-3θ (Figs. 4F, G). Consistently, the K90R mutant lost the ability to interact with 14-3-3θ, while the K90T mutant was associated with enhanced interaction (Fig. 4H), potentially as a consequence of lactylation-dependent modulation of YAP phosphorylation. Furthermore, difopein treatment induced YAP nuclear localization (Fig. 4I and S4M). Collectively, these data indicate that HDAC1-mediated K90 delactylation may disrupt the interaction between YAP, LATS1, and 14-3-3θ, promoting YAP translocation to the nucleus and increasing YAP-mediated transcriptional activity (Fig. 4J).

TEAD proteins are a family of transcription factors, including TEAD1-4, that regulate cell proliferation and stem cell function. They play a crucial role in tumorigenesis [30]. Our data show that overexpression of YAP increased the reporter gene activity of TEAD2 and TEAD4 (Fig. S5A). In addition, we performed Hallmark pathway enrichment analysis on the differentially expressed genes between the resistant and sensitive cells, and the most significantly enriched pathway was EMT (Fig. 4K). Previous studies have shown that EMT is closely associated with TKI resistance [31, 32]. Tumors can also induce resistance by lowering the intracellular concentration of TKIs, such as by enhancing drug efflux [33]. Therefore, we hypothesize that the transcription of EMT-related genes and ABC transporter genes is activated by YAP in drug-resistant cells. PCR results showed that overexpression of YAP significantly promoted the expression of EMT-related genes as well as ABC transporter genes in RCC cells (Figs. S5B, S5C). On the other hand, knockdown of TEAD4 significantly reduced the expression of YAP-mediated EMT-related genes and ABC transporter genes, while knockdown of TEAD2 had negligible effect (Figs. S5D–S5G). Immunoblotting results showed that knockout of TEAD4 significantly reduced the expression of YAP-mediated EMT-related proteins and ABC transporter proteins (Fig. S5H). Expression of HDAC1 significantly increased YAP-mediated TEAD4 reporter gene activity (Fig. 4L) and promoted the expression of YAP-TEAD target genes CDH2, SNAI2, ZEB1, and ABCC1 (Fig. 4M). On the other hand, the loss of HDAC1 suppressed the expression of YAP target genes (Fig. 4N and S5I), and reintroducing HDAC1 was able to rescue YAP target gene expression (Figs. 4O, P). In summary, these results indicate that HDAC1 is a positive regulator of YAP-TEAD transcriptional activity. Next, we examined the role of K90 lactylation in HDAC1-mediated YAP transcriptional activation. We found that the K90R mutant increased TEAD4-dependent transcriptional activity, while the K90T mutant decreased TEAD4 reporter activation (Fig. S5J). Additionally, HDAC1 was unable to activate the K90T mutant form of YAP (Fig. 4Q). Our data indicate that the transcriptional activity of YAP target genes in YAP-K90R mutant cells is stronger than in the wild type, and HDAC1 influences target gene expression in the wild type but has minimal impact in YAP-K90R cells (Fig. 4R, S). Additionally, analysis of data from the JAVELIN Renal 101 trial established that a high EMT signature was associated with an unfavorable prognosis (Fig. S5K). In conclusion, we conclude that HDAC1-mediated delactylation at the K90 site promotes the transcriptional activity of YAP.

Previous studies have shown that the LATS1-binding domain of YAP is located within amino acids 150–300, whereas K90 resides outside this region [34]. To determine whether YAP-K90 lactylation directly regulates the interaction between YAP and LATS1, we generated a YAP fragment encompassing the reported LATS1-binding region (amino acids 150–300). Consistent with our previous observations, HDAC1 overexpression reduced the interaction between full-length YAP and LATS1, whereas HDAC1 knockdown or sodium lactate treatment enhanced this interaction (Fig. S5L). However, none of these manipulations affected the interaction between LATS1 and the YAP(150–300 aa) fragment (Fig. S5M), suggesting that K90 lactylation does not directly alter the intrinsic LATS1-binding domain. Instead, YAP-K90 lactylation may regulate YAP–LATS1 association through conformational changes or additional regulatory factors. Because K90 is located within the TEAD-binding region of YAP, we next investigated whether K90 lactylation affects the assembly of the nuclear YAP transcriptional complex [35, 36]. Co-immunoprecipitation assays were performed in cells co-expressing NLS-actin together with NLS-YAP-WT, NLS-YAP-K90R, or NLS-YAP-K90T. Compared with YAP-WT, the non-lactylatable mutant YAP-K90R exhibited enhanced interactions with both nuclear actin and TEAD4, whereas the lactylation-mimetic mutant YAP-K90T displayed markedly reduced binding (Fig. S5N). These findings indicate that K90 lactylation suppresses the assembly of the YAP–actin–TEAD4 transcriptional complex. To determine the functional consequence of this regulation, TEAD4 luciferase reporter assays were performed. Consistent with the co-immunoprecipitation results, YAP-K90R significantly enhanced TEAD4-dependent transcriptional activity, whereas YAP-K90T markedly reduced reporter activity (Fig. S5O). Furthermore, HDAC1 overexpression further increased TEAD4 transcriptional activity in YAP-WT-expressing cells but failed to rescue the transcriptional deficiency induced by YAP-K90T. Collectively, these findings demonstrate that HDAC1-mediated YAP-K90 delactylation promotes YAP transcriptional activity by facilitating the assembly of the YAP–actin–TEAD4 transcriptional complex. Taken together, our results indicate that YAP-K90 delactylation not only promotes YAP nuclear localization through attenuation of the LATS1–14-3-3 signaling axis but also facilitates the assembly of the YAP–actin–TEAD4 transcriptional complex in the nucleus, thereby maximizing YAP-dependent transcriptional activity.

### YAP-K90 delactylation promotes PD-L1-mediated immunosuppression

**Fig. 5:**
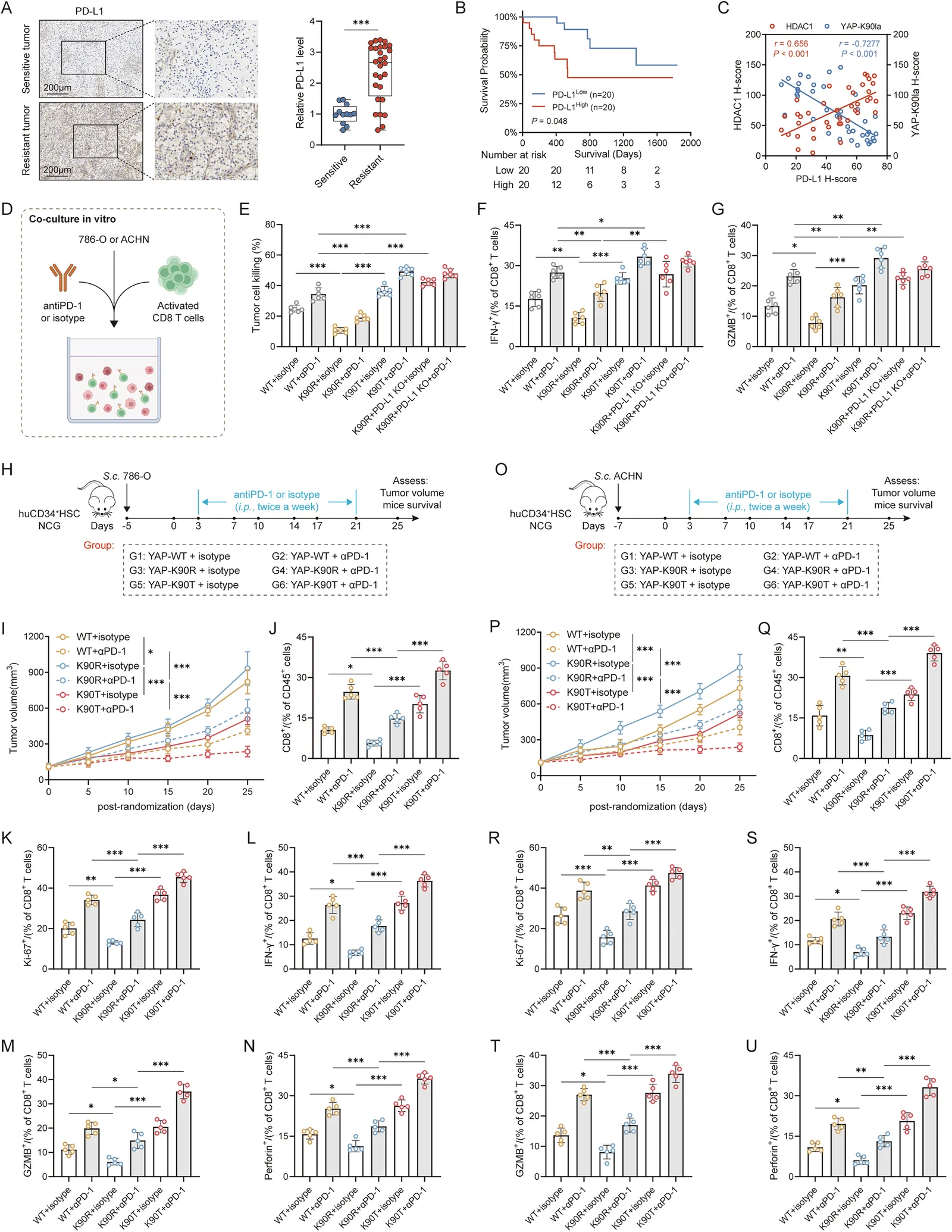
YAP-K90 delactylation promotes PD-L1-mediated immunosuppression. **A** Representative images (left) and quantification (right) of PD-L1 levels in sensitive versus resistant tumors from the SYSURCC cohort. **B** Kaplan-Meier analysis of overall survival in the SYSURCC cohort stratified by PD-L1 levels. **C** Correlation analysis of PD-L1 levels with HDAC1 and YAP-K90la in the SYSURCC cohort. **D** Schematic diagram of the in vitro co-culture system. Created with BioRender.com. **E** Flow cytometric analysis of tumor killing rate after co-culture of 786-O cells with activated CD8⁺ T cells. Flow cytometric analysis of the proportion of IFN-γ⁺ CD8⁺ T cells (**F**) and GZMB⁺ CD8⁺ T cells (**G**) after co-culture with activated 786-O cells. **H** Schematic diagram of the experimental design in huCD34⁺ HSC-NCG humanized mice bearing YAP-KO 786-O tumors reconstituted with YAP-WT, YAP-K90R, or YAP-K90T and treated with anti-PD-1 antibody at the indicated time points. **I** Measurement of subcutaneous tumor volume in huCD34^+^HSC NCG mice bearing 786-O cell-derived tumors. (*n* = 5). **J** Flow cytometric analysis of the percentage of CD8⁺ cells among CD45⁺ cells in subcutaneous tumors derived from the 786-O cell line in huCD34^+^HSC NCG mice. Flow cytometric analysis of the proportions of Ki-67⁺ (**K**), IFN-γ⁺ (**L**), GZMB⁺ (**M**), and perforin⁺ (**N**) cells within the CD8⁺ T cell population in 786-O-derived subcutaneous tumors from huCD34^+^HSC NCG mice. **O** Schematic diagram of the experimental design in huCD34⁺ HSC-NCG humanized mice bearing YAP-KO ACHN tumors reconstituted with YAP-WT, YAP-K90R, or YAP-K90T and treated with anti-PD-1 antibody at the indicated time points. **P** Measurement of subcutaneous tumor volume n subcutaneous tumors derived from the ACHN cell line in huCD34^+^HSC NCG mice. (*n* = 5). **Q** Flow cytometric analysis of the percentage of CD8⁺ cells among CD45⁺ cells in subcutaneous tumors derived from the ACHN cell line in huCD34^+^HSC NCG mice. Flow cytometric analysis of the proportions of Ki-67⁺ (**R**), IFN-γ⁺ (**S**), GZMB⁺ (**T**), and perforin⁺ (**U**) cells within the CD8⁺ T cell population in ACHN-derived subcutaneous tumors from huCD34^+^HSC NCG mice. The statistical data in A are analyzed using unpaired two-tailed Student’s *t*-test. The Log-rank test is used for panels B. Spearman correlation analysis is used for panels **C**. The statistical data in **E**–**G**, **J**–**N** and **Q**–**U** are analyzed using one-way ANOVA followed by multiple comparisons test. The statistical data in I and P are analyzed using two-way ANOVA followed by multiple comparisons test. Data are presented as mean ± SD. *p < 0.05, **p < 0.01, ***p < 0.001.

Numerous studies have revealed that YAP/TEAD promotes PD-L1 transcription by binding to the enhancer region of PD-L1 [37–40]. PD-L1 specifically inhibits the proliferation of CD8^+^ T cells and the secretion of granzyme B (GZMB), leading to reduced CD8^+^ T cell-mediated apoptosis. This enhances immune evasion by tumor cells and drives resistance to EGFR-TKIs [41]. Interestingly, the expression of PD-L1 in RCC sunitinib-resistant cells was significantly higher than in sensitive cells (Fig. S6A). Furthermore, we found that YAP positively regulates PD-L1 expression (Figs. S6B, S6C). Similarly, HDAC1 also acts as a positive regulator of PD-L1 (Figs. S6D, S6E). Consistent with this, the mRNA level of CD274 was regulated by both HDAC1 and YAP (Fig. S6F). The Hippo pathway core kinase LATS1/2 phosphorylates YAP/TAZ, leading to their cytoplasmic retention and functional inactivation [21]. We discovered that HDAC1-mediated YAP-K90 delactylation, which promotes PD-L1 expression, could be further enhanced by knocking down LATS1 (Figs. S6G, S6H). Analysis of clinical samples revealed that PD-L1 expression was significantly elevated in resistant tumor tissues compared to sensitive ones (Fig. 5A). A high level of PD-L1 was correlated with a poorer prognosis (Fig. 5B). Furthermore, in these samples, PD-L1 levels showed a negative correlation with YAP-K90la but a positive correlation with HDAC1 expression (Fig. 5C). In summary, our data support a model where HDAC1-mediated YAP-K90 delactylation interacts with the Hippo pathway to control YAP activity and subsequently modulate PD-L1 expression.

To investigate the role of the YAP-K90la-PD-L1 axis in tumor immune regulation, we assessed its impact on CD8⁺ T cell function within the tumor microenvironment using both in vitro co-culture and in vivo humanized mouse models. In vitro, RCC cells were co-cultured with activated CD8⁺ T cells in the presence of either isotype control IgG or anti-PD-1 antibody (Fig. 5D). Flow cytometry analysis revealed that in the YAP-K90R group (mimicking delactylation), the proportion of tumor cell killing, along with the production of Interferon-gamma (IFN-γ) and Granzyme B (GZMB), were significantly reduced (Fig. 5E–G and S6I–S6K). Notably, knockout of PD-L1 rescued this suppression. Conversely, in the YAP-K90T group, these T cell parameters were increased to some extent. This indicates that enhanced YAP-K90 lactylation can potentiate the anti-tumor activity of CD8⁺ T cells. To validate this finding in a more clinically relevant, immunocompetent setting, we subcutaneously transplanted the human RCC cell lines 786-O and ACHN into huCD34^+^HSC NCG mice. The in vivo results were strikingly consistent with the in vitro findings. Compared to the YAP-WT group, the YAP-K90R group exhibited increased tumor volume, shortened overall survival, and flow cytometry analysis revealed a significant reduction in the proportion of tumor-infiltrating CD8⁺ T cells, their Ki-67 levels, and the production of IFN-γ, GZMB, and perforin, indicating suppressed cytotoxic T cell function. Conversely, the YAP-K90T group showed the opposite effects (Fig. 5H–U and S6L, S6M). Collectively, our integrated in vitro and in vivo data demonstrate that preventing YAP-K90 delactylation effectively remodels the tumor immune microenvironment, promoting the recruitment and functional activation of CD8⁺ T cells, thereby augmenting the anti-tumor immune response.

### YAP-K90R remodels TIME and impairs the response to combined targeted therapy and immunotherapy

**Fig. 6:**
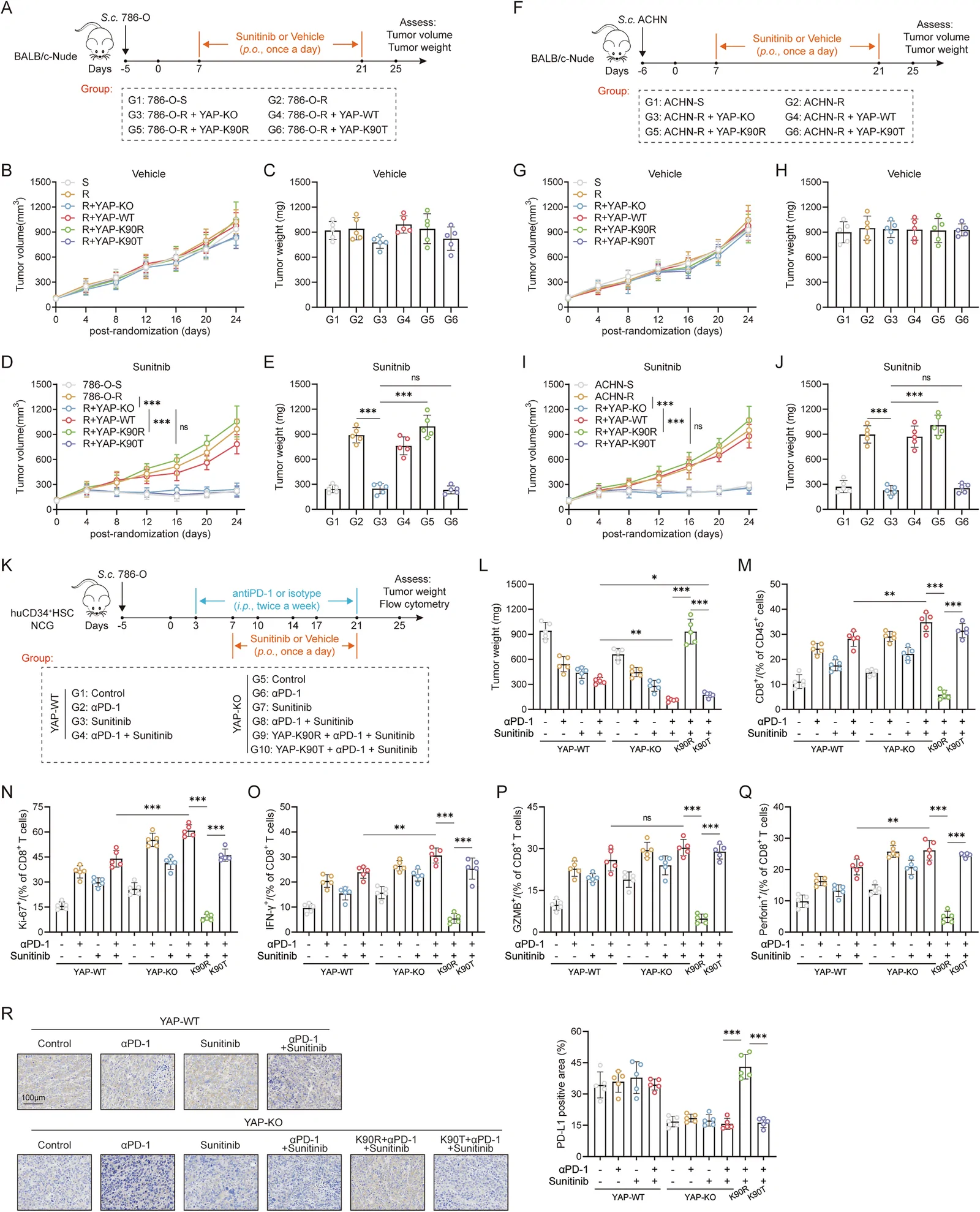
YAP-K90R remodels TIME and suppresses sunitinib response in RCC. **A** Schematic diagram depicting the treatment of BALB/c Nude mice bearing 786-O-derived tumors with sunitinib at the indicated time points. Measurement of tumor volume (**B**) and weight (**C**) after vehicle treatment in BALB/c nude mice bearing 786-O subcutaneous tumors. (*n* = 5). Measurement of tumor volume (**D**) and weight (**E**) after sunitinib treatment in BALB/c nude mice bearing 786-O subcutaneous tumors. (*n* = 5). **F** Schematic diagram depicting the treatment of BALB/c Nude mice bearing ACHN-derived tumors with sunitinib at the indicated time points. Measurement of tumor volume (**G**) and weight (**H**) after vehicle treatment in BALB/c nude mice bearing ACHN subcutaneous tumors. (*n* = 5). Measurement of tumor volume (**I**) and weight (**J**) after sunitinib treatment in BALB/c nude mice bearing ACHN subcutaneous tumors. (*n* = 5). **K** Schematic diagram depicting the treatment of huCD34^+^HSC NCG mice bearing 786-O-derived tumors with anti-PD-1 antibody or/and sunitinib at the indicated time points. **L** Measurement of subcutaneous tumor weight in huCD34^+^HSC NCG mice bearing 786-O cell-derived tumors. (*n* = 5). **M** Flow cytometric analysis of the percentage of CD8⁺ cells among CD45⁺ cells in subcutaneous tumors derived from the 786-O cell line in huCD34^+^HSC NCG mice. Flow cytometric analysis of the proportions of Ki-67⁺ (**N**), IFN-γ⁺ (**O**), GZMB⁺ (**P**), and perforin⁺ (**Q)** cells within the CD8⁺ T cell population in 786-O-derived subcutaneous tumors from huCD34^+^HSC NCG mice. **R** Representative images (left) and quantification (right) of PD-L1 expression in tumors. The statistical data in **B**, **D**, **G**, and **I** are analyzed using two-way ANOVA followed by multiple comparisons test. The statistical data in **C**, **E**, **H**, **J**, and **L**–**R** are analyzed using one-way ANOVA followed by multiple comparisons test. ns, not significant; *p < 0.05, **p < 0.01, ***p < 0.001.

To establish the specific role of YAP-K90 lactylation in targeted therapy sensitivity, we conducted an in vivo rescue experiment. Compared to the resistant lines (786-O-R and ACHN-R), knocking out YAP indeed suppressed their resistance, as expected. Crucially, re-expressing the delactylation-mimetic mutant YAP-K90R restored the resistant phenotype. Conversely, re-expressing the lactylation-mimetic mutant YAP-K90T did not (Fig. 6A–J). This provides direct evidence that the delactylated state of YAP-K90 is a key driver of targeted therapy resistance in RCC. As described above, YAP mediates the increased expression of PD-L1 in RCC cells. PD-L1 is indeed a critical immune checkpoint molecule. It binds to programmed death receptor-1 (PD-1), inhibiting T cell activation and thereby helping tumor cells evade the immune system’s attack. ICIs work by blocking the PD-1/PD-L1 axis or other checkpoint pathways, lifting this immunosuppressive state and restoring T cell anti-tumor function [42–44]. Compared to VEGFR-TKI monotherapy, the combination of VEGFR-TKI and PD-1 antibody treatment in mRCC patients results in greater benefits in terms of objective response rate and progression-free survival [45, 46], but treatment rates remained unsatisfactory. Having established the role of YAP-K90 lactylation, we therefore investigated whether modulating this modification could improve the efficacy of anti-PD-1 and targeted therapy in RCC. Using the huCD34^+^HSC NCG mouse model subcutaneously implanted with RCC cells expressing different YAP mutants, we evaluated the therapeutic outcomes (Fig. 6K and S7A). The combination therapy showed limited efficacy against tumors expressing the delactylation-mimetic YAP-K90R mutant. In stark contrast, tumors expressing the lactylation-mimetic YAP-K90T mutant exhibited a significantly enhanced response, as evidenced by reduced tumor weight (Fig. 6L and S7B). Flow cytometry analysis of tumor-infiltrating lymphocytes revealed that the YAP-K90R mutation suppressed CD8⁺ T cell function, indicated by reduced proportions, lower Ki-67 (proliferation), and decreased production of IFN-γ, GZMB, and perforin, while the YAP-K90T mutation maintained robust T cell activity (Fig. 6M–Q and S7C–S7G). Consistent with these findings, PD-L1 expression was decreased in the YAP-K90T group (Fig. 6R). These findings are consistent with our mechanistic studies demonstrating that YAP-K90 delactylation promotes PD-L1 expression and suppresses CD8⁺ T cell function. In summary, these findings demonstrate that maintaining YAP-K90 lactylation potently sensitizes RCC to the combination of targeted therapy and anti-PD-1 immunotherapy.

### HDAC1 inhibitor potentiates combination therapy and overcomes resistance in RCC

**Fig. 7:**
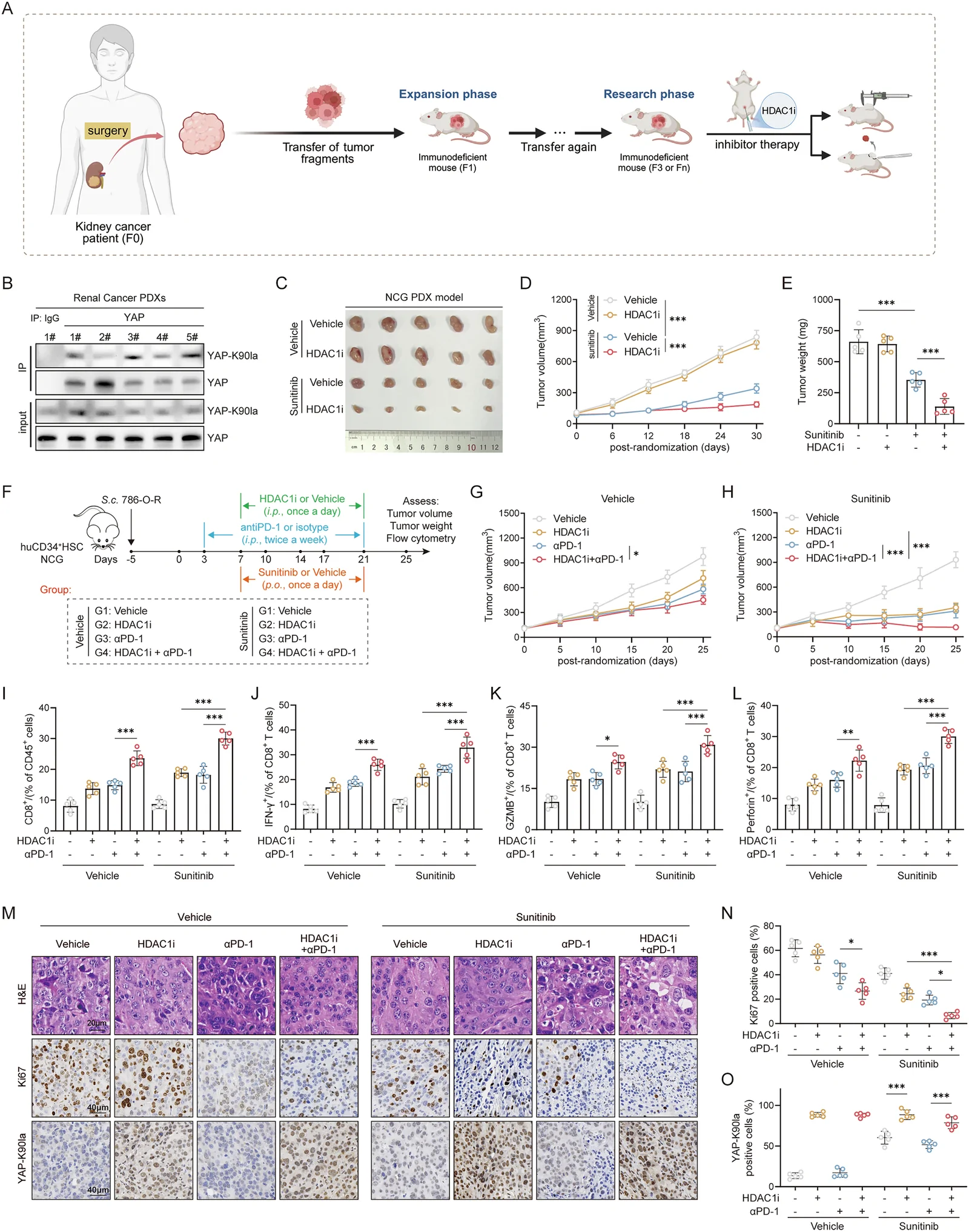
HDAC1 inhibitor potentiates combination therapy and overcomes resistance in RCC. **A** Schematic diagram of the construction of the patients derived xenograft (PDX). Created with BioRender.com. **B** Immunoblot analysis of PDXs in 5 patients with RCC. **C** Tumor images of NCG mice transplanted subcutaneously with PDX. (*n* = 5). Tumor volume measurements (**D**) and weight measurements (**E**) from Fig. 7C tumor images. **F** Schematic diagram of the treatment schedule with anti-PD-1 antibody, sunitinib, and/or HDAC1i at indicated time points in huCD34^+^HSC NCG mice bearing 786-O-R-derived tumors. Measurement of tumor volume after vehicle (**G**) or sunitinib (**H**) treatment in huCD34^+^HSC NCG mice bearing 786-O-R subcutaneous tumors. (*n* = 5). **I** Flow cytometric analysis of the percentage of CD8⁺ cells among CD45⁺ cells in subcutaneous tumors derived from the 786-O-R cell line in huCD34^+^HSC NCG mice. Flow cytometric analysis of the proportions of IFN-γ⁺ (**J**), GZMB⁺ (**K**), and perforin⁺ (**L)** cells within the CD8⁺ T cell population in 786-O-R-derived subcutaneous tumors from huCD34^+^HSC NCG mice. **M** Representative images of H&E, Ki-67 and YAP-K90la staining. Quantitative Ki-67 (**N**) and YAP-K90la (**O**) staining. The statistical data in **D**, **G**, and **H** are analyzed using two-way ANOVA followed by multiple comparisons test. The statistical data in **E**, **I**–**L**, **N**, and **O** are analyzed using one-way ANOVA followed by multiple comparisons test. *p < 0.05, **p < 0.01, ***p < 0.001.

Various combination strategies, including targeted therapy and immune checkpoint blockade, are being actively explored to improve treatment outcomes in RCC [47–49]. To further evaluate the functional significance of YAP-K90 lactylation in targeted therapy responsiveness, dose–response assays were performed in both sunitinib-resistant and parental RCC cells. In 786-O-R cells, Both lactate supplementation and HDAC1 inhibition reduced the IC50 of sunitinib, whereas the combination treatment produced the greatest sensitizing effect (Fig. S8A). Notably, lactate treatment failed to further enhance sunitinib sensitivity in YAP-K90T-expressing cells and YAP-K90R-expressing cells, indicating that the sensitizing effect of lactate is primarily mediated through the YAP-K90 site (Fig. S8B). Similar results were observed following HDAC1 inhibition or HDAC1 overexpression, where K90T consistently exhibited enhanced sensitivity whereas K90R displayed a resistant phenotype (Figs. S8C and S8D), further supporting YAP-K90 as the principal functional target of HDAC1-mediated regulation. Collectively, these findings indicate that YAP-K90 lactylation enhances sensitivity to sunitinib, whereas HDAC1-mediated delactylation promotes therapeutic resistance in RCC cells. To definitively confirm the role of the YAP lactylation status in the response to sunitinib, we screened five RCC PDX models and selected the one with the lowest YAP-K90la level for in vivo therapeutic experiments (Fig. 7A, B). HDAC1i significantly enhanced the efficacy of sunitinib against this YAP-K90la-low PDX model (Fig. 7C–E). Subsequently, we validated the therapeutic potential in a huCD34^+^HSC NCG mice subcutaneously engrafted with RCC cells (Fig. 7F and S8E). The triple combination of HDAC1i, sunitinib, and anti-PD-1 yielded the most potent anti-tumor response (Fig. 7G, H and S8F, S8G). Flow cytometry analysis confirmed that this regimen resulted in the highest levels of CD8⁺ T cell infiltration and activation (Fig. 7I–L and S8H–S8K), alongside the most suppressed tumor proliferation (Fig. 7M–O). Collectively, these findings identify HDAC1 as a therapeutically actionable regulator of YAP-K90 lactylation and provide a strong rationale for combining HDAC1 inhibition with targeted therapy and anti-PD-1 therapy to overcome resistance and enhance anti-tumor immunity in RCC.

## Discussion

The Warburg effect is a common phenomenon in most cancers and is the primary cause of lactate production [50]. This metabolic pathway is prevalent in tumor cells, but whether it affects the sensitivity of tumor cells to certain therapeutic drugs remains largely unknown. Recently, the novel PTMs of lysine lactylation mediated by lactate has been reported [9, 51–53]. However, the impact of this phenotype on tumors is still under investigation. Although previous studies have noted that lactate-mediated lactylation modifications are elevated in chemotherapy-resistant cancer cells, tumor metabolic reprogramming is complex and exhibits significant heterogeneity across different tumor states or sites [10, 11]. For example, basal-like breast tumors express low levels of glutamine synthetase (GLUL), in contrast to luminal tumor cells, which express GLUL [54]. Different subtypes of non-small cell lung cancer (NSCLC) also exhibit distinct metabolic phenotypes. Compared to lung adenocarcinoma, squamous cell carcinoma expresses higher levels of the glucose transporter GLUT1, shows higher uptake of the glucose analog FDG in clinical PET imaging, and is more sensitive to glucose uptake inhibitors [55]. In NSCLC, the 13C-glucose labeling pattern varies between tumors and across different regions within the same tumor [56]. In addition, Wang and his colleagues reported that the metabolic shift from tumor fatty acid metabolism to glycolysis increased the chemotherapy sensitivity of cisplatin-resistant cells [57]. Tumor cell metabolism is not rigid. Studies have shown that EGFR activation increases lactylation, which decreases after TKI treatment [58, 59]. Here, we found that targeted therapy treatment suppressed glycolytic activity in RCC cells, which may be a major factor affecting their drug responsiveness.

YAP, as a key effector protein of the Hippo signaling pathway, is typically retained in the cytoplasm when the pathway is active, limiting its activity [21]. When the Hippo pathway is inhibited, YAP is dephosphorylated and translocates to the nucleus, where it binds to transcription factors (such as TEADs) to regulate gene expression, thereby promoting cell proliferation and inhibiting apoptosis [28]. Reports have indicated that SET1A methylates YAP at the K342 site, leading to nuclear retention and increased YAP activity [60]. The activity of YAP is influenced by PTMs. Previous studies have primarily focused on YAP phosphorylation. For instance, CDK7 phosphorylates nuclear YAP at S127 and S397, enhancing its transcriptional function [61]. The viral-activated kinase IKKɛ phosphorylates YAP at the Ser403 site, promoting its degradation within the lysosome [62]. In addition to phosphorylation, YAP is also regulated by O-GlcNAcylation [63] and methylation [60, 64]. Previous studies by Zhou et al. found that lactylation promotes the nuclear localization of the YAP-TEAD complex [65], enhancing its transcriptional activity, whereas our study demonstrates that YAP delactylation promotes its nuclear localization and regulates its function. Although these two findings may seem contradictory, we believe the discrepancy may be due to differences in disease context, cell types, and the distinct roles of YAP lactylation and delactylation under various physiological or pathological conditions. Similar to how p53 acetylation promotes its interaction with co-activators, enhancing p53’s transcriptional activity and activating genes related to cell cycle arrest and apoptosis [66], other studies have shown that Sirt1-mediated deacetylation of p53 promotes its role in DNA damage response, activating genes related to repair [67]. Therefore, we propose that lactylation and delactylation may play distinct roles in different biological environments. Lactylation may promote YAP nuclear translocation under certain conditions, while delactylation could enhance YAP’s functional activation in other contexts. Differences in cell types and disease context may also contribute to these distinct effects, suggesting that lactylation and delactylation may regulate YAP’s nuclear translocation and function in different cellular contexts. In our study, we provide compelling evidence that, in addition to O-GlcNAcylation and methylation, lactylation is a key regulatory mechanism for YAP function in both the cytoplasm and nucleus. We demonstrate that HDAC1-mediated delactylation in renal cancer cells regulates YAP’s nuclear localization, leading to resistance to targeted therapy. In contrast, Zhou et al. focused on gastric cancer cells. This suggests that lactylation and delactylation may be dynamic processes, depending on the cell type and metabolic state. Under certain conditions, lactylation may facilitate YAP nuclear localization, while in other contexts, delactylation may promote YAP nuclear translocation. Therefore, the effects of these modifications are likely adjustable, depending on the specific needs and microenvironment of the cells. Interestingly, while our study demonstrates that YAP lactylation suppresses its nuclear localization and oncogenic function in RCC, previous studies suggest that YAP can exhibit context-dependent roles in cancer. For example, Wu et al reported that YAP silencing promotes metastasis in small cell lung cancer (SCLC), and entinostat, an HDAC1/3 inhibitor, restores YAP activity and suppresses metastatic traits [68]. These findings suggest that YAP may function either as an oncogene or a metastasis suppressor depending on the cellular context, tumor type, and epigenetic modifications. In RCC, our data indicate that HDAC1-mediated delactylation of YAP contributes to targeted therapy resistance and immune evasion, highlighting a tumor-specific mechanism in which modulating YAP lactylation could offer therapeutic benefit.

Interestingly, although YAP-K90 lactylation markedly regulated the interaction between YAP and LATS1, K90 is located outside the previously reported LATS1-binding region (amino acids 150–300) [34]. Consistent with this observation, modulation of HDAC1 expression or lactate levels did not affect the interaction between LATS1 and the isolated YAP(150–300 aa) fragment, suggesting that K90 lactylation does not directly alter the intrinsic LATS1-binding interface. Instead, K90 lactylation may influence YAP–LATS1 association through conformational changes or the recruitment of additional regulatory factors. Notably, K90 is positioned within the TEAD-binding region of YAP [35], raising the possibility that lactylation at this residue may also regulate the formation of transcriptionally active nuclear complexes. Recent studies have demonstrated that YAP cooperates with nuclear actin and TEAD4 to form a ternary transcriptional complex that drives target gene expression [36]. In line with this model, we found that the non-lactylatable mutant YAP-K90R exhibited enhanced interactions with both nuclear actin and TEAD4, whereas the lactylation-mimetic mutant YAP-K90T showed markedly reduced binding, accompanied by corresponding changes in TEAD-dependent transcriptional activity. These findings suggest that YAP-K90 delactylation not only facilitates YAP nuclear accumulation by weakening LATS1-dependent phosphorylation and subsequent 14-3-3 sequestration but also promotes the assembly of the YAP–actin–TEAD4 transcriptional complex, thereby maximizing YAP transcriptional activity. Because YAP-K90 lactylation also affects YAP phosphorylation, we cannot completely exclude the possibility that some of these effects are partially influenced by phosphorylation-dependent mechanisms. Nevertheless, our findings identify YAP-K90 lactylation as a regulatory mechanism that coordinates YAP phosphorylation, subcellular localization, and transcriptional complex formation. Collectively, these results support a model in which HDAC1 functions as a YAP de-lactylase that removes K90 lactylation, thereby reducing LATS1-mediated phosphorylation and 14-3-3 sequestration, promoting YAP nuclear localization, facilitating YAP–actin–TEAD4 complex assembly, and ultimately driving YAP-dependent transcription, PD-L1 expression, tumor progression, and therapeutic resistance in RCC.

HDAC1 is a well-known histone deacetylase that removes acetyl groups from lysine residues on histones, altering the charge and resulting in a more compact chromatin structure, thereby repressing gene transcription [69]. We discovered that HDAC1 also functions as a delactylase, inducing the delactylation of YAP. This finding aligns with previous studies showing that HDAC1-3 globally reduces histone lactylation levels [24]. In our study, we found that HDAC1-mediated YAP delactylation cooperates with the reduction of inhibitory YAP phosphorylation to enhance YAP activity and downstream biological functions. HDAC1 is overexpressed in various cancers and is often associated with tumor proliferation, invasion, and resistance, such as in lung and renal cancers [69, 70]. Moreover, HDAC inhibitors (e.g., Vorinostat and Entinostat) have been approved for the treatment of certain cancers, inducing cell cycle arrest and apoptosis by inhibiting HDAC1 [71–73]. A phase II clinical study demonstrated that NSCLC patients with high E-cadherin expression had significantly longer overall survival (OS) when treated with a combination of Erlotinib and Entinostat compared to Erlotinib monotherapy [74]. Similarly, the combination of vorinostat and EGFR-TKI has been shown to overcome EGFR-TKI resistance mediated by BIM polymorphism in patients with EGFR-mutant NSCLC [75]. However, studies investigating the role of HDAC inhibitors in RCC remain limited. In this study, we conducted in vivo experiments using an HDAC1 inhibitor and observed that its administration enhanced the responsiveness of RCC to combined targeted and immunotherapy. These findings highlight the promising preclinical potential of HDAC inhibitors in combination therapy for RCC.

RCC is closely associated with the functional loss of the VHL gene [76]. VHL inactivation leads to the accumulation of HIF and the activation of downstream signaling pathways. This gene’s functional loss causes abnormal regulation of the VEGF pathway, contributing to the highly vascular nature of advanced renal cancer [77]. Consequently, the anti-angiogenic effect of VEGFR TKIs plays a crucial role in the treatment of advanced RCC. However, resistance often develops after a period of targeted therapy [78], and the efficacy of combining TKIs with ICI therapy has also been unsatisfactory [46, 79]. The mechanisms of RCC resistance to TKIs are complex. The most direct mechanism is mutations in the drug targets of TKIs. Tumors can also induce resistance by reducing the intracellular concentration of TKIs, for instance, through enhanced drug efflux mediated by ATP-binding cassette (ABC) transporters, which expel drugs out of cells [33]. These transporters use energy from ATP hydrolysis to pump various substances, including chemotherapy drugs and TKIs, out of cells. This efflux mechanism reduces intracellular drug concentrations, leading to drug resistance in cancer cells [80]. Additionally, alterations in the tumor immune microenvironment promote immune evasion [81], thereby reducing the effectiveness of anti-PD-1 therapy. Our study demonstrates that HDAC1 is significantly elevated in TKI-resistant RCC tumors compared to their treatment-sensitive counterparts. Furthermore, the level of YAP lactylation at the K90 site is substantially reduced in these resistant tumors. Collectively, these findings indicate that HDAC1-mediated YAP-K90 delactylation is critically associated with a poor response to TKI therapy in RCC. This positions YAP-K90 as a promising prognostic biomarker and a potential therapeutic target. In this study, using an RCC PDX model characterized by low YAP-K90 lactylation, we demonstrated that an HDAC1 inhibitor promotes YAP K90 lactylation and significantly enhances the cancer-killing effect mediated by targeted therapy. Furthermore, in a huCD34^+^HSC NCG mouse subcutaneous tumor model, the HDAC1 inhibitor improved the efficacy of the combination therapy involving targeted therapy and anti-PD-1. Specifically, it enhanced the cytotoxic activity of CD8⁺ T cells and markedly potentiated targeted therapy-mediated tumor cell killing in vivo. However, a limitation of this approach is that HDAC1 inhibitors may broadly affect the deacetylation of both histone and non-histone proteins, which could lead to nonspecific alterations in gene expression and an increased risk of off-target effects. Ideally, a strategy that specifically promotes lactylation at the YAP K90 site would be more favorable for precise therapeutic intervention.

**Fig. 8:**
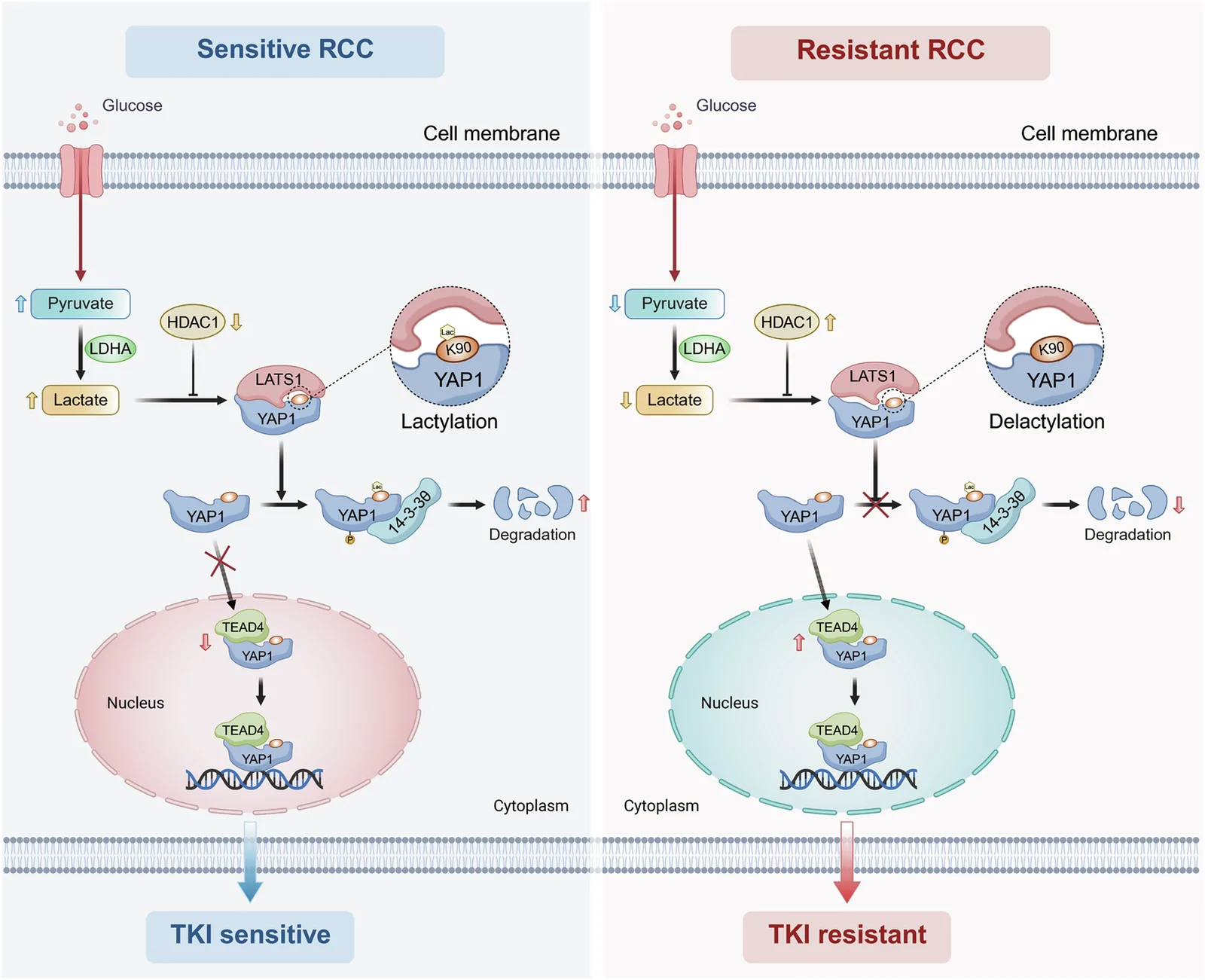
A model for the role of YAP delactylation mediated by HDAC1 in regulating its nuclear localization and promoting tumor resistance. Created with BioRender.com.

Overall, our findings support a model in which HDAC1-mediated YAP K90 delactylation promotes YAP activation through multiple coordinated mechanisms. In the cytoplasm, K90 delactylation is associated with reduced YAP phosphorylation and diminished interactions with LATS1 and 14-3-3θ, thereby facilitating YAP nuclear accumulation. However, whether HDAC1/K90 lactylation-dependent regulation of YAP phosphorylation converges entirely with the canonical LATS1-mediated phosphorylation pathway remains to be fully elucidated. In addition, our findings suggest that YAP-K90 delactylation not only promotes YAP nuclear accumulation but also facilitates assembly of the YAP–actin–TEAD4 transcriptional complex, thereby providing an additional layer of regulation for YAP transcriptional activation. Consequently, YAP activates a transcriptional program involving EMT-associated genes and PD-L1, ultimately promoting tumor progression, immune evasion, and targeted therapy resistance **(**Fig. 8**)**. Collectively, these findings identify HDAC1 as a critical upstream regulator of YAP signaling and suggest that therapeutic targeting of the HDAC1–YAP K90 delactylation axis may represent a promising strategy for overcoming resistance to both targeted therapy and immunotherapy in renal cell carcinoma.

One limitation of this study is that with the rapid evolution of therapeutic strategies, combination therapies involving TKIs and immune checkpoint inhibitors have become the preferred first-line regimen for ccRCC. But, clinically used doses are often lower than the concentration employed in our study to establish the resistant model and investigate the underlying mechanisms. Although our initial discoveries were made under higher drug concentrations, we are confident that the pathway we identified, involving lactylation, represents a genuine and mechanistically significant survival mechanism. It is plausible that this same pathway contributes to adaptive resistance and relapse during the chronic, lower-dose exposure seen in patients. Ideally, future investigations should focus on studying the relationship between YAP delactylation and the development of adaptive resistance under conditions of chronic, low-dose therapy. In addition, although our data support a role for YAP-K90 lactylation in regulating YAP–actin–TEAD4 complex assembly, the precise structural mechanism by which K90 lactylation influences these interactions remains to be determined.

## Materials and methods

### Human studies

The human studies were approved by the Clinical Research and Experimental Animal Ethics Committee of the First Affiliated Hospital of Sun Yat-sen University, and written informed consent was obtained from all participants. Tumor specimens were collected from 40 patients with RCC who experienced disease recurrence or metastasis after primary tumor resection and were subsequently treated with TKI targeted therapy. Based on the clinical response to TKI treatment, patients were classified into sensitive and resistant groups.

### Cell lines and cell culture

The YAP KO cell line was generated using gene targeting by CRISPR/Cas9. HEK293T, human renal cancer cell line 786-O and ACHN were obtained from American Type Culture Collection (ATCC). The cell lines were cultured in a humidified incubator at 37 °C with 5% CO2. The culture medium was supplemented with 10% fetal bovine serum and 1% penicillin-streptomycin. 786-O cells were cultured in RPMI 1640 medium (Gibco), while HEK293T and ACHN cells were cultured in DMEM medium (Gibco). Vendor-conducted validations encompassed short tandem repeat (STR) profiling and routine Mycoplasma contamination testing for every cell line.

### Establishment of sunitinib-resistant RCC cell lines

To establish sunitinib-resistant RCC cell lines, a xenograft mouse model was generated using 4–6 week-old male NCG mice maintained under specific pathogen-free (SPF) conditions. A total of 5 × 10⁶ 786-O or ACHN cells suspended in 100 μL were subcutaneously injected into the flanks of mice. Tumor volume was calculated using the formula length × width² / 2 and measured weekly. When tumors reached approximately 200 mm³, first-generation mice were treated with sunitinib (40 mg/kg/day, Selleck) or vehicle control by oral gavage following a standard schedule (4 weeks on treatment and 2 weeks off). Sunitinib was dissolved in 5% DMSO and corn oil. After 6 weeks, tumors were harvested, dissected into ~1 mm³ fragments, and transplanted into second-generation mice, which were subjected to the same treatment regimen. This process was repeated for a third generation of mice. Tumors from third-generation mice were collected and mechanically dissociated, followed by washing with cold PBS containing 2% penicillin–streptomycin and levofloxacin (100 μg/mL). The tissues were then digested in DMEM containing 0.2% collagenase IV (Stemcell), 0.01% hyaluronidase (Stemcell), and 0.002% DNase I (Stemcell) at 37 °C with continuous agitation for 45 min. The resulting cell suspension was centrifuged at 300 × *g*, and cells were resuspended and plated at a density of 7 × 10⁵ cells per well in 6-well plates. Fibroblasts were removed by repeated differential adhesion. The established sunitinib-resistant RCC cell lines were designated as 786-O-R and ACHN-R.

### Animals

All animal experiments are conducted in accordance with the provisions of the Sun Yat-sen University Animal Protection and Use Committee. Male BALB/c nude (4–6 weeks old), NCG mice (4–6 weeks old) and huCD34^+^HSC NCG mice (4–6 weeks old) were purchased from GemPharmatech, China. The mice were fed for 1 week without specific pathogens in the laboratory animal room of the Cancer Prevention and Control Center of Sun Yat-sen University. The animal room has a controlled temperature (18–23 °C), humidity (40-60%) and a 12 h light /12 h dark cycle.

### Animal experiments

This study conducted five in vivo experiments. No statistical methods were used to predetermine the sample size. No data was excluded and the investigator was blinded to the group allocation during the experiment. In all animal experiments, tumor volume was calculated using the formula: length × width × width/2.

In Experiment 1, 786-O or ACHN cells (expressing YAP-WT, YAP-K90R, or YAP-K90T) were injected subcutaneously into the right flank of huCD34^+^HSC NCG mice. When tumor volumes reached approximately 100 mm³, anti-PD-1 antibody (BioXcell, 12.5 mg/kg, *i.p*.) or an isotype control was administered intraperitoneally twice a week for 2-3 weeks. Mice were euthanized on day 25 for tumor assessment and flow cytometric analyses.

In Experiment 2, 786-O or ACHN cells (including sensitive, resistant, and resistant cells overexpressing different YAP variants) were injected subcutaneously into the right flank of BALB/c nude mice. When tumor volumes reached approximately 100 mm³, mice were randomly divided into two main treatment groups: a vehicle control group and a sunitinib group (Selleck, 40 mg/kg/day, *p*.*o*.). Each main group was further subdivided into 6 subgroups (*n* = 5 per subgroup), resulting in a total of 12 groups (60 mice). The subgroups were as follows: Sensitive (S), Resistant (R), R + YAP-KO, R + YAP-WT, R + YAP-K90R and R + YAP-K90T. Treatment commenced in the second week and continued daily for two weeks. All mice were euthanized on day 25 for tumor assessment.

In Experiment 3, 786-O or ACHN cells (WT or YAP-KO) were subcutaneously injected into the right axilla of huCD34^+^ HSC NCG mice. When tumor volumes reached approximately 100 mm³, mice were randomly divided into 4 subgroups (*n* = 5): 1) control, 2) anti-PD-1 monotherapy, 3) sunitinib monotherapy, and 4) anti-PD-1+sunitinib combination. The mice bearing YAP-KO tumors were divided into 6 subgroups (*n* = 5): 1) control, 2) anti-PD-1, 3) sunitinib, 4) anti-PD-1+sunitinib, 5) anti-PD-1+sunitinib+YAP-K90R reconstitution, and 6) anti-PD-1+sunitinib+YAP-K90T reconstitution, totaling 50 mice. The treatment regimen was as follows: anti-PD-1 antibody (12.5 mg/kg, *i.p*., twice weekly) and/or sunitinib (40 mg/kg/day, *p*.*o*.) for two weeks. All mice were euthanized on day 25 for tumor assessment, followed by immunohistochemical and flow cytometric analyses.

In Experiment 4, patient-derived xenografts (PDXs) of renal cell carcinoma were subcutaneously implanted into 6-week-old NCG mice. When tumor volumes reached approximately 100 mm³, mice were randomly divided into four groups (*n* = 5): Vehicle, HDAC1i group (50 mg/kg/day, *i.p*.), sunitinib group (40 mg/kg/day, *p*.*o*.), and sunitinib+HDAC1i group. Tumor volume measurements and assessments were conducted according to the protocol for tumor xenograft experiments.

In Experiment 5, 786-O-R or ACHN-R cells were injected subcutaneously into the right axilla of huCD34^+^HSC NCG mice. When tumor volumes reached approximately 100 mm³, mice were randomly assigned to two main cohorts: a vehicle cohort and a sunitinib treatment cohort. Each main cohort was further divided into four subgroups (*n* = 5 per subgroup), resulting in a total of 8 groups (40 mice). The subgroups were: 1) Vehicle, 2) HDAC1i, 3) anti-PD-1, and 4) HDAC1i+anti-PD-1. All treatments were administered for two weeks. The dosing regimens were as follows: sunitinib (40 mg/kg/day, *p*.*o*.); anti-PD-1 antibody (12.5 mg/kg, *i.p*., twice weekly); and the HDAC1 inhibitor valproic acid (Sigma-Aldrich, 40 mg/kg/day, *i.p*.).

### RT-qPCR

Total RNA was extracted from tumor tissues or cell lines using TRIzol (Invitrogen). Reverse transcription of total RNA was performed using the Reverse Transcription Master Mix (EZBioscience). Quantitative RT-PCR (qRT-PCR) was conducted with SYBR Green Real-Time PCR Master Mix (EZBioscience) using a Roche LightCycler480 instrument. The amplification parameters were set at 95 °C for 30 s, 58 °C for 30 s, and 72 °C for 30 s (for a total of 35 cycles). The expression levels of all target genes were normalized to the control gene GAPDH or β-Actin. The qPCR primers are listed in Table S1.

### Lentiviral transduction and the generation of stable cell lines

Lentiviral particles were produced by transfecting HEK293T cells with transfer plasmids together with packaging plasmids (pCMV-VSVG, pMDLg-RRE, and pRSV-REV) using Lipofectamine 2000 (Invitrogen). Viral supernatants were collected at 48 h post-transfection and used immediately for infection or stored at −80 °C. To establish stable cell lines, viral supernatants were diluted 1:2 in complete culture medium containing 8 μg/mL polybrene and used to infect cells at approximately 80% confluence for 24 h. Cells were selected with puromycin (1–3 μg/mL) starting 48 h after infection for one week, followed by expansion and validation by immunoblotting. The shRNA constructs targeting HDAC1, YAP, TEAD2, and TEAD4, as well as overexpression constructs for HDAC family members (HDAC1–11), SIRT family members (SIRT1–7), and YAP, are listed in Table S1.

### Co-immunoprecipitation and Western blotting

Cells were lysed on ice with cell lysis buffer (Beyotime) containing protease inhibitors (CoWin BioSciences). After centrifugation at 12,000 *g* for 15 min, protein concentrations were measured using the Pierce BCA Protein Assay Kit (Thermo Fisher Scientific). Immunoprecipitation was performed using anti-FLAG magnetic beads (Selleck), anti-HA magnetic beads (Selleck) or various antibodies, incubating overnight at 4°C. The magnetic beads were washed three times with wash buffer and boiled for 10 min in 1×SDS sample buffer. Proteins were separated on a 7.5–12.5% SDS-PAGE gel. After electrophoresis, the proteins were transferred to a PVDF membrane (Merck Millipore) using a transfer unit (Tanon). The membrane was blocked with 5% milk in TBST buffer. After blocking, the PVDF membrane was incubated overnight at 4 °C with the primary antibody. Following a 1-hour incubation at room temperature with the secondary antibody, protein bands were detected using chemiluminescence (Tanon). Primary antibodies used in western blotting included rabbit anti-L-Lactyl Lysine (Jingjie PTM, Cat#PTM-1401RM), rabbit anti-Malonyllysine (Jingjie PTM, Cat#PTM-901), rabbit anti-Succinyllysine (Jingjie PTM, Cat#PTM-401), rabbit anti-Crotonyllysine (Jingjie PTM, Cat#PTM-501), rabbit anti-YAP1 (abcam, Cat#ab52771), rabbit anti-YAP1 (phospho S127) (abcam, Cat#ab76252), rabbit anti-HDAC1 (CST, Cat#34589), rabbit anti-LATS1 (CST, Cat#3477), rabbit anti-14-3-3 (proteintech, Cat#14503-1-AP), rabbit anti-N-cadherin (CST, Cat#13116), rabbit anti-SLUG (CST, Cat#9585), rabbit anti-ZEB1 (CST, Cat#70512), rabbit anti-MRP1/ABCC1 (CST, Cat#72202), rabbit anti-MDR1/ABCB1 (CST, Cat#13978), rabbit anti-TEAD2 (proteintech, Cat#21159-1-AP), rabbit anti-TEAD4 (proteintech, Cat#12418-1-AP), rabbit anti-PD-L1 (CST, Cat#13684), rabbit anti-FLAG (CST, Cat#14793), mouse anti-FLAG (CST, Cat#8146), rabbit anti-HA (CST, Cat#3724), mouse anti-HA (CST, Cat#2367), mouse anti-GAPDH (proteintech, Cat#60004-1-Ig), mouse anti-Alpha Tubulin (proteintech, Cat#66031-1-Ig), mouse anti-Lamin B1 (proteintech, Cat#66095-1-Ig). HRP-conjugated secondary antibodies included HRP-conjugated goat anti-rabbit IgG (proteintech, Cat#SA00001-2) and HRP-conjugated goat anti-mouse IgG (proteintech, Cat#SA00001-1).

### Immunofluorescence (IF) Staining

786-O and ACHN cells were transfected with plasmids or treated with various stimuli. The cells were then washed with PBS and fixed with 4% paraformaldehyde in PBS for 20 min. After permeabilization with 0.2% Triton X-100, the cells were blocked with 10% goat serum at room temperature for 1 h. Cells were incubated overnight with specific primary antibodies, followed by incubation with fluorescence dye-conjugated secondary antibodies. The nuclei were stained with DAPI (4’,6-diamidino-2-phenylindole; Sigma-Aldrich). Images were captured using a Zeiss LSM880 confocal microscope system (ZEN 2.3 (blue edition)) and analyzed with Zeiss confocal software. Nuclear localization was quantified using mean fluorescence intensity (MFI), and all images were acquired as single-plane images under identical imaging settings.

### Silver staining

Protein samples were loaded onto each lane of a 10% SDS-PAGE gel. After electrophoresis, the gel was stained using the Fast Silver Stain Kit (Beyotime).

### Colony formation assay

Seed the specified number of cells (500–1000) into each well of a six-well plate. After one day, expose the cells to sunitinib or various reagents, and incubate in a culture chamber for 7–10 days to allow colony formation. The colonies were stained with crystal violet and quantified.

### Lactate measurement

Intracellular lactate levels were measured using the L-Lactate Assay Kit (Abcam) following the manufacturer’s instructions.

### Metabolic assays

According to the protocol, the Seahorse XF96 Analyzer was used to measure the oxygen consumption rate (OCR) and extracellular acidification rate (ECAR) of cells. Tumor cells were seeded in XF96 microplates at a density of 1 × 10⁴ cells/well and incubated overnight. To assess cellular bioenergetics, glucose (10 mM), oligomycin (1.5 μM, Selleck), and 2-deoxyglucose (2-DG, 50 mM, Selleck) were sequentially added for ECAR measurements; or oligomycin (1.5 μM), carbonyl cyanide-4 (trifluoromethoxy) phenylhydrazone (FCCP, 0.5 μM, Selleck), and rotenone/antimycin A (Rot/AA, 0.5 μM) were added for OCR measurements. Data were normalized to cell number and analyzed using Wave software (Agilent).

### 4D label-free quantitative lactylproteomics analysis

Remove the culture medium from the cultured cells and wash them 2-3 times with pre-cooled PBS. Discard the supernatant and use a cell scraper to thoroughly scrape all cells from the culture vessel for cell counting. Transfer the cells to a 2 mL cryovial, flash freeze in liquid nitrogen, and store at -80 °C, ensuring sufficient dry ice for transport. Samples were sent to Jingjie PTM BioLabs for 4D label-free quantitative proteomics analysis, involving experimental procedures such as protein extraction, trypsin digestion, affinity enrichment, and LC-MS/MS analysis.

### Luciferase reporter assay

The promoter sequences were synthesized and cloned into the pGL4 luciferase reporter vector by Synbio Technologies (Soochow, China), and all constructs were verified by DNA sequencing. Recombinant pGL4 plasmids containing the promoter sequences or the empty pGL4 vector were co-transfected into human RCC cells together with the pRL Renilla luciferase plasmid (Promega), which served as an internal control. After 48 h of incubation, transcriptional activity was quantified using the Dual-Luciferase Reporter Assay System (Promega) according to the manufacturer’s instructions. Luminescence signals were measured using a Tecan SPARK multimode microplate reader. Firefly luciferase activity was subsequently normalized to the corresponding Renilla luciferase activity to correct for variations in transfection efficiency.

### Immunohistochemistry

Paraffin-embedded tissue sections were first baked at 56 °C for 30 min, followed by deparaffinization in xylene and rehydration through a graded ethanol series. Antigen retrieval was performed using citrate buffer under pressurized heat conditions. After cooling to room temperature, the sections were treated with 3% hydrogen peroxide for 10 min to block endogenous peroxidase activity. The sections were then incubated with normal goat serum blocking solution (ZSGB-BIO) at 37 °C for 60 min and subsequently incubated overnight at 4 °C with the primary antibody. After washing, the sections were incubated with the corresponding secondary antibody (ZSGB-BIO) at 37 °C for 1 h. The target proteins were visualized using 3,3′-diaminobenzidine (DAB; ZSGB-BIO), followed by counterstaining of nuclei with hematoxylin (Servicebio). Primary antibodies included rabbit anti-Ki67 (CST, Cat#9449), rabbit anti-PD-L1 (CST, Cat#13684), rabbit anti-YAP (abcam, Cat#ab52771), and rabbit anti-HDAC1 (CST, Cat#34589).

To evaluate antigen expression levels in tissue sections, H-score assessment was independently performed by two experienced pathologists based on the percentage of positively stained cells and staining intensity. The percentage score represented the proportion of positively stained cells, ranging from 0% to 100%. Staining intensity was graded on a scale of 0–3, where 0 indicated negative staining, 1 indicated weak staining, 2 indicated moderate staining, and 3 indicated strong staining. The final H-score was calculated using the following formula: H-score = (3 × percentage of strongly positive cells) + (2 × percentage of moderately positive cells) + (1 × percentage of weakly positive cells). The resulting H-score ranged from 0 to 300.

### Flow cytometry analysis

Subcutaneous tumors were harvested, minced into small fragments, and digested in DMEM containing 0.002% DNase I (Stemcell), 0.01% hyaluronidase (Stemcell), 0.2% collagenase IV (Stemcell), and 3 mM CaCl₂ (Sigma-Aldrich) at 37 °C with continuous agitation for 60 min. The digested tissues were centrifuged at 300 × *g*, and the resulting cell suspension was filtered through cell strainer to obtain single-cell suspensions. To block Fc receptors, cells were incubated with Human BD Fc Block (BD Biosciences) for human immune cells or purified rat anti-mouse CD16/CD32 antibody (BD Biosciences) for mouse immune cells. For intracellular staining of IFN-γ, GZMB, and Perforin, single-cell suspensions were stimulated with 50 ng/mL phorbol 12-myristate 13-acetate (PMA; Sigma-Aldrich) and 1 μg/mL ionomycin (Sigma-Aldrich) for 5 h at 37 °C in a humidified incubator. To facilitate intracellular cytokine accumulation, Brefeldin A (BFA, Sigma-Aldrich) was added according to the manufacturer’s instructions during stimulation. Following viability staining, Fc receptor blocking, and surface marker staining, cells were fixed and permeabilized using the BD Cytofix/Cytoperm Kit (BD Biosciences) according to the manufacturer’s instructions. Intracellular staining was then performed in 1× BD Perm/Wash buffer at 4 °C for 30 min. After each staining step, cells were washed twice with FACS buffer and collected by centrifugation. Fluorescence signals were acquired using a CytoFLEX flow cytometer (Beckman), and data were analyzed with FlowJo software (v10.5.3). The antibodies used for flow cytometry analysis include Zombie Aqua™ Fixable Viability Kit (BioLegend, Cat#423101), PE/Cyanine7 anti-human CD45 (BioLegend, Cat#304015), APC/Cyanine7 anti-human CD3 (BioLegend, Cat#300317), FITC anti-human CD8 (BioLegend, Cat#344703), APC anti-human IFN-γ (BioLegend, Cat#502511), PE anti-human/mouse Granzyme B Recombinant (BioLegend, Cat#372207), Brilliant Violet 421™ anti-mouse/human Ki-67 (BioLegend, Cat#151208), and PerCP/Cyanine5.5 anti-human Perforin (BioLegend, Cat#308113).

### Cell proliferation assay

Cell proliferation was assessed using the Cell Counting Kit-8 (CCK-8, MCE) according to the manufacturer’s instructions. Cells were seeded at 2000 or 4000 cells per well in a 96-well cell culture plate. After 1 day, complete culture medium containing various concentrations of sunitinib was added and incubated for 2 days. Subsequently, 10 μL of CCK-8 reagent was added to each well at the designated time points. After incubation at 37 °C for 1 h the absorbance was measured at OD 450 nm, and growth curves were generated to calculate the half maximal inhibitory concentration (IC50).

### Isolation and co-culture of human CD8^+^ T cells

Human peripheral blood mononuclear cells (PBMCs) were isolated from healthy donor buffy coats by density gradient centrifugation. CD8⁺ T cells were subsequently purified from PBMCs by positive selection using human CD8 MicroBeads and an automated magnetic-activated cell sorting (MACS) system (Miltenyi Biotec). The isolated CD8⁺ T cells were activated with Human T-Activator CD3/CD28 Dynabeads for 48–72 h before co-culture. For the co-culture assay, activated CD8⁺ T cells were co-cultured with target RCC cells (786-O or ACHN) for 12–24 ho.

### Statistical analysis

Data are presented as mean ± standard deviation, and statistical analyses were performed using unpaired two-tailed Student’s *t*-test for two-group comparisons and one-way or two-way ANOVA with appropriate post hoc tests for multiple-group comparisons. All in vitro experiments were conducted with at least three independent biological replicates. The exact value of *n*, representing the number of experimental mice, is indicated in the legends. All tests were two-tailed, with a *p*-value of <0.05 considered statistically significant. Statistical analysis was performed using GraphPad Prism (v 9.5.1).

## Supplementary information

Supplementary figures and tables

WB original image

## Data Availability

All data generated in this study are available upon request by contacting the corresponding author.

## References

[1] Siegel RL, Giaquinto AN, Jemal A. Cancer statistics, 2024. CA A Cancer J Clinicians. 2024;74:12–49. 10.3322/caac.21820 38230766

[2] Capitanio U, Montorsi F. Renal cancer. Lancet. 2016;387:894–906. 10.1016/S0140-6736(15)00046-X 26318520

[3] Barata PC, Rini BI. Treatment of renal cell carcinoma: Current status and future directions. CA A Cancer J Clinicians. 2017;67:507–24. 10.3322/caac.21411 28961310

[4] Motzer RJ, Jonasch E, Boyle S, Carlo MI, Manley B, Agarwal N, et al. Kidney Cancer, Version 1.2021: Featured Updates to the NCCN Guidelines. J Natl Compr Cancer Netw: JNCCN. 2020;18:1160. 10.6004/jnccn.2020.0043 PMC10191771 32886895

[5] McDermott DF, Huseni MA, Atkins MB, Motzer RJ, Rini BI, Escudier B, et al. Clinical activity and molecular correlates of response to atezolizumab alone or in combination with bevacizumab versus sunitinib in renal cell carcinoma. Nat Med 2018;24:749. 10.1038/s41591-018-0053-3 PMC6721896 29867230

[6] Braun DA, Bakouny Z, Hirsch L, Flippot R, Allen EV, Wu CJ, et al. Beyond Conventional Immune Checkpoint Inhibition: Renal Cell Carcinoma at the Forefront of Solid Tumor Immunology. Nat Rev Clin Oncol. 2021;18:199. 10.1038/s41571-020-00455-z PMC8317018 33437048

[7] Heiden MGV, Cantley LC, Thompson CB. Understanding the Warburg Effect: The Metabolic Requirements of Cell Proliferation. Science (N Y, NY). 2009;324:1029. 10.1126/science.1160809 PMC2849637 19460998

[8] Kim J, DeBerardinis RJ. Mechanisms and implications of metabolic heterogeneity in cancer. Cell Metab. 2019;30:434. 10.1016/j.cmet.2019.08.013 PMC6730674 31484055

[9] Zhang D, Tang Z, Huang H, Zhou G, Cui C, Weng Y, et al. Metabolic regulation of gene expression by histone lactylation. Nature. 2019;574:575–80. 10.1038/s41586-019-1678-1 PMC6818755 31645732

[10] Chen Y, Wu J, Zhai L, Zhang T, Yin H, Gao H, et al. Metabolic regulation of homologous recombination repair by MRE11 lactylation. Cell. 2024;187:294–311. 10.1016/j.cell.2023.11.022 PMC11725302 38128537

[11] Chen H, Li Y, Li H, Chen X, Fu H, Mao D, et al. NBS1 lactylation is required for efficient DNA repair and chemotherapy resistance. Nature. 2024;631:663. 10.1038/s41586-024-07620-9 PMC11254748 38961290

[12] Leo AD, Ugolini A, Yu X, Scirocchi F, Scocozza D, Peixoto B, et al. Glucose-driven histone lactylation promotes the immunosuppressive activity of monocyte-derived macrophages in glioblastoma. Immunity. 2024;57:1105–23.e8. 10.1016/j.immuni.2024.04.006 PMC11114377 38703775

[13] Wang Y, Li H, Jiang S, Fu D, Lu X, Lu M, et al. The glycolytic enzyme PFKFB3 drives kidney fibrosis through promoting histone lactylation-mediated NF-κB family activation. Kidney Int. 2024;106:226–40. 10.1016/j.kint.2024.04.016 38789037

[14] Ma W, Jia K, Cheng H, Xu H, Li Z, Zhang H, et al. Orphan Nuclear Receptor NR4A3 Promotes Vascular Calcification via Histone Lactylation. Circulation Res. 2024;134:1427–47. 10.1161/CIRCRESAHA.123.323699 38629274

[15] Li F, Si W, Xia L, Yin D, Wei T, Tao M, et al. Positive feedback regulation between glycolysis and histone lactylation drives oncogenesis in pancreatic ductal adenocarcinoma. Mol Cancer. 2024;23:90. 10.1186/s12943-024-02008-9 PMC11071201 38711083

[16] Li F, Zhang H, Huang Y, Li D, Zheng Z, Xie K, et al. Single-cell transcriptome analysis reveals the association between histone lactylation and cisplatin resistance in bladder cancer. Drug Resistance Updates. 2024;73:101059. 10.1016/j.drup.2024.101059 38295753

[17] Li G, Wang D, Zhai Y, Pan C, Zhang J, Wang C, et al. Glycometabolic reprogramming-induced XRCC1 lactylation confers therapeutic resistance in ALDH1A3-overexpressing glioblastoma. Cell Metab. 2024;36:1696–710.e10. 10.1016/j.cmet.2024.07.011 39111285

[18] Tan M, Luo H, Lee S, Jin F, Yang JS, Montellier E, et al. Identification of 67 histone marks and histone lysine crotonylation as a new type of histone modification. Cell. 2011;146:1016–28. 10.1016/j.cell.2011.08.008 PMC3176443 21925322

[19] Zhang Z, Tan M, Xie Z, Dai L, Chen Y, Zhao Y. Identification of lysine succinylation as a new post-translational modification. Nat Chem Biol. 2011;7:58–63. 10.1038/nchembio.495 PMC3065206 21151122

[20] Peng C, Lu Z, Xie Z, Cheng Z, Chen Y, Tan M, et al. The First Identification of Lysine Malonylation Substrates and Its Regulatory Enzyme. Molecular Cellular Proteomics; 10. Epub ahead of print 1 December 2011. 10.1074/mcp.M111.012658. PMC3237090 21908771

[21] Fu M, Hu Y, Lan T, Guan K-L, Luo T, Luo M. The Hippo signalling pathway and its implications in human health and diseases. Sig Transduct Target Ther. 2022;7:1–20. 10.1038/s41392-022-01191-9 PMC9643504 36347846

[22] Sabari BR, Zhang D, Allis CD, Zhao Y. Metabolic regulation of gene expression through histone acylations. Nat Rev Mol Cell Biol. 2017;18:90–101. 10.1038/nrm.2016.140 PMC5320945 27924077

[23] Wang C, Huang M, Lin Y, Zhang Y, Pan J, Jiang C, et al. ENO2-derived phosphoenolpyruvate functions as an endogenous inhibitor of HDAC1 and confers resistance to antiangiogenic therapy. Nat Metab. 2023;5:1765–86. 10.1038/s42255-023-00883-y 37667133

[24] Moreno-Yruela C, Zhang D, Wei W, Bæk M, Liu W, Gao J, et al. Class I histone deacetylases (HDAC1–3) are histone lysine delactylases. Sci Adv. 2022;8:eabi6696. 10.1126/sciadv.abi6696 PMC8769552 35044827

[25] Xiong J, He J, Zhu J, Pan J, Liao W, Ye H, et al. Lactylation-driven METTL3-mediated RNA m6A modification promotes immunosuppression of tumor-infiltrating myeloid cells. Mol Cell. 2022;82:1660–77.e10. 10.1016/j.molcel.2022.02.033 35320754

[26] Gao R, Li Y, Xu Z, Zhang F, Xu J, Hu Y, et al. Mitochondrial pyruvate carrier 1 regulates fatty acid synthase lactylation and mediates treatment of nonalcoholic fatty liver disease. Hepatology. 2023;78:1800–15. 10.1097/HEP.0000000000000279 36651176

[27] Motzer RJ, Robbins PB, Powles T, Albiges L, Haanen JB, Larkin J, et al. Avelumab plus axitinib versus sunitinib in advanced renal cell carcinoma: biomarker analysis of the phase 3 JAVELIN Renal 101 trial. Nat Med. 2020;26:1733. 10.1038/s41591-020-1044-8 PMC8493486 32895571

[28] Hagenbeek TJ, Zbieg JR, Hafner M, Mroue R, Lacap JA, Sodir NM, et al. An allosteric pan-TEAD inhibitor blocks oncogenic YAP/TAZ signaling and overcomes KRAS G12C inhibitor resistance. Nat Cancer. 2023;4:812–28. 10.1038/s43018-023-00577-0 PMC10293011 37277530

[29] Bennison SA, Blazejewski SM, Liu X, Hacohen-Kleiman G, Sragovich S, Zoidou S, et al. The cytoplasmic localization of ADNP through 14-3-3 promotes sex-dependent neuronal morphogenesis, cortical connectivity, and calcium signaling. Mol Psychiatry. 2023;28:1946–59. 10.1038/s41380-022-01939-3 36631597

[30] Currey L, Thor S, Piper M. TEAD family transcription factors in development and disease. Development. 2021;148:dev196675. 10.1242/dev.196675 34128986

[31] Nilsson MB, Yang Y, Heeke S, Patel SA, Poteete A, Udagawa H, et al. CD70 is a therapeutic target upregulated in EMT-associated EGFR tyrosine kinase inhibitor resistance. Cancer Cell. 2023;41:340–55.e6. 10.1016/j.ccell.2023.01.007 PMC10259078 36787696

[32] Lv M, Liu B, Duan Y, Lin J, Dai L, Li Y, et al. Engineered Biomimetic Nanovesicles Synergistically Remodel Folate-Nucleotide and γ-Aminobutyric Acid Metabolism to Overcome Sunitinib-Resistant Renal Cell Carcinoma. ACS Nano. 2024;18:27487–502. 10.1021/acsnano.4c08055 39329191

[33] Yang Y, Li S, Wang Y, Zhao Y, Li Q. Protein tyrosine kinase inhibitor resistance in malignant tumors: molecular mechanisms and future perspective. Signal Transduct Target Ther. 2022;7:329. 10.1038/s41392-022-01168-8 PMC9482625 36115852

[34] Hao Y, Chun A, Cheung K, Rashidi B, Yang X. Tumor Suppressor LATS1 Is a Negative Regulator of Oncogene YAP. J Biol Chem. 2008;283:5496–509. 10.1074/jbc.M709037200 18158288

[35] Chen L, Chan SW, Zhang X, Walsh M, Lim CJ, Hong W, et al. Structural basis of YAP recognition by TEAD4 in the Hippo pathway. Genes Dev. 2010;24:290–300. 10.1101/gad.1865310 PMC2811830 20123908

[36] Wang H, Jayawardana IM, Fleisch JM, Frank D, Zhao B, Kamaras C, et al. Regulation of YAP activity by nuclear G-actin binding. Nucleic Acids Res. 2026;54:gkag248. 10.1093/nar/gkag248 PMC13000468 41854080

[37] Yang H, Xue M, Su P, Zhou Y, Li X, Li Z, et al. RNF31 represses cell progression and immune evasion via YAP/PD-L1 suppression in triple negative breast Cancer. J Exp Clin Cancer Res: CR. 2022;41:364. 10.1186/s13046-022-02576-y PMC9801641 36581998

[38] Kim MH, Kim CG, Kim S-K, Shin SJ, Choe EA, Park S-H, et al. YAP-Induced PD-L1 Expression Drives Immune Evasion in BRAFi-Resistant Melanoma. Cancer Immunol Res. 2018;6:255–66. 10.1158/2326-6066.CIR-17-0320 29382670

[39] Hsu P-C, Miao J, Wang Y-C, Zhang W-Q, Yang Y-L, Wang C-W, et al. Inhibition of yes-associated protein down-regulates PD-L1 (CD274) expression in human malignant pleural mesothelioma. J Cell Mol Med. 2018;22:3139. 10.1111/jcmm.13593 PMC5980156 29575535

[40] Chao Y-C, Lee K-Y, Wu S-M, Kuo D-Y, Shueng P-W, Lin C-W. Melatonin Downregulates PD-L1 Expression and Modulates Tumor Immunity in KRAS-Mutant Non-Small Cell Lung Cancer. Int J Mol Sci. 2021;22:5649. 10.3390/ijms22115649 PMC8199131 34073318

[41] Wang S, Su D, Chen H, Lai J-C, Tang C, Li Y, et al. PD-L2 drives resistance to EGFR-TKIs: dynamic changes of the tumor immune environment and targeted therapy. Cell Death Differ. 2024;31:1140–56. 10.1038/s41418-024-01317-2 PMC11369230 38816578

[42] Postow MA, Callahan MK, Wolchok JD. Immune Checkpoint Blockade in Cancer Therapy. J Clin Oncol. 2015;33:1974. 10.1200/JCO.2014.59.4358 PMC4980573 25605845

[43] Hargadon KM, Johnson CE, Williams CJ. Immune checkpoint blockade therapy for cancer: An overview of FDA-approved immune checkpoint inhibitors. Int Immunopharmacol. 2018;62:29–39. 10.1016/j.intimp.2018.06.001 29990692

[44] Kubli SP, Berger T, Araujo DV, Siu LL, Mak TW. Beyond immune checkpoint blockade: emerging immunological strategies. Nat Rev Drug Discov. 2021;20:899–919. 10.1038/s41573-021-00155-y 33686237

[45] Huang J, Wang Y, Zhang H, Hu X, Wang P, Cai W, et al. Clinical outcomes of second-line treatment following first-line VEGFR-TKI failure in patients with metastatic renal cell carcinoma: a comparison of axitinib alone and axitinib plus anti-PD-1 antibody. Cancer Commun. 2021;41:1071–4. 10.1002/cac2.12206 PMC8504141 34363742

[46] Rini BI, Plimack ER, Stus V, Gafanov R, Hawkins R, Nosov D, et al. Pembrolizumab plus Axitinib versus Sunitinib for Advanced Renal-Cell Carcinoma. N Engl J Med. 2019;380:1116–27. 10.1056/NEJMoa1816714 30779529

[47] Fu Y, Zhang Y, Lei Z, Liu T, Cai T, Wang A, et al. Abnormally activated OPN/integrin αVβ3/FAK signalling is responsible for EGFR-TKI resistance in EGFR mutant non-small-cell lung cancer. J Hematol Oncol. 2020;13:169. 10.1186/s13045-020-01009-7 PMC7720454 33287873

[48] Chen Y, Lu Z, Qi C, Yu C, Li Y, Huan W, et al. N6-methyladenosine-modified TRAF1 promotes sunitinib resistance by regulating apoptosis and angiogenesis in a METTL14-dependent manner in renal cell carcinoma. Molecular Cancer; 21. Epub ahead of print 2022. 10.1186/s12943-022-01549-1. PMC9087993 35538475

[49] Pan Y, Lu X, Shu G, Cen J, Lu J, Zhou M, et al. Extracellular Vesicle-Mediated Transfer of LncRNA IGFL2-AS1 Confers Sunitinib Resistance in Renal Cell Carcinoma. Cancer Res. 2023;83:103–16. 10.1158/0008-5472.CAN-21-3432 PMC9811158 36264173

[50] Liberti MV, Locasale JW. The Warburg Effect: How Does it Benefit Cancer Cells? Trends Biochemical Sci. 2016;41:211–8. 10.1016/j.tibs.2015.12.001 PMC4783224 26778478

[51] Wan N, Wang N, Yu S, Zhang H, Tang S, Wang D, et al. Cyclic immonium ion of lactyllysine reveals widespread lactylation in the human proteome. Nat Methods. 2022;19:854–64. 10.1038/s41592-022-01523-1 35761067

[52] Yang Z, Yan C, Ma J, Peng P, Ren X, Cai S, et al. Lactylome analysis suggests lactylation-dependent mechanisms of metabolic adaptation in hepatocellular carcinoma. Nat Metab. 2023;5:61–79. 10.1038/s42255-022-00710-w 36593272

[53] Li W, Zhou C, Yu L, Hou Z, Liu H, Kong L, et al. Tumor-derived lactate promotes resistance to bevacizumab treatment by facilitating autophagy enhancer protein RUBCNL expression through histone H3 lysine 18 lactylation (H3K18la) in colorectal cancer. Autophagy. 2023;20:114. 10.1080/15548627.2023.2249762 PMC10761097 37615625

[54] Kung H-N, Marks JR, Chi J-T. Glutamine Synthetase Is a Genetic Determinant of Cell Type–Specific Glutamine Independence in Breast Epithelia. PLoS Genet. 2011;7:e1002229. 10.1371/journal.pgen.1002229 PMC3154963 21852960

[55] Goodwin J, Neugent ML, Lee SY, Choe JH, Choi H, Jenkins DMR, et al. The distinct metabolic phenotype of lung squamous cell carcinoma defines selective vulnerability to glycolytic inhibition. Nat Commun. 2017;8:15503. 10.1038/ncomms15503 PMC5458561 28548087

[56] Hensley CT, Faubert B, Yuan Q, Lev-Cohain N, Jin E, Kim J, et al. Metabolic heterogeneity in human lung tumors. Cell. 2016;164:681. 10.1016/j.cell.2015.12.034 PMC4752889 26853473

[57] Zhang L, Sun Y, Lin Y, Li H, Huang Y, Tang N, et al. Cell calcification reverses the chemoresistance of cancer cells via the conversion of glycolipid metabolism. Biomaterials. 2025;314:122886. 10.1016/j.biomaterials.2024.122886 39427430

[58] Zhu R, Ye X, Lu X, Xiao L, Yuan M, Zhao H, et al. ACSS2 acts as a lactyl-CoA synthetase and couples KAT2A to function as a lactyltransferase for histone lactylation and tumor immune evasion. Cell Metab. 2025;37:361–76. 10.1016/j.cmet.2024.10.015 39561764

[59] Feng C, Li Y, Li K, Lyu Y, Zhu W, Jiang H, et al. PFKFB4 is overexpressed in clear-cell renal cell carcinoma promoting pentose phosphate pathway that mediates Sunitinib resistance. J Exp Clin Cancer Res. 2021;40:308. 10.1186/s13046-021-02103-5 PMC8482632 34593007

[60] Fang L, Teng H, Wang Y, Liao G, Weng L, Li Y, et al. SET1A-Mediated Mono-Methylation at K342 Regulates YAP Activation by Blocking Its Nuclear Export and Promotes Tumorigenesis. Cancer Cell. 2018;34:103–18.e9. 10.1016/j.ccell.2018.06.002 30008322

[61] Lv M, Gong Y, Liu X, Wang Y, Wu Q, Chen J, et al. CDK7-YAP-LDHD axis promotes D-lactate elimination and ferroptosis defense to support cancer stem cell-like properties. Signal Transduct Target Ther. 2023;8:302. 10.1038/s41392-023-01555-9 PMC10427695 37582812

[62] Wang S, Xie F, Chu F, Zhang Z, Yang B, Dai T, et al. YAP antagonizes innate antiviral immunity and is targeted for lysosomal degradation through IKKɛ-mediated phosphorylation. Nat Immunol. 2017;18:733–43. 10.1038/ni.3744 28481329

[63] Peng C, Zhu Y, Zhang W, Liao Q, Chen Y, Zhao X, et al. Regulation of the Hippo-YAP Pathway by Glucose Sensor O-GlcNAcylation. Mol Cell. 2017;68:591–604.e5. 10.1016/j.molcel.2017.10.010 29100056

[64] Oudhoff MJ, Freeman SA, Couzens AL, Antignano F, Kuznetsova E, Min PH, et al. Control of the Hippo Pathway by Set7-Dependent Methylation of Yap. Developmental Cell. 2013;26:188–94. 10.1016/j.devcel.2013.05.025 23850191

[65] Ju J, Zhang H, Lin M, Yan Z, An L, Cao Z, et al. The alanyl-tRNA synthetase AARS1 moonlights as a lactyl-transferase to promote YAP signaling in gastric cancer. J Clin Investig. Epub ahead of print 21 March 2024. 10.1172/JCI174587. PMC11093599 38512451

[66] Gu W, Roeder RG. Activation of p53 Sequence-Specific DNA Binding by Acetylation of the p53 C-Terminal Domain. Cell. 1997;90:595–606. 10.1016/s0092-8674(00)80521-8 9288740

[67] Kim E-J, Kho J-H, Kang M-R, Um S-J. Active Regulator of SIRT1 Cooperates with SIRT1 and Facilitates Suppression of p53 Activity. Mol Cell. 2007;28:277–90. 10.1016/j.molcel.2007.08.030 17964266

[68] Wu Z, Su J, Li F, Chen T, Mayner J, Engler A, et al. YAP silencing by RB1 mutation is essential for small-cell lung cancer metastasis. Nat Commun. 2023;14:5916. 10.1038/s41467-023-41585-z PMC10516997 37739954

[69] Dunaway LS, Pollock JS. HDAC1: an environmental sensor regulating endothelial function. Cardiovascular Res. 2021;118:1885. 10.1093/cvr/cvab198 PMC9239577 34264338

[70] Jiang M, Qi F, Zhang K, Zhang X, Ma J, Xia S, et al. MARCKSL1–2 reverses docetaxel-resistance of lung adenocarcinoma cells by recruiting SUZ12 to suppress HDAC1 and elevate miR-200b. Mol Cancer. 2022;21:150. 10.1186/s12943-022-01605-w PMC9306054 35864549

[71] New M, Olzscha H, La Thangue NB. HDAC inhibitor-based therapies: Can we interpret the code? Mol Oncol. 2012;6:637–56. 10.1016/j.molonc.2012.09.003 PMC5528347 23141799

[72] Shah RR. Safety and Tolerability of Histone Deacetylase (HDAC) Inhibitors in Oncology. Drug Saf. 2019;42:235–45. 10.1007/s40264-018-0773-9 30649740

[73] West AC, Johnstone RW. New and emerging HDAC inhibitors for cancer treatment. J Clin Invest. 2014;124:30–39. 10.1172/JCI69738 PMC3871231 24382387

[74] Witta SE, Jotte RM, Konduri K, Neubauer MA, Spira AI, Ruxer RL, et al. Randomized Phase II Trial of Erlotinib With and Without Entinostat in Patients With Advanced Non–Small-Cell Lung Cancer Who Progressed on Prior Chemotherapy. J Clin Oncol. 2012;30:2248–55. 10.1200/JCO.2011.38.9411 PMC4798782 22508830

[75] Nakagawa T, Takeuchi S, Yamada T, Ebi H, Sano T, Nanjo S, et al. EGFR-TKI Resistance Due to BIM Polymorphism Can Be Circumvented in Combination with HDAC Inhibition. Cancer Res. 2013;73:2428–34. 10.1158/0008-5472.CAN-12-3479 23382048

[76] Rose TL, Kim WY. Renal Cell Carcinoma: A Review. JAMA. 2024;332:1001. 10.1001/jama.2024.12848 PMC11790279 39196544

[77] Suwaki N, Vanhecke E, Atkins KM, Graf M, Swabey K, Huang P, et al. A HIF-regulated VHL-PTP1B-Src signaling axis identifies a therapeutic target in Renal Cell Carcinoma. Sci Transl Med. 2011;3:85ra47. 10.1126/scitranslmed.3002004 PMC3303496 21632985

[78] Biswas S, Eisen T. Immunotherapeutic strategies in kidney cancer—when TKIs are not enough. Nat Rev Clin Oncol. 2009;6:478–87. 10.1038/nrclinonc.2009.91 19546865

[79] Yi M, Jiao D, Qin S, Chu Q, Wu K, Li A. Synergistic effect of immune checkpoint blockade and anti-angiogenesis in cancer treatment. Mol Cancer. 2019;18:60. 10.1186/s12943-019-0974-6 PMC6441150 30925919

[80] Yu T, Liu Z, Tao Q, Xu X, Li X, Li Y, et al. Targeting tumor-intrinsic SLC16A3 to enhance anti-PD-1 efficacy via tumor immune microenvironment reprogramming. Cancer Lett. 2024;589:216824. 10.1016/j.canlet.2024.216824 38522774

[81] Peng S, Wang R, Zhang X, Ma Y, Zhong L, Li K, et al. EGFR-TKI resistance promotes immune escape in lung cancer via increased PD-L1 expression. Mol Cancer. 2019;18:165. 10.1186/s12943-019-1073-4 PMC6864970 31747941

