# Supplementary material for: YAP delactylation enhances therapy resistance in renal cell carcinoma
Source: Cell Death Dis. 2026 Aug 15;17(1):961. doi: 10.1038/s41419-026-09204-z (PMC13755448; doi:10.1038/s41419-026-09204-z)
Supplement: Supplementary file 1 [file 41419_2026_9204_MOESM1_ESM.pdf]

Supplementary material

YAP delactylation enhances therapy resistance in renal cell carcinoma

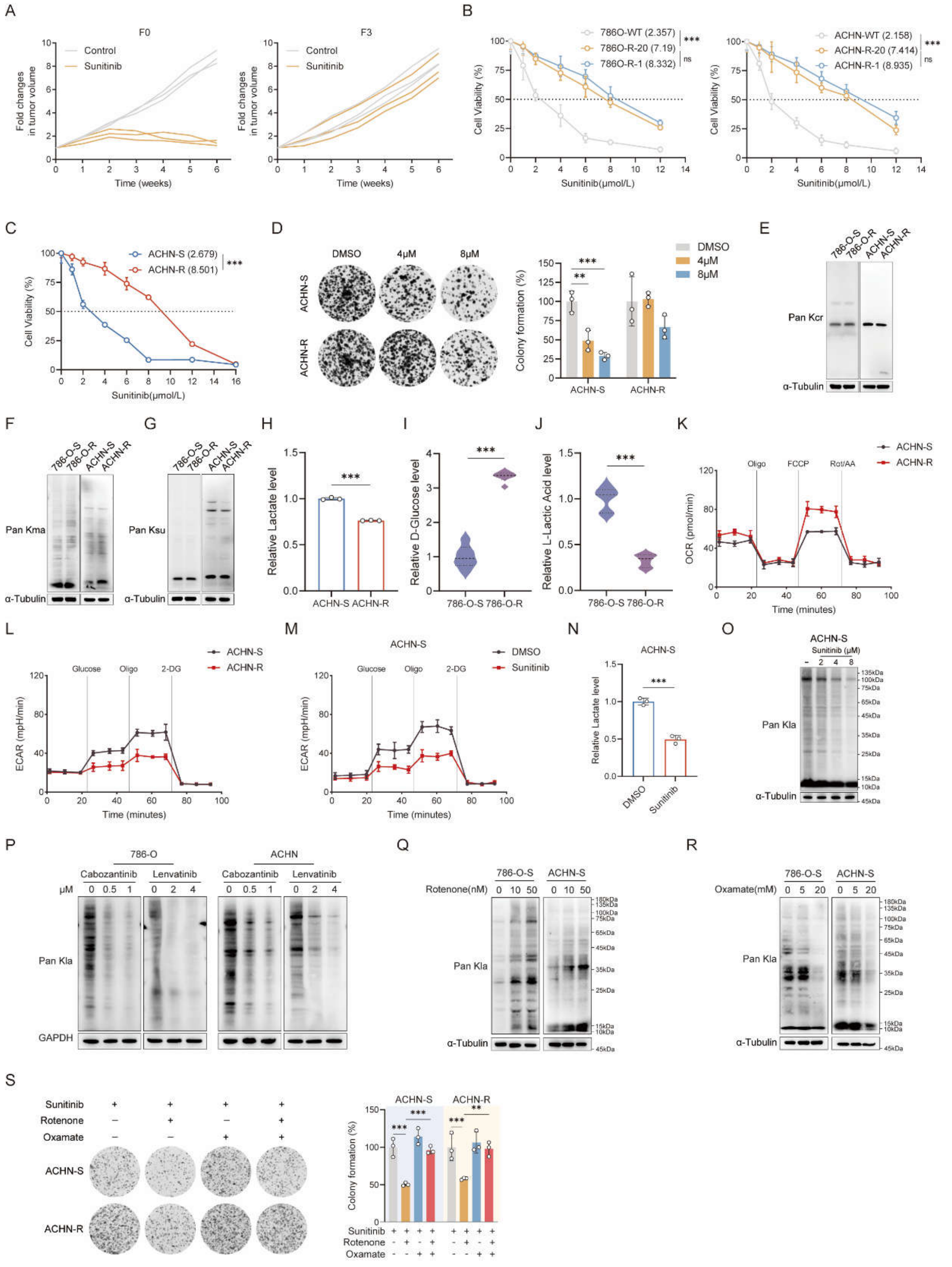

Figure S1. Lactylation inhibits TKI targeted treatment resistance in renal cancer.

(A) Subcutaneous tumor growth of first (left) and third (right) generation renal cancer cells in NCG mice.

(B) Relative cell viability of 786-O-R (left) and ACHN-R (right) drug-free cultures for one and 20 generations versus wild-type cells. The values in parentheses represent the IC50.

(C) Relative cell viability of ACHN calculated by CCK8. The values in parentheses represent the IC50.

(D) Left: colony formation assay treating ACHN cells (1,000 cells per well in a 6-well plate) with 4 μmol/L sunitinib for 7 days; Right: Quantification of the data.

(E–G) Immunoblot analysis of pan-crotonyllysine (E), pan-malonyllysine (F) and pan-succinyllysine (G) levels in 786-O and ACHN cells.

(H) Lactate level measurement in ACHN cells.

(I, J) Metabolomics analysis reveals the D-Glucose (I) and L-Lactic acid (J) levels in 786-O cells.

(K, L) OCR (K) and ECAR (L) measurement in ACHN cells.

(M, N) ECAR (M) and lactate level (N) were measured in ACHN-S cells treated with 4 μmol/L sunitinib or DMSO.

(O) The pan-lactylation level was measured by Immunoblot analysis in ACHN-S cells treated with sunitinib or DMSO.

(P) Immunoblot analysis of pan-lactylation levels in 786-O and ACHN cells treated with cabozantinib or lenvatinib.

(Q, R) Immunoblot analysis of 786-O-S and ACHN-S cells treated with rotenone (Q) and oxamate (R), respectively.

(S) ACHN cells (1,000 cells per well in a 6-well plate) were treated with 4 μmol/L sunitinib, 20 mmol/L oxamate, and 50 nmol/L rotenone, respectively, for colony formation assays (left) and data quantification (right).

The results shown are representative images from three independent experiments (n=3). The statistical data in B is analyzed using one-way ANOVA followed by multiple comparisons test. The statistical data in C, H–J, and N are analyzed using unpaired two-tailed Student's t-test. The statistical data in D and S are analyzed using two-way ANOVA followed by multiple comparisons test. ns, not significant; $^{**}p < 0.01$, $^{***}p < 0.001$.

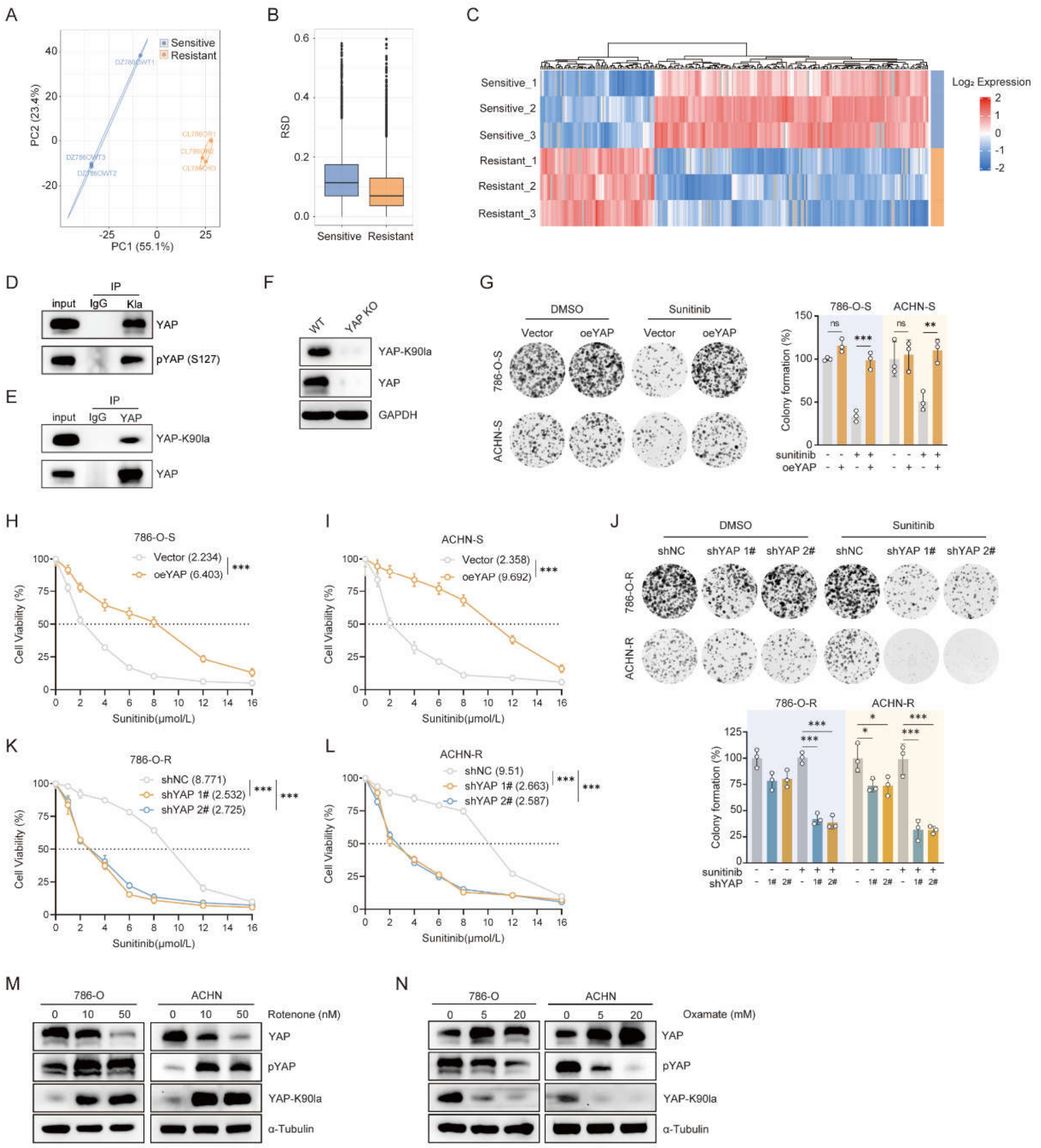

Figure S2. Lactate induces YAP-K90 lactylation

(A) PCA analysis of lactylation proteomics.

(B) Relative standard deviation (RSD) of replicate samples from each group of lactylation proteomics plotted as a box line graph.

(C) Heatmap of DEKSs.

(D, E) Immunoblot analysis of whole cell lysates (input) and immunoprecipitates (IP) from 786-O. The samples were probed with anti-pan-Kla (D) and anti-YAP antibodies (E), with rabbit IgG serving as the negative control for immunoprecipitation.

(F) Immunoblot analysis of YAP-KO in 786-O cells.

(G) Colony formation assay (left) and data quantification (right) of 786-O-S and ACHN-S cells

(1,000 cells per well in 6-well plates) oeYAP treated with 4 μmol/L sunitinib for 7 days.

(H, I) Relative viability assay of 786-O-S (H) and ACHN-S (I) cells overexpressing YAP. The values in parentheses represent the IC50.

(J) Colony formation assay (top) and data quantification (bottom) of 786-O-R and ACHN-R cells (1,000 cells per well in 6-well plates) shYAP treated with 4 μmol/L sunitinib for 7 days.

(K, L) Relative viability assay of 786-O-R (K) and ACHN-R (L) cells shYAP. The values in parentheses represent the IC50.

(M, N) Immunoblot analysis of 786-O and ACHN cells treated with Rotenone (M) or Oxamate (N) for 24h, respectively.

The results shown are representative images from three independent experiments (n=3). The statistical data in G and J are analyzed using two-way ANOVA followed by multiple comparisons test. The statistical data in H and I are analyzed using unpaired two-tailed Student's t-test. The statistical data in K and L are analyzed using one-way ANOVA followed by multiple comparisons test. ns, not significant; *$p < 0.05$, **$p < 0.01$, ***$p < 0.001$.

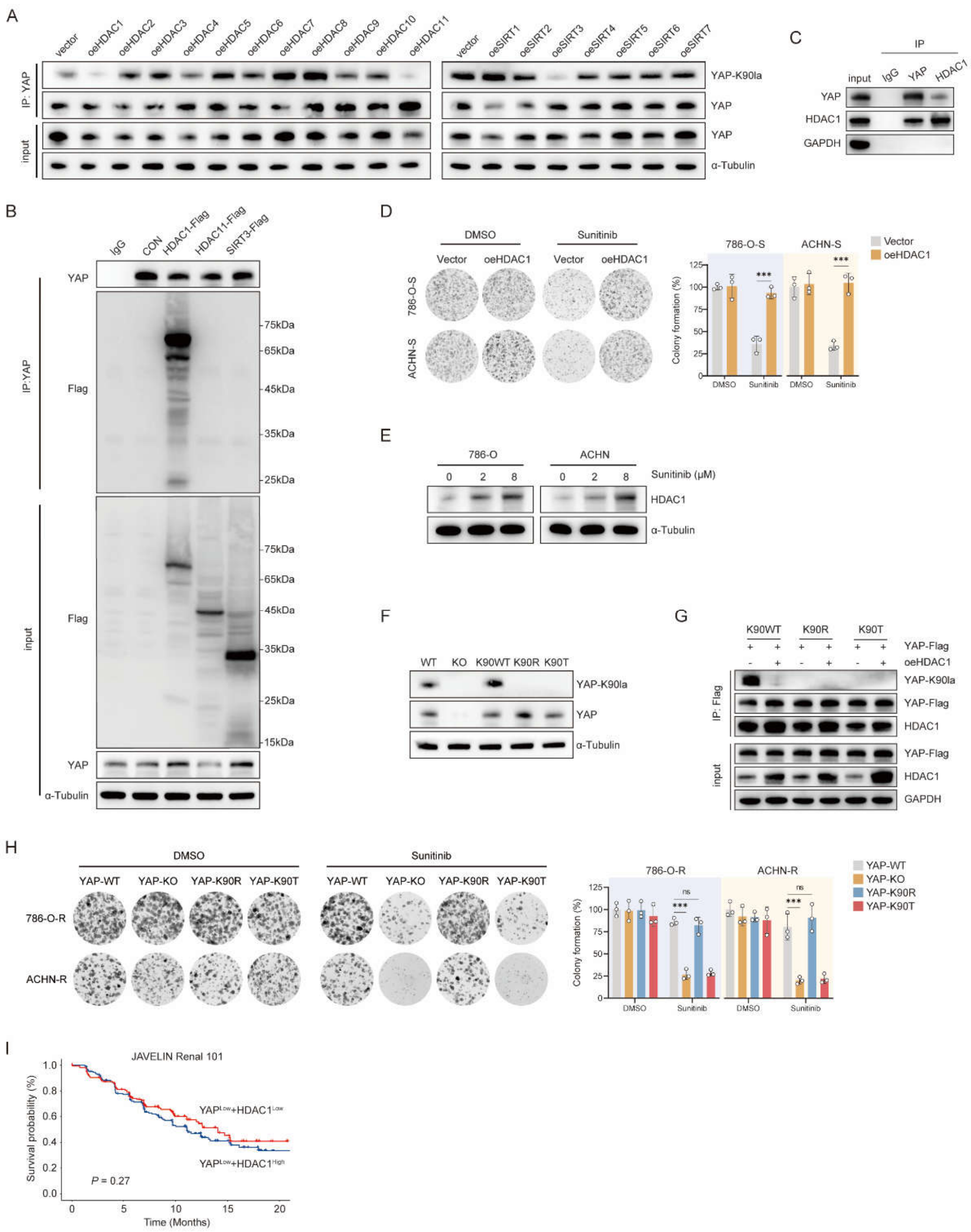

Figure S3. YAP K90 is delactylated by HDAC1

(A) A series of HDAC- or SIRT-expressing plasmids were transfected into 786-O cells, and the cells were then subjected to immunoprecipitation using an anti-YAP antibody to determine the level of YAP-K90la by immunoblotting analysis.

(B) Immunoblot analysis of YAP interaction with HDAC1, HDAC11 and SIRT3 in 786-O cells.

(C) Immunoblot analysis of YAP interaction with HDAC1 in 786-O cells.

(D) Colony formation assay (left) and data quantification (right) of 786-O-S and ACHN-S cells (1,000 cells per well in 6-well plates) overexpressing HDAC1 treated with 4 μmol/L sunitinib for 7

days.

(E) Immunoblot analysis of HDAC1 levels in 786-O and ACHN cells following sunitinib treatment.

(F) Immunoblot analysis of YAP-K90la and YAP levels in YAP-WT, KO, K90WT, K90R and K90T cells.

(G) Immunoblot analysis of YAP-K90la in anti-flag immunoprecipitates of YAP-WT, K90R, and K90T.

(H) Colony formation assay (left) and data quantification (right) of 786-O-R and ACHN-R cells (1000 cells/well in 6-well plates) treated with 4 μmol/L sunitinib for 7 days, including YAP-WT, YAP-KO, and cells transfected with YAP-K90R or YAP-K90T.

(I) Kaplan-Meier analysis of Progression-free survival in patients from the JAVELIN Renal 101 Phase 3 Trial, stratified by tumor YAP and HDAC1 level.

The results shown are representative images from three independent experiments (n=3). The statistical data in D and H are analyzed using two-way ANOVA followed by multiple comparisons test. ns, not significant; ***$p < 0.001$.

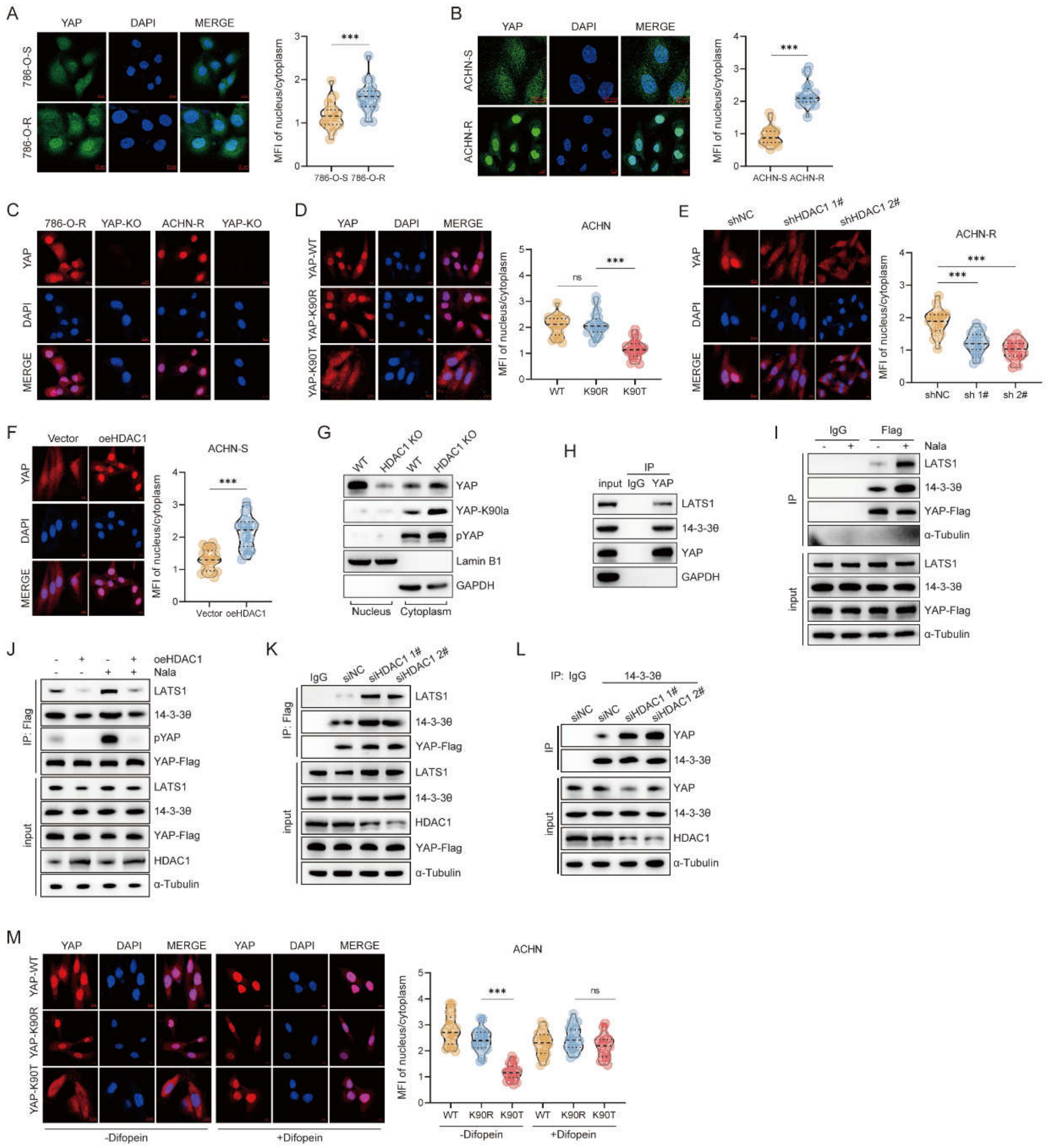

Figure S4. Delactylation of YAP disrupts its interaction with LATS1, prevents its phosphorylation, and promotes its nuclear localization.

(A) Immunofluorescence analysis (left) and quantitative data (right) of YAP and in 786-O-S and 786-O-R cells.

(B) Immunofluorescence analysis (left) and quantitative data (right) of YAP and in ACHN-S and ACHN-R cells.

(C) Immunofluorescence analysis of YAP in YAP KO cells.

(D) Immunofluorescence analysis (left) and quantitative data (right) of YAP WT, YAP-K90R and YAP-K90T cell localization in ACHN cells.

(E) Immunofluorescence analysis (left) and data quantification (right) of changes in YAP cellular localization in HDAC1-knockdown ACHN-R cells.

(F) Immunofluorescence analysis (left) and data quantification (right) of changes in YAP cellular localization in HDAC1-overexpressing ACHN-S cells.

(G) Nucleocytoplasmic separation and immunoblotting analysis of changes in YAP cellular localization in 786-O cells of HDAC1 KO.

(H) Immunoprecipitation analysis of YAP interaction with LATS1 and 14-3-3θ in 786-O cells.

(I) Immunoprecipitation analysis of YAP interaction with LATS1 and 14-3-3θ in 786-O cells after 10 mmol/L NaLa treatment.

(J) Immunoblot analysis of LATS1, 14-3-3θ and YAP interactions in 786-O cells treated with 10 mmol/L Nala in WT or overexpressing HDAC1.

(K, L) Immunoblot analysis of the interaction between YAP and LATS1, 14-3-3θ in 786-O cells following anti-Flag (K) and anti-14-3-3θ (L) immunoprecipitation.

(M) Immunofluorescence analysis (left) and data quantification (right) of difopein-treated ACHN cells with anti-YAP antibody and detection of changes in cellular localization of YAP-WT, K90R and K90T.

The results shown are representative images from three independent experiments (n=3). Nuclear localization was quantified using MFI, and all images were acquired as single-plane images. The statistical data in A, B, and F are analyzed using unpaired two-tailed Student's t-test. The statistical data in D, E, and M are analyzed using one-way ANOVA followed by multiple comparisons test. ns, not significant; ***$p < 0.001$. MFI, mean fluorescence intensity.

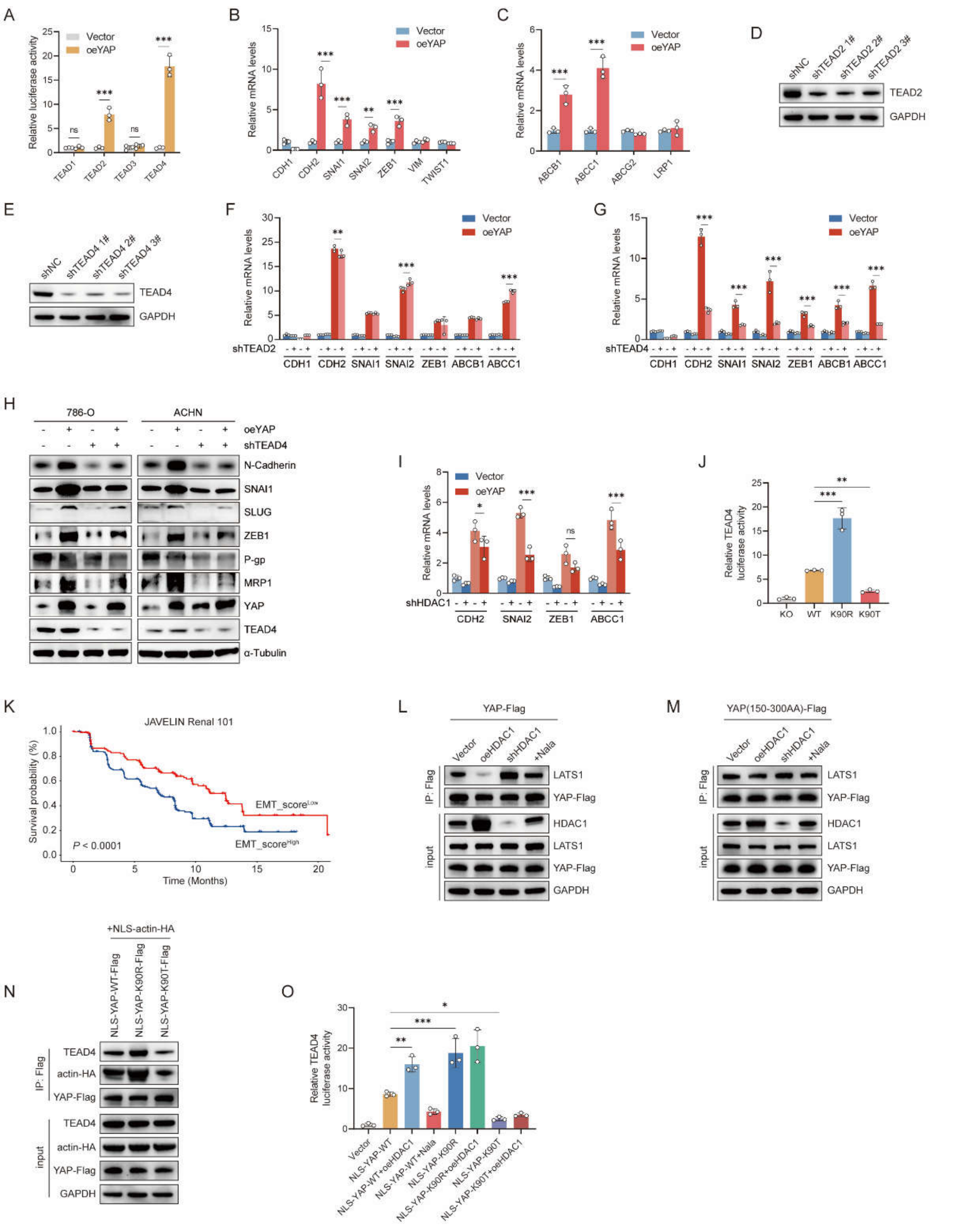

Figure S5. Delactylation of YAP promotes transcriptional activation of TEAD.

(A) Relative luciferase activity analysis of TEAD in 786-O cells.

(B, C) mRNA expression levels of YAP target genes in 786-O cells.

(D, E) Immunoblot analysis validating the knockdown efficiency of three independent shRNAs targeting TEAD2 (D) and TEAD4 (E) in 786-O cells.

(F, G) mRNA expression levels of YAP target genes in 786-O cells after shTEAD2 (F) and shTEAD4

(G) treatment.

(H) Immunoblot analysis of shTEAD4 and cotransfected HDAC1 verified YAP target proteins.

(I) mRNA expression levels levels of YAP target genes in 786-O cells after shHDAC1 treatment.

(J) Relative TEAD4-dependent luciferase reporter activity in 786-O cells with YAP with different mutations.

(K) Kaplan-Meier analysis of Progression-free survival in patients from the JAVELIN Renal 101 Phase 3 Trial, stratified by EMT_score.

(L) Immunoblot analysis of the interaction between full-length YAP and LATS1 in 786-O cells following HDAC1 overexpression, shHDAC1, or NaLa treatment.

(M) Immunoblot analysis of the interaction between the YAP(150–300 AA) fragment and LATS1 in 786-O cells following HDAC1 overexpression, HDAC1 knockdown, or NaLa treatment.

(N) Immunoblot analysis of the interaction between YAP, nuclear actin, and TEAD4 in 786-O cells co-expressing NLS-actin-HA with NLS-YAP-WT, NLS-YAP-K90R, or NLS-YAP-K90T.

(O) Relative TEAD4-dependent luciferase reporter activity in 786-O cells expressing NLS-YAP-WT, NLS-YAP-K90R, or NLS-YAP-K90T with or without HDAC1 overexpression.

The results shown are representative images from three independent experiments (n=3). The statistical data in A–C, F, G, and I are analyzed using two-way ANOVA followed by multiple comparisons test. The statistical data in J and O are analyzed using one-way ANOVA followed by multiple comparisons test. ns, not significant; *$p < 0.05$, **$p < 0.01$, ***$p < 0.001$.

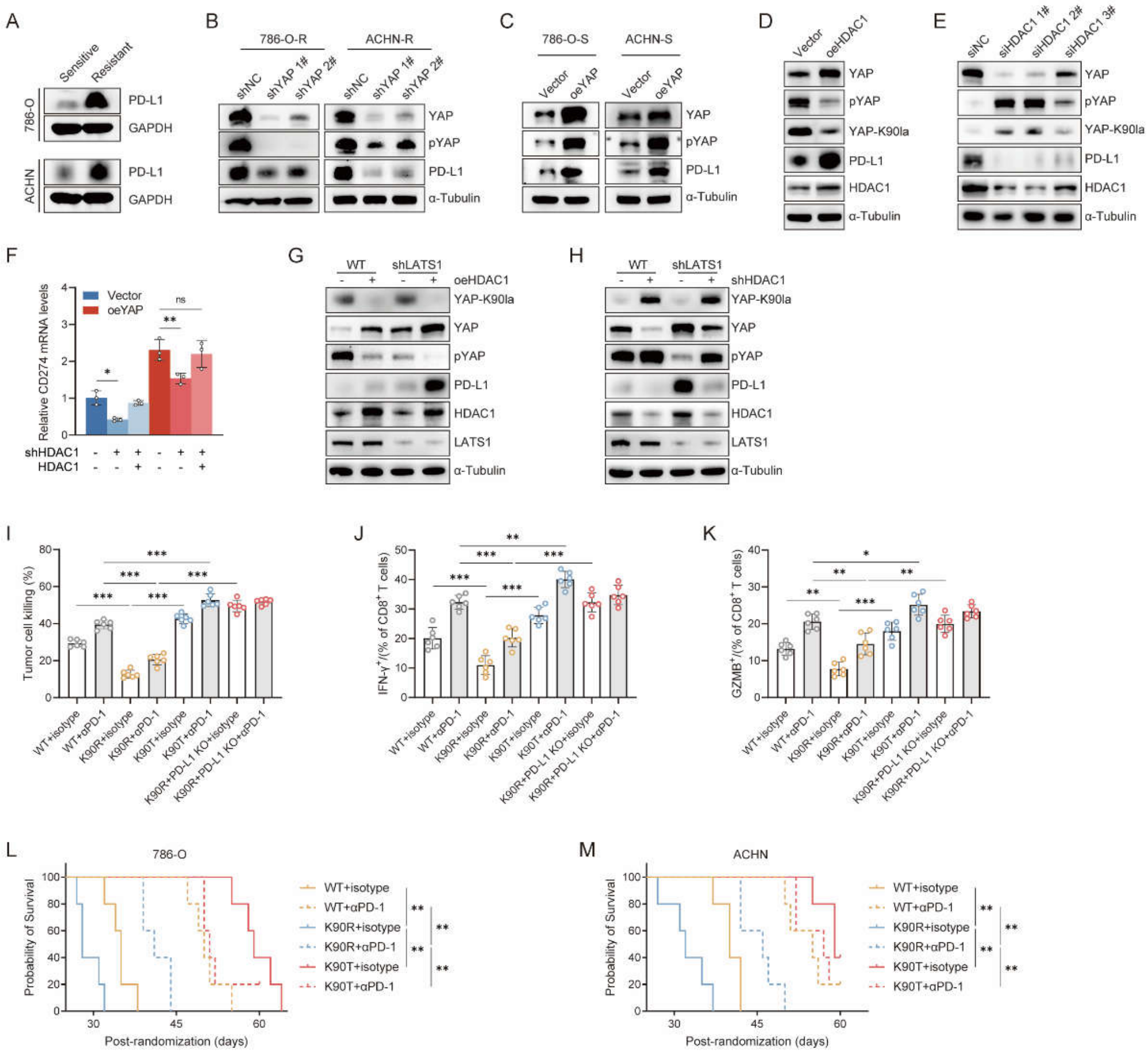

Figure S6. YAP-K90 delactylation promotes PD-L1-mediated immunosuppression.

(A) Immunoblot analysis of relative expression levels of PD-L1 in sensitive and resistant cells.

(B) Immunoblot analysis of relative expression levels of PD-L1, YAP and pYAP in drug-resistant cells of shYAP.

(C) Immunoblot analysis of relative expression levels of PD-L1, YAP and pYAP in 786-O-S and ACHN-S cells oeYAP.

(D) Immunoblot analysis of PD-L1, YAP, pYAP, and YAP-K90la expression levels in 786-O cells following HDAC1 overexpression.

(E) Immunoblot analysis of PD-L1, YAP, pYAP, and YAP-K90la expression levels in 786-O cells following HDAC1 knockdown.

(F) Re-expression of HDAC1 rescues the suppression of CD274 caused by HDAC1 knockdown in 786-O cells.

(G, H) Immunoblot analysis of YAP-K90la, pYAP, YAP, LATS1 and PD-L1 in 786-O cells following HDAC1 overexpression (G) or HDAC1 knockdown (H).

(I) Flow cytometric analysis of tumor killing rate after co-culture of ACHN cells with activated $CD8^+$ T cells.

(J, K) Flow cytometric analysis of the proportion of IFN-γ^+ $CD8^+$ T cells (J) and $GZMB^+$ $CD8^+$ T cells (K) after co-culture with activated ACHN cells.

(L, M) Survival analysis of $huCD34^+$HSC NCG mice transplanted with 786-O (L) or ACHN (M) cell-derived tumors. (n=5)

The statistical data in F and I–K are analyzed using one-way ANOVA followed by multiple comparisons test. The Log-rank test is used for L and M. ns, not significant; *$p < 0.05$, **$p < 0.01$, ***$p < 0.001$.

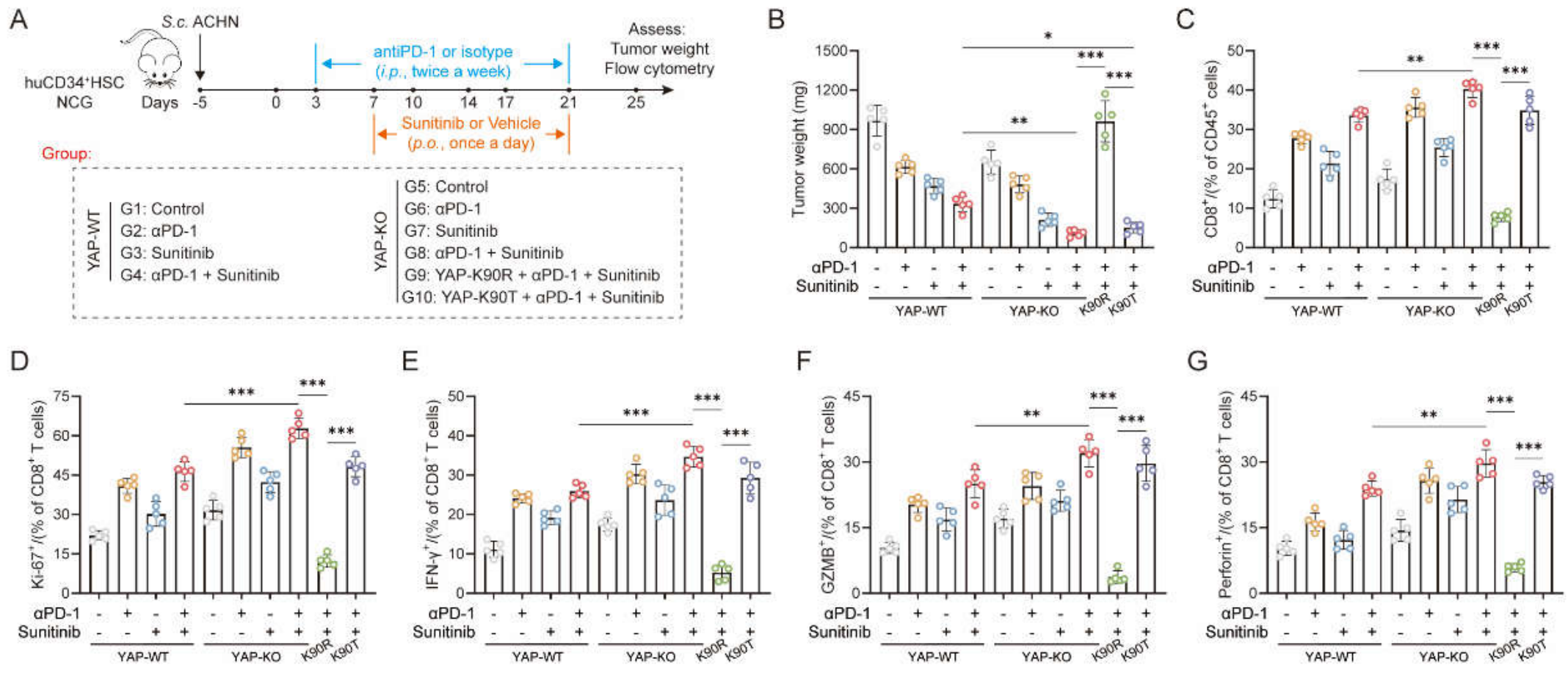

Figure S7. YAP-K90T remodels the TIME and enhance the sunitinib response in RCC

(A) Schematic diagram depicting the treatment of huCD34^{+}HSC NCG mice bearing ACHN-derived tumors with anti-PD-1 antibody or/and sunitinib at the indicated time points.

(B) Measurement of subcutaneous tumor weight in huCD34^{+}HSC NCG mice bearing ACHN cell-derived tumors. (n=5)

(C) Flow cytometric analysis of the percentage of CD8^{+} cells among CD45^{+} cells in subcutaneous tumors derived from the ACHN cell line in huCD34^{+}HSC NCG mice.

(D–G) Flow cytometric analysis of the proportions of Ki-67^{+} (D), IFN-γ^{+} (E), GZMB^{+} (F), and perforin^{+} (G) cells within the CD8^{+} T cell population in ACHN-derived subcutaneous tumors from huCD34^{+}HSC NCG mice.

All statistical analyses were performed using one-way ANOVA followed by multiple-comparisons testing; Data are presented as mean ± SD. ns, not significant; *p < 0.05, **p < 0.01, ***p < 0.001.

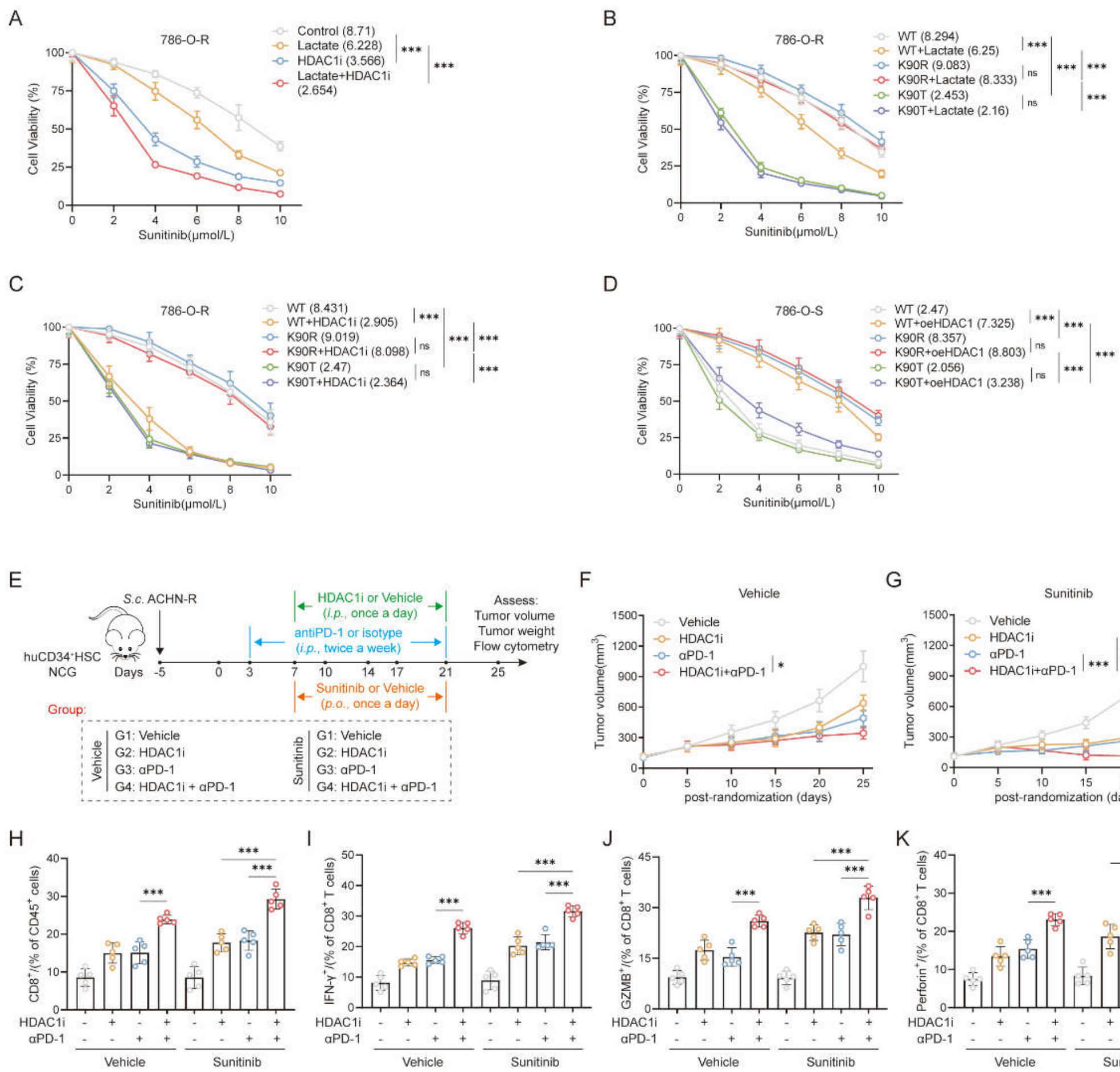

Figure S8. Modulation of YAP-K90 lactylation regulates sunitinib sensitivity in RCC

(A) Cell viability assay of 786-O-R cells treated with lactate (10 mM) or HDAC1 inhibitor. The values in parentheses represent the IC50.

(B) Cell viability assay of 786-O-R cells treated with either lactate (10 mM) or YAP-K90 point mutants. The values in parentheses represent the IC50.

(C) Cell viability assay of 786-O-R cells treated with either HDAC1 inhibitor or YAP-K90 point mutants. The values in parentheses represent the IC50.

(D) Cell viability assay of 786-O-S cells with either HDAC1 overexpression or YAP-K90 point mutation. The values in parentheses represent the IC50.

(E) Schematic diagram of the treatment schedule with anti-PD-1 antibody, sunitinib, and/or HDAC1i at indicated time points in huCD34^{+}HSC NCG mice bearing ACHN-R-derived tumors.

(F, G) Measurement of tumor volume after vehicle (E) or sunitinib (F) treatment in huCD34^{+}HSC

NCG mice bearing ACHN-R subcutaneous tumors. (n=5)

(H) Flow cytometric analysis of the percentage of $CD8^+$ cells among $CD45^+$ cells in subcutaneous tumors derived from the ACHN-R cell line in $huCD34^+$HSC NCG mice.

(I–K) Flow cytometric analysis of the proportions of IFN-γ^+ (I), $GZMB^+$ (J), and $perforin^+$ (K) cells within the $CD8^+$ T cell population in ACHN-R-derived subcutaneous tumors from $huCD34^+$HSC NCG mice.

All statistical analyses were performed using one-way ANOVA followed by multiple-comparisons testing. *$p < 0.05$, ***$p < 0.001$

Table S1. qPCR assay primer sequences, siRNA sequences and shRNA sequences

qPCR primers	Sequence
Human ABCB1 Forward	TTGCTGCTTACATTCAGGTTTCA
Human ABCB1 Reverse	AGCCTATCTCCTGTCGCATTA
Human ABCC1 Forward	CTCTATCTCTCCCGACATGACC
Human ABCC1 Reverse	AGCAGACGATCCACAGCAAAA
Human CDH1 Forward	CGAGAGCTACACGTTCACGG
Human CDH1 Reverse	GGGTGTCGAGGGAAAAATAGG
Human CDH2 Forward	TCAGGCGTCTGTAGAGGCTT
Human CDH2 Reverse	ATGCACATCCTTCGATAAGACTG
Human SNAI1 Forward	TCGGAAGCCTAACTACAGCGA
Human SNAI1 Reverse	AGATGAGCATTGGCAGCGAG
Human SNAI2 Forward	CGAACTGGACACACATACAGTG
Human SNAI2 Reverse	CTGAGGATCTCTGGTTGTGGT
Human ZEB Forward	GATGATGAATGCGAGTCAGATGC
Human ZEB Reverse	ACAGCAGTGTCTTGTTGTTGT
Human CD274 Forward	TGGCATTTGCTGAACGCATTT
Human CD274 Reverse	TGCAGCCAGGTCTAATTGTTTT
Human GAPDH Forward	GGAGCGAGATCCCTCCAAAAT
Human GAPDH Reverse	GGCTGTTGTCATACTTCTCATGG

shRNA	Sequence	Source
shYAP 1#	GCCACCAAGCTAGATAAAGAA	This paper
shYAP 2#	CAGGTGATACTATCAACCAAA	This paper
shYAP 3#	CGACCAATAGCTCAGATCCTT	This paper
shTEAD2 1#	CCCGAAGGAAATCAAGGGAAA	This paper
shTEAD2 2#	CCGGCAGATCTACGACAAATT	This paper
shTEAD4 1#	GAGACAGAGTATGCTCGCTAT	This paper
shTEAD4 2#	CCTTTCTCTCAGCAAACCTAT	This paper
shHDAC1 1#	CCTAATGAGCTTCCATACAAT	This paper
shHDAC1 2#	CGGTTAGGTTGCTTCAATCTA	This paper
shLATS1 1#	CACGGCAAGATAGCATGGATT	This paper
shLATS1 2#	GAAGATAAAGACACTAGGAAT	This paper

siRNA	Target Sequence	Source
siYAP 1#	GAGATACTTCTTAAATCACATCG	This paper
siYAP 2#	CAGGCTAATACAGAAAAAGATGA	This paper
siYAP 3#	AAGCTTTCTTACATGGTTATAGA	This paper
siHDAC1 1#	TACCGAAAAATGGAAATCTATCG	This paper
siHDAC1 2#	TTCTTAACTTTGAACCATAAAGG	This paper
siYWHAQ 1#	ATCGAAAACAAACGATAGATAAT	This paper
siYWHAQ 2#	GGGCTGAAAACTAAATCCATACA	This paper

**Recombinant DNA**
pLVX-puro-Flag-YAP WT
pLVX-puro-Flag-YAP K90R
pLVX-puro-Flag-YAP K90T
pLVX-puro-Flag-YAP WT-NLS
pLVX-puro-Flag-YAP K90R-NLS
pLVX-puro-Flag-YAP K90T-NLS
pLVX-puro-HA-actin-NLS
pLVX-puro-Flag-HDAC1
pLVX-puro-Flag-HDAC2
pLVX-puro-Flag-HDAC3
pLVX-puro-Flag-HDAC4
pLVX-puro-Flag-HDAC5
pLVX-puro-Flag-HDAC6
pLVX-puro-Flag-HDAC7
pLVX-puro-Flag-HDAC8
pLVX-puro-Flag-HDAC9
pLVX-puro-Flag-HDAC10
pLVX-puro-Flag-HDAC11
pLVX-puro-Flag-SIRT1
pLVX-puro-Flag-SIRT2
pLVX-puro-Flag-SIRT3
pLVX-puro-Flag-SIRT4
pLVX-puro-Flag-SIRT5
pLVX-puro-Flag-SIRT6
pLVX-puro-Flag-SIRT7
pLVX-puro-HA-HDAC1 WT
pLVX-puro-HA-HDAC1 H141A
pLVX-puro-HDAC1
pLVX-puro-YWHAQ
