# Supplementary material for: YAP delactylation enhances therapy resistance in renal cell carcinoma
Source: Cell Death Dis. 2026 Aug 15;17(1):961. doi: 10.1038/s41419-026-09204-z (PMC13755448; doi:10.1038/s41419-026-09204-z)

WB images in Figure 1

Figure 1D

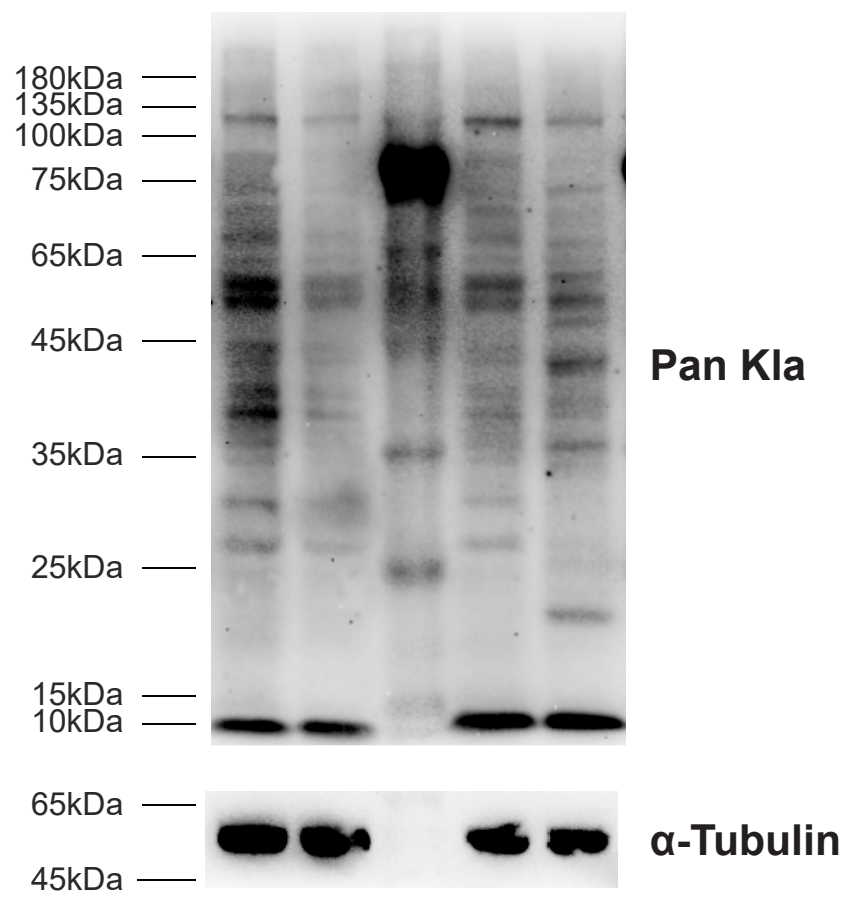

Figure 1K

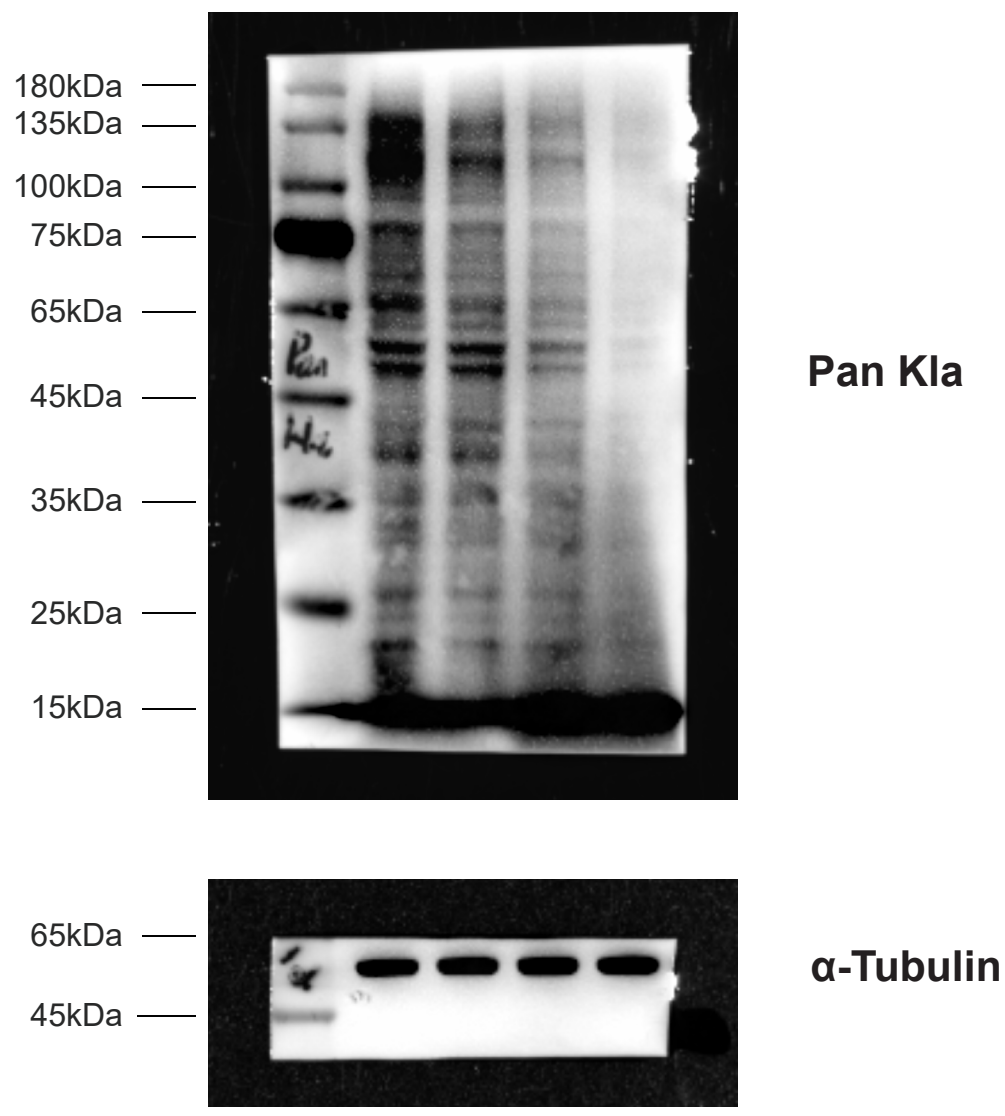

WB images in Figure 2

Figure 2M

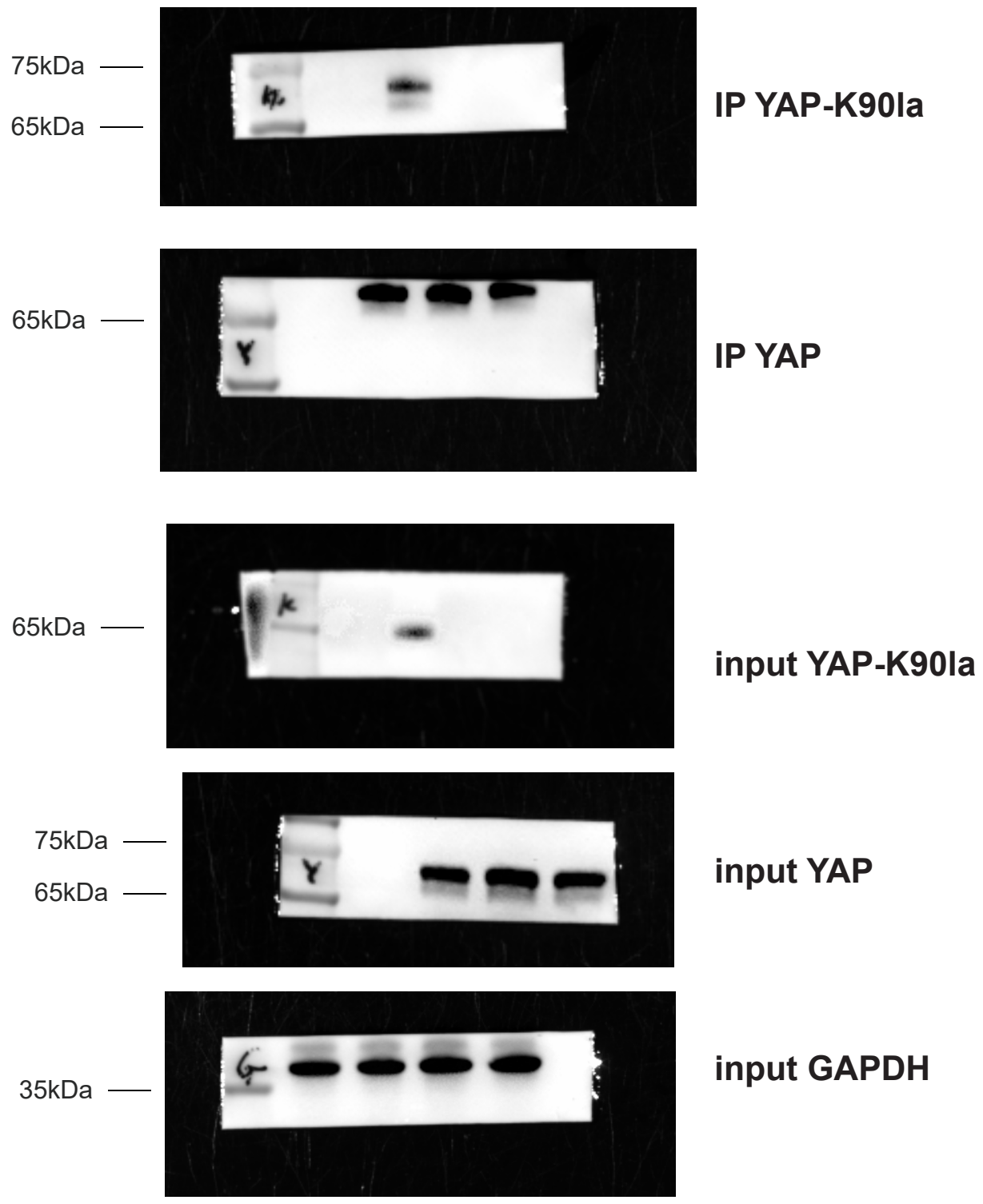

Figure 2N

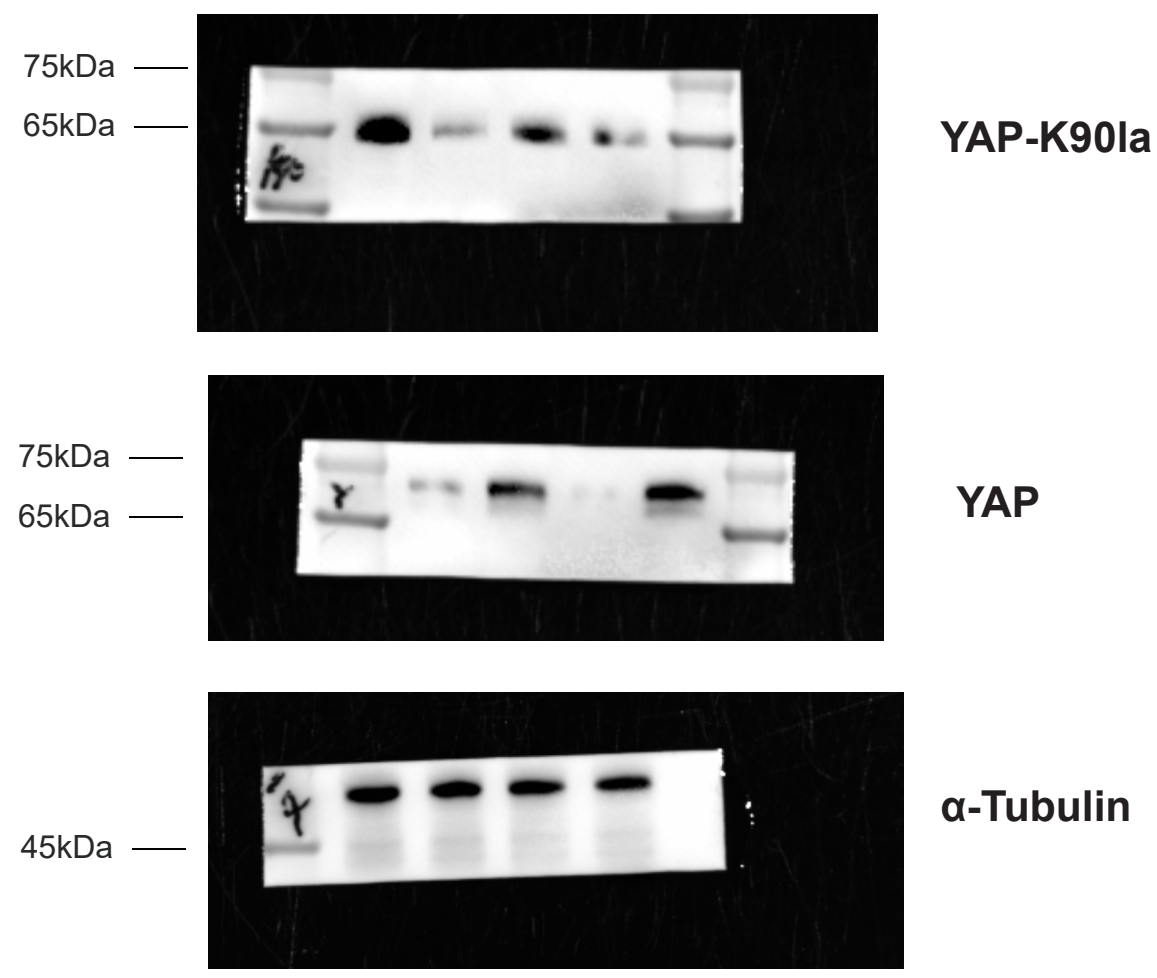

WB images in Figure 3

Figure 3C

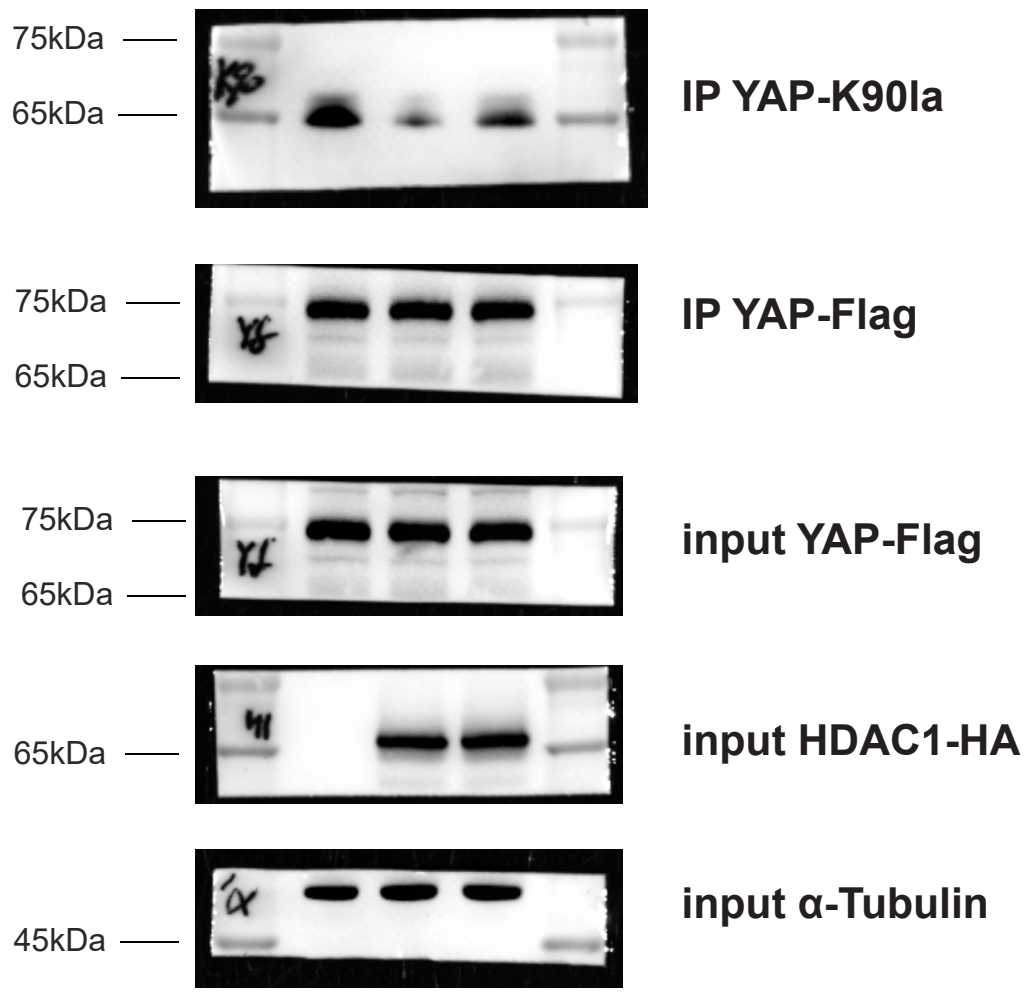

Figure 3D

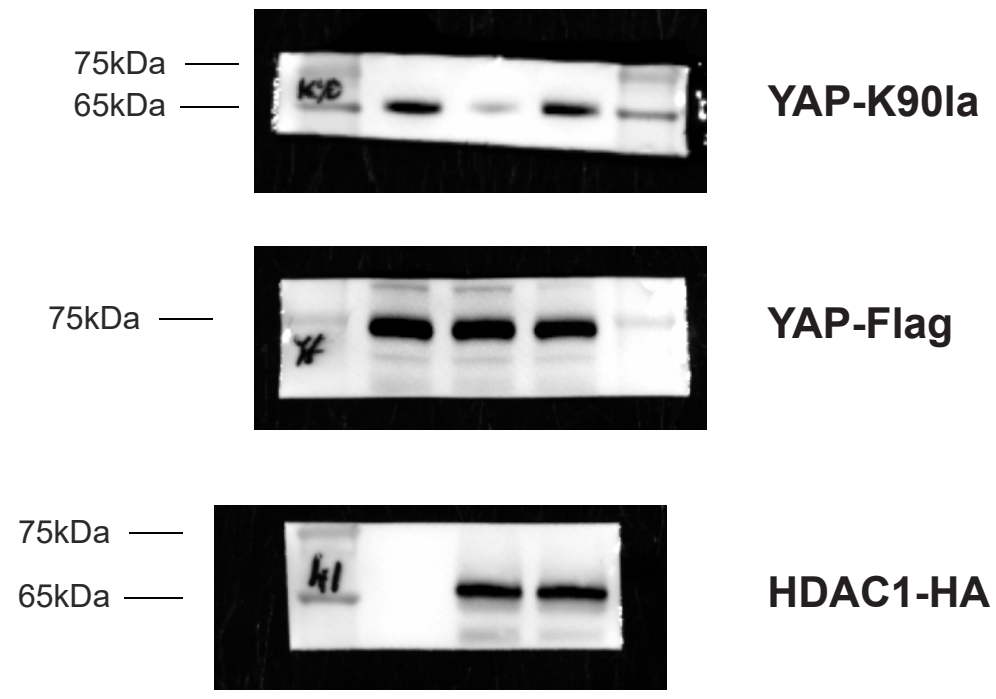

Figure 3E

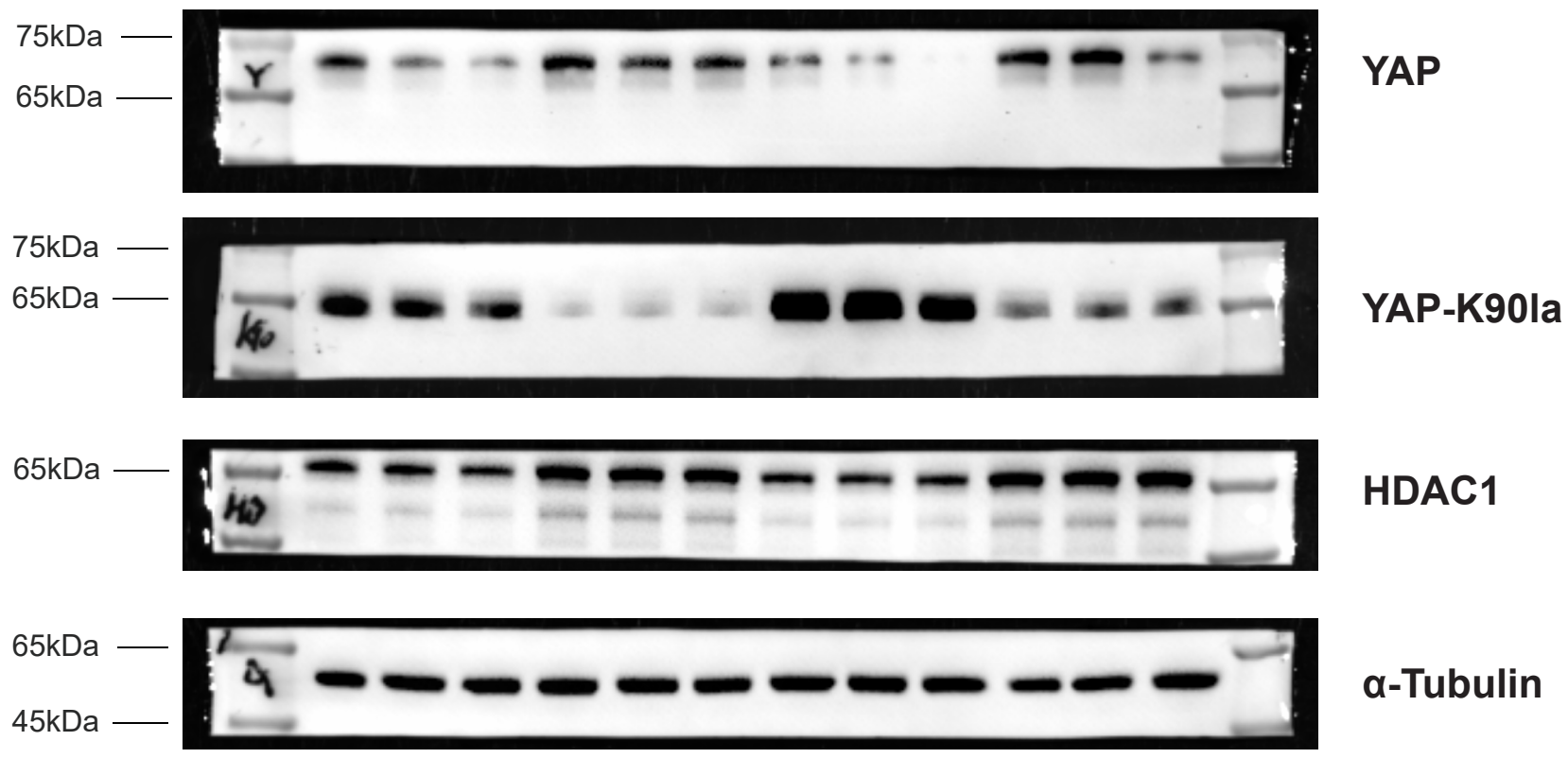

Figure 3F

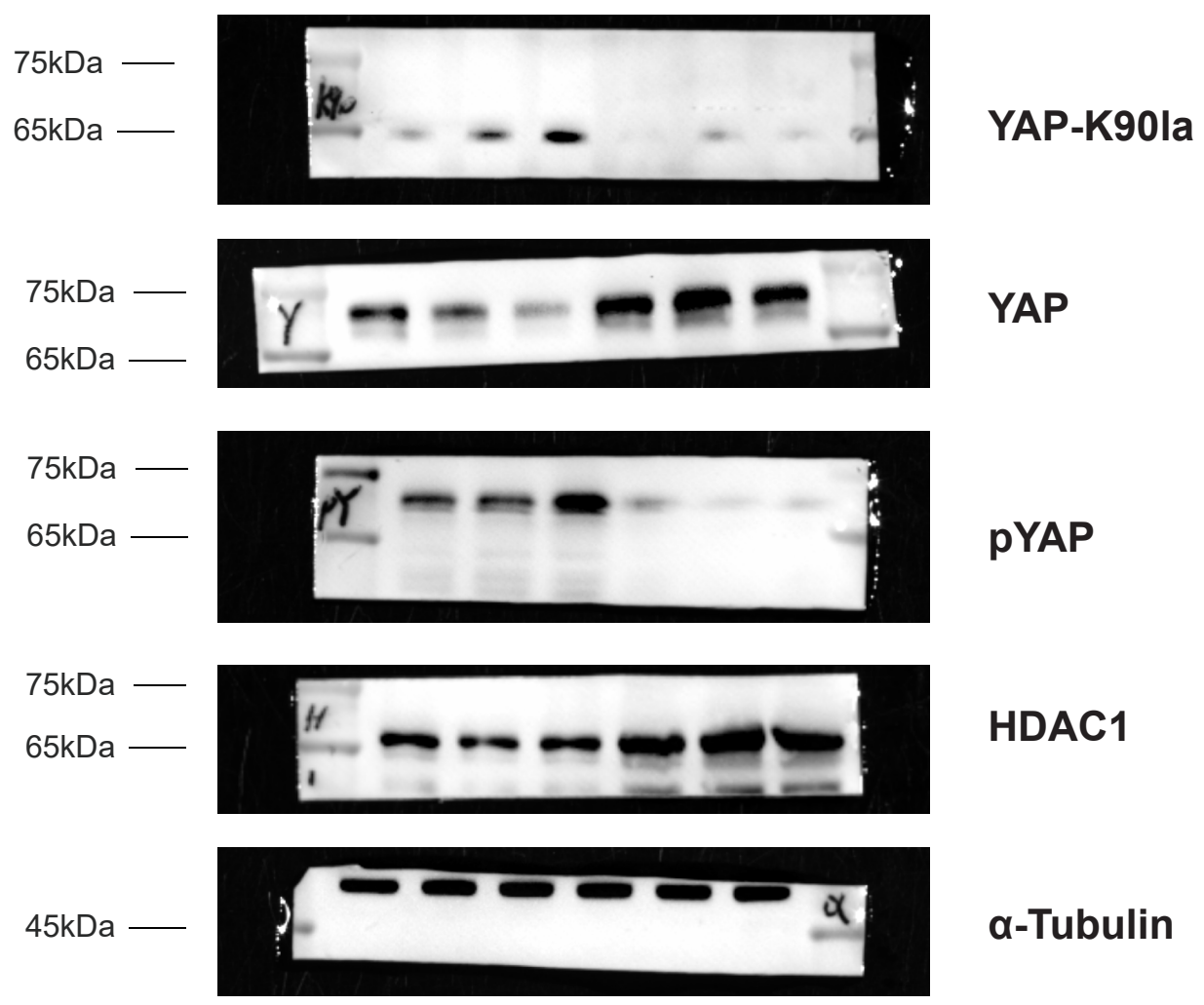

Figure 3G

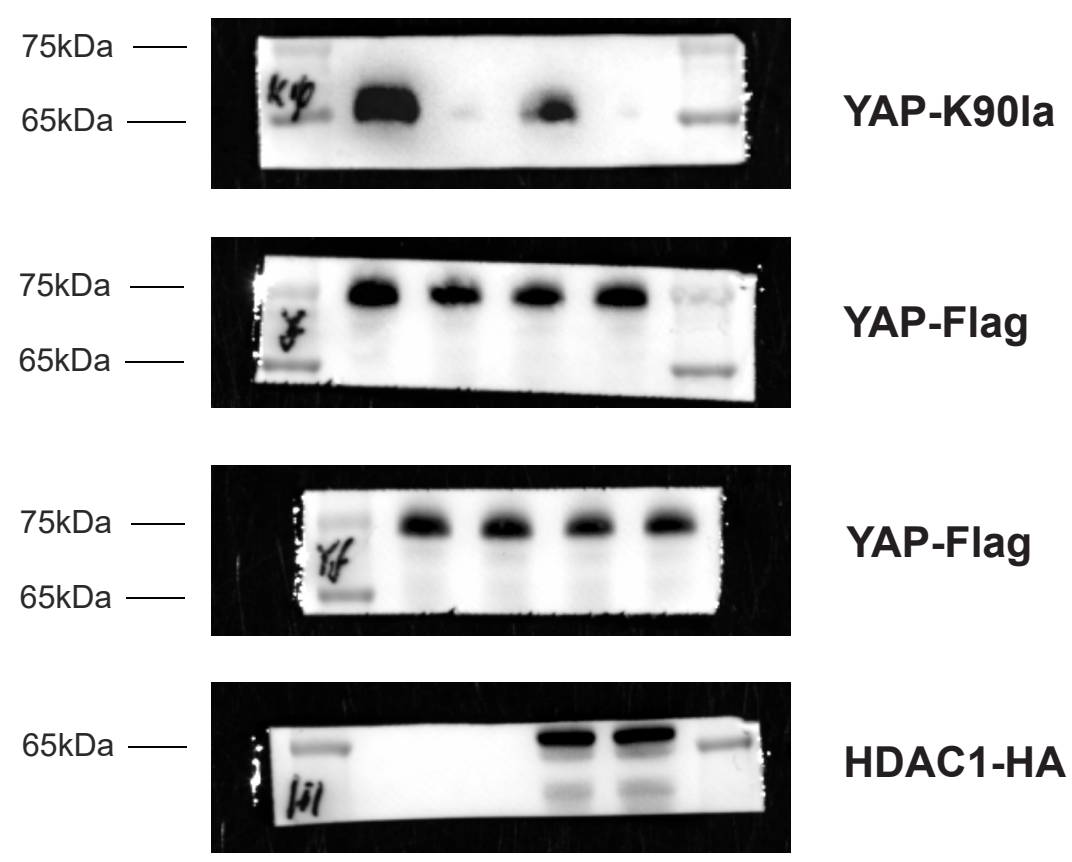

WB images in Figure 4

Figure 4E

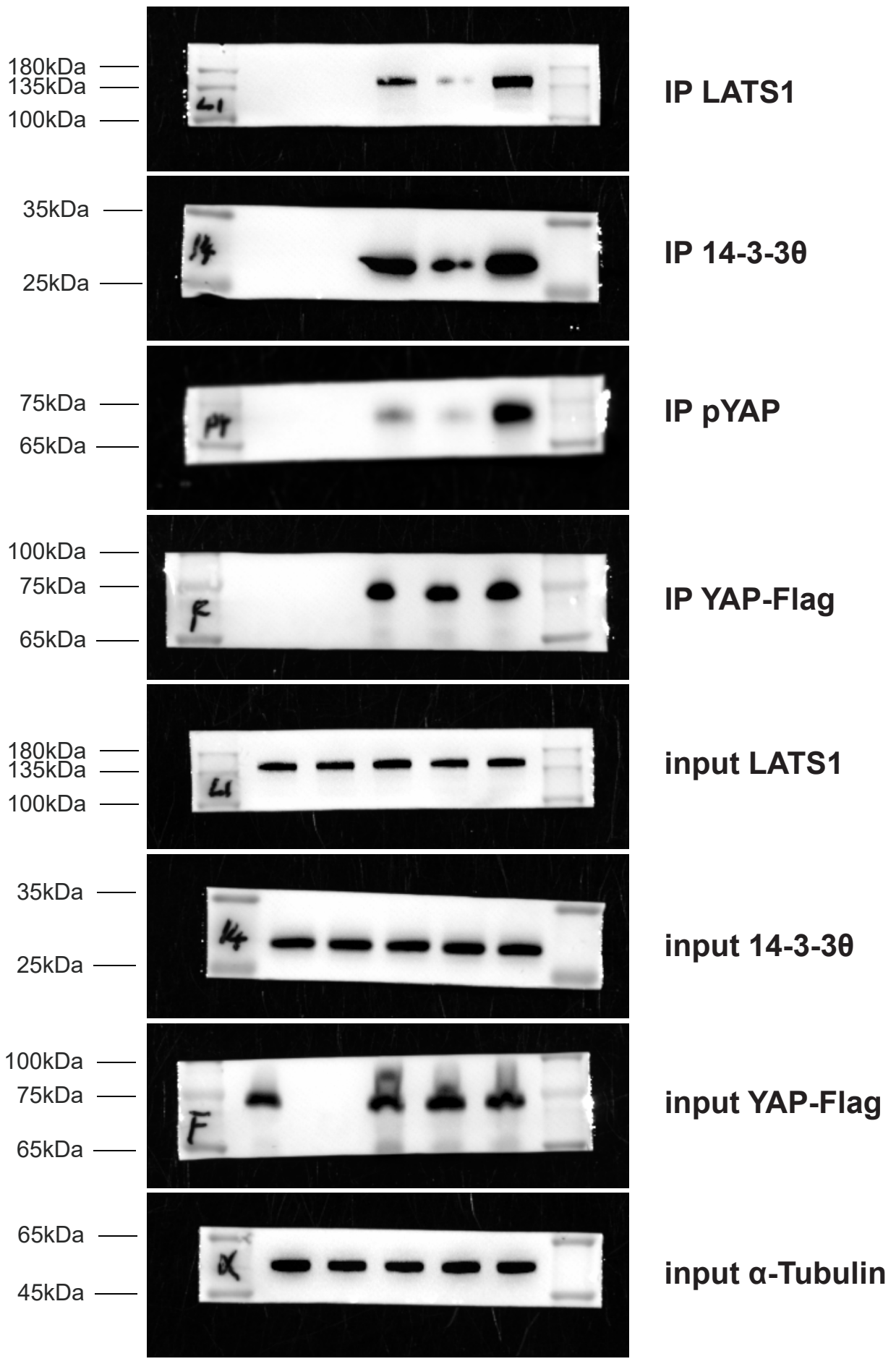

Figure 4F

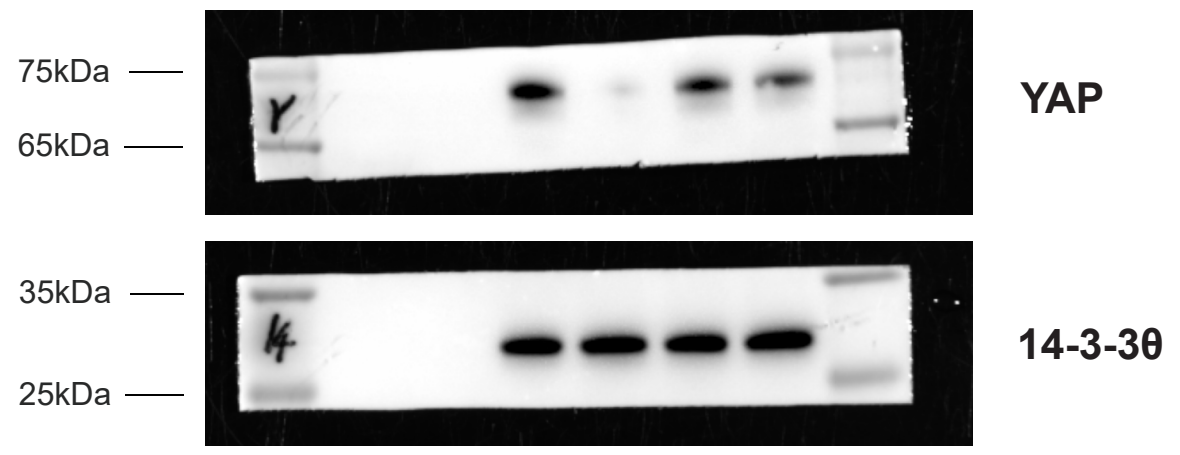

Figure 4G

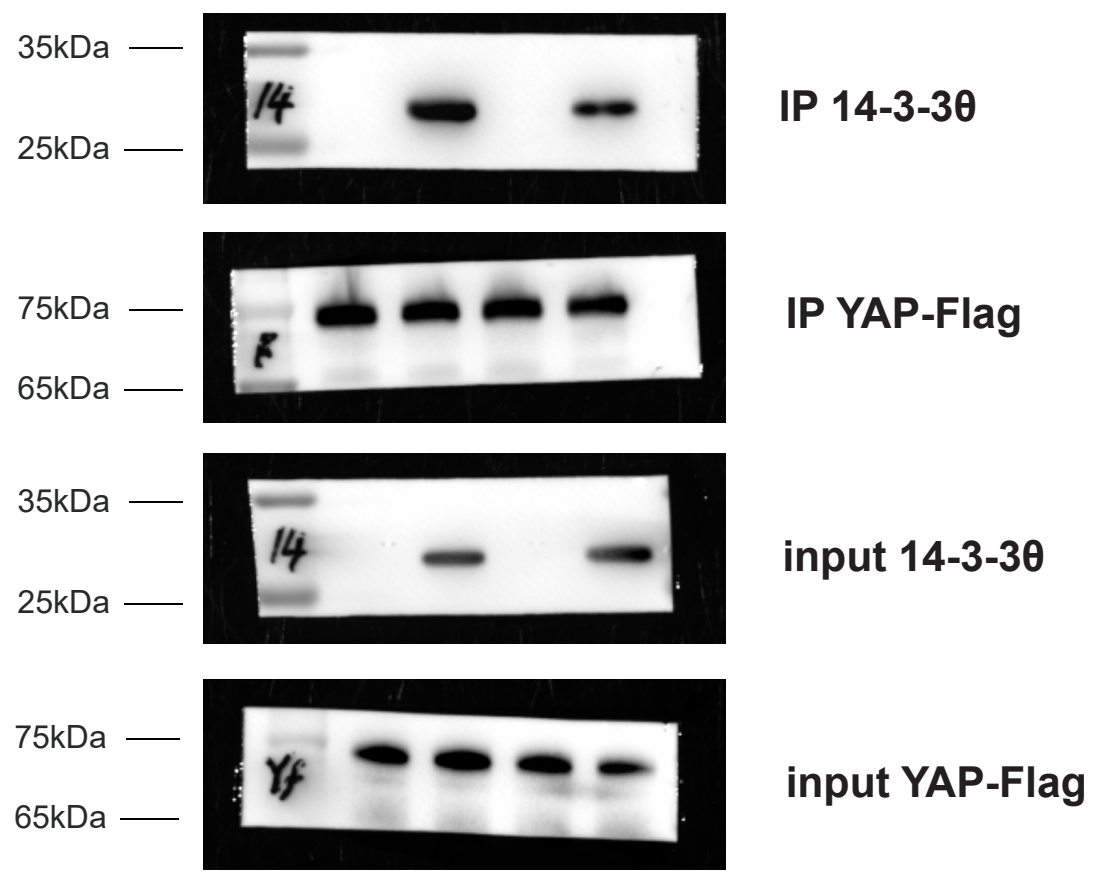

Figure 4H

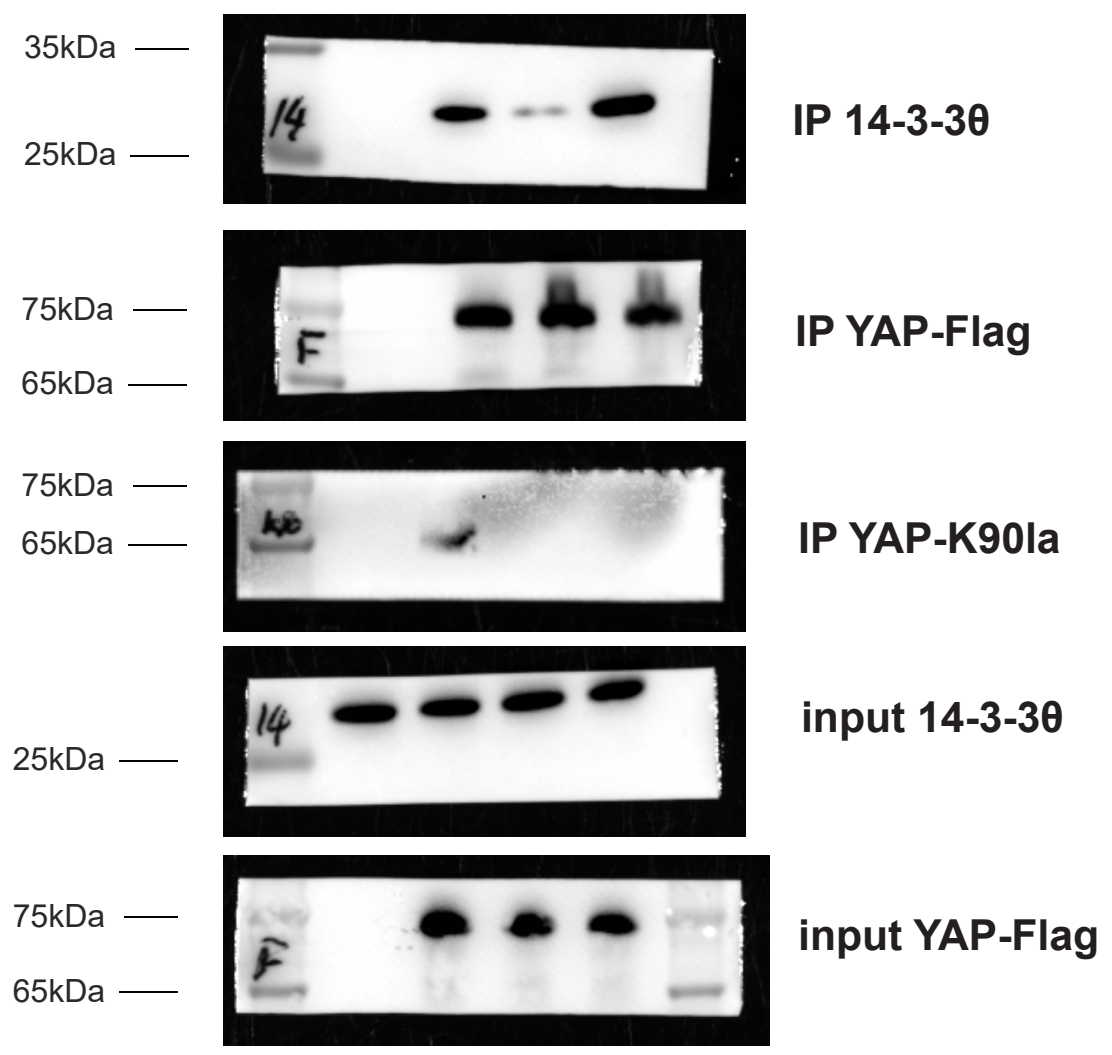

Figure 4S

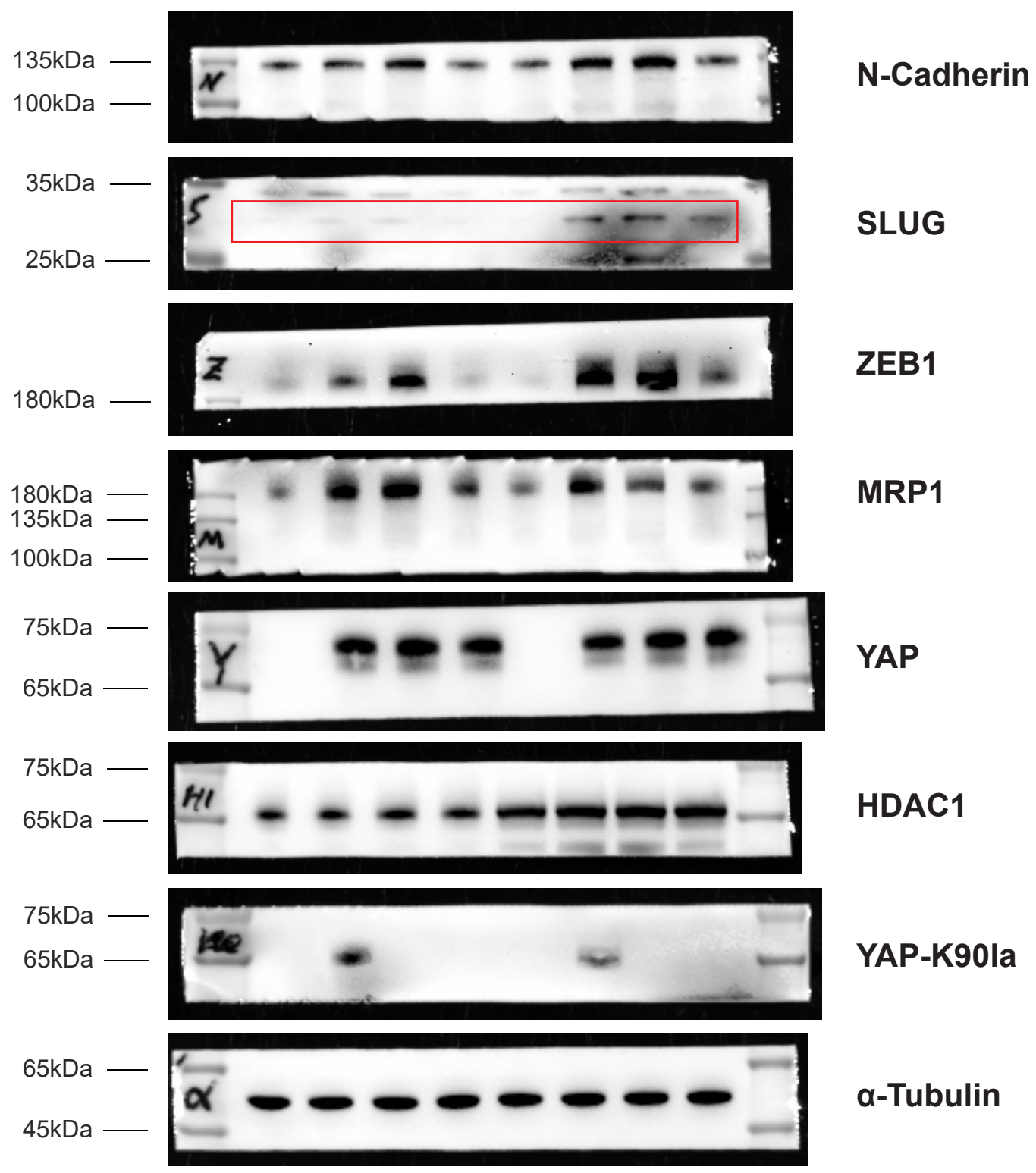

WB images in Figure 7

Figure 7B

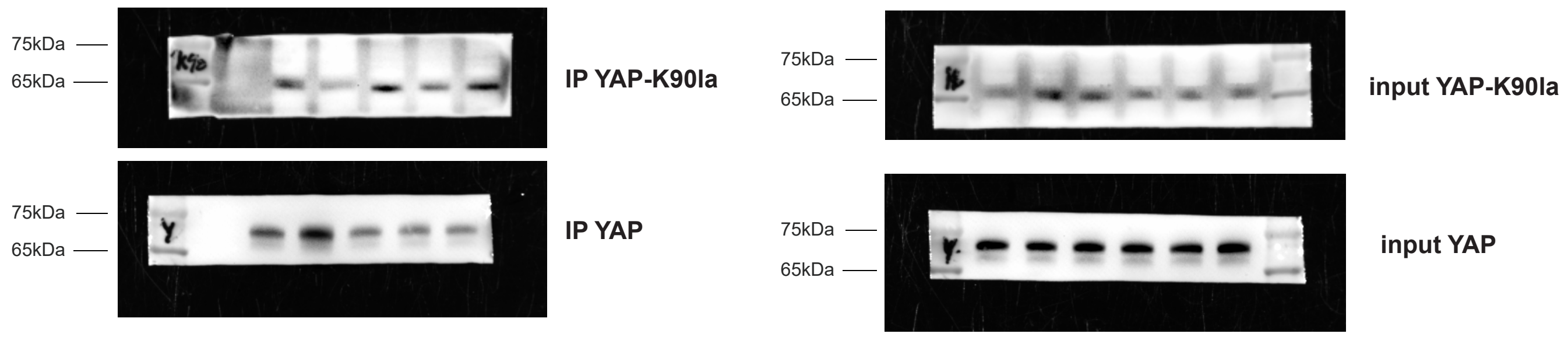

WB images in Figure S1

Figure S1E

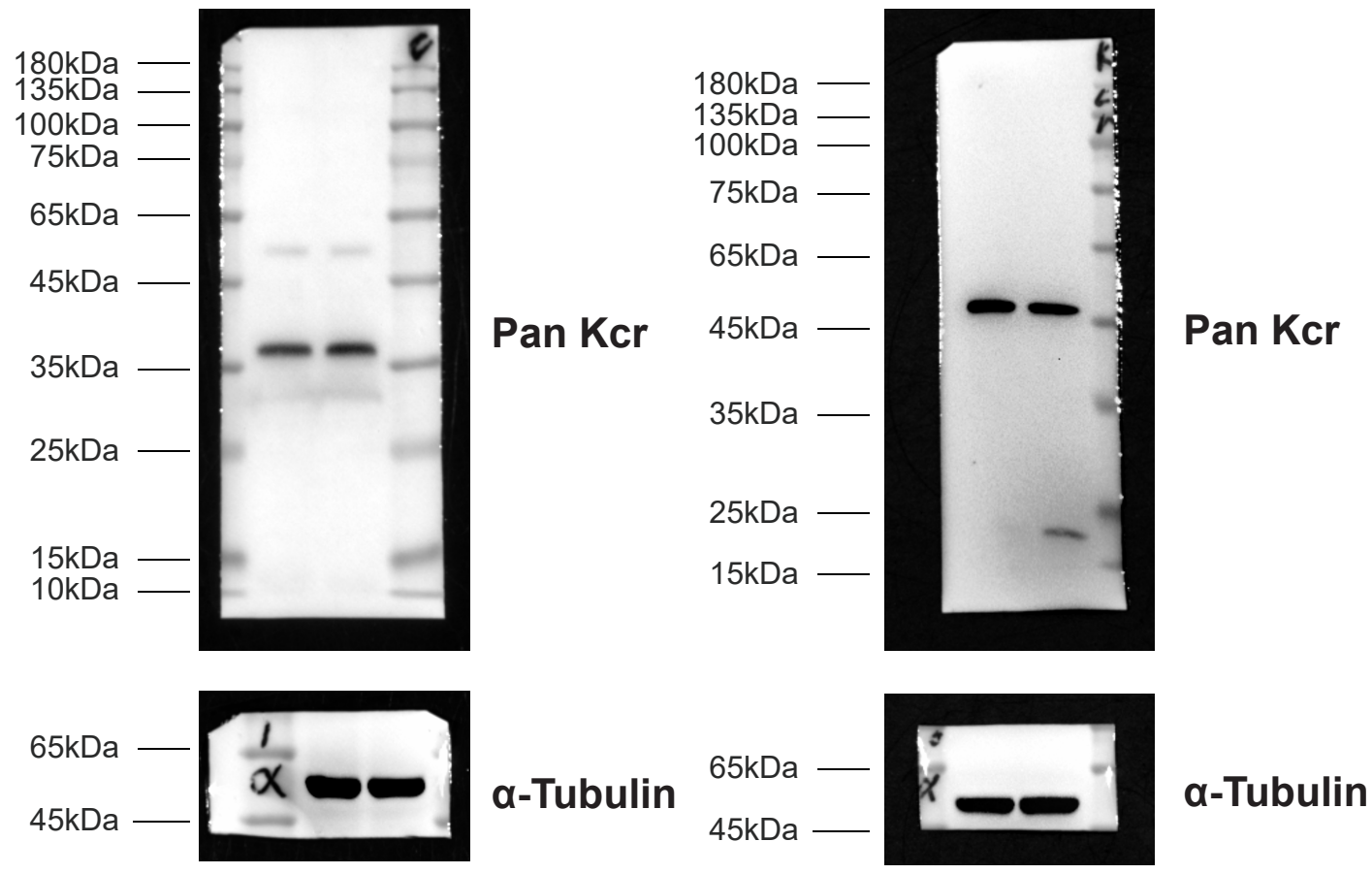

Figure S1F

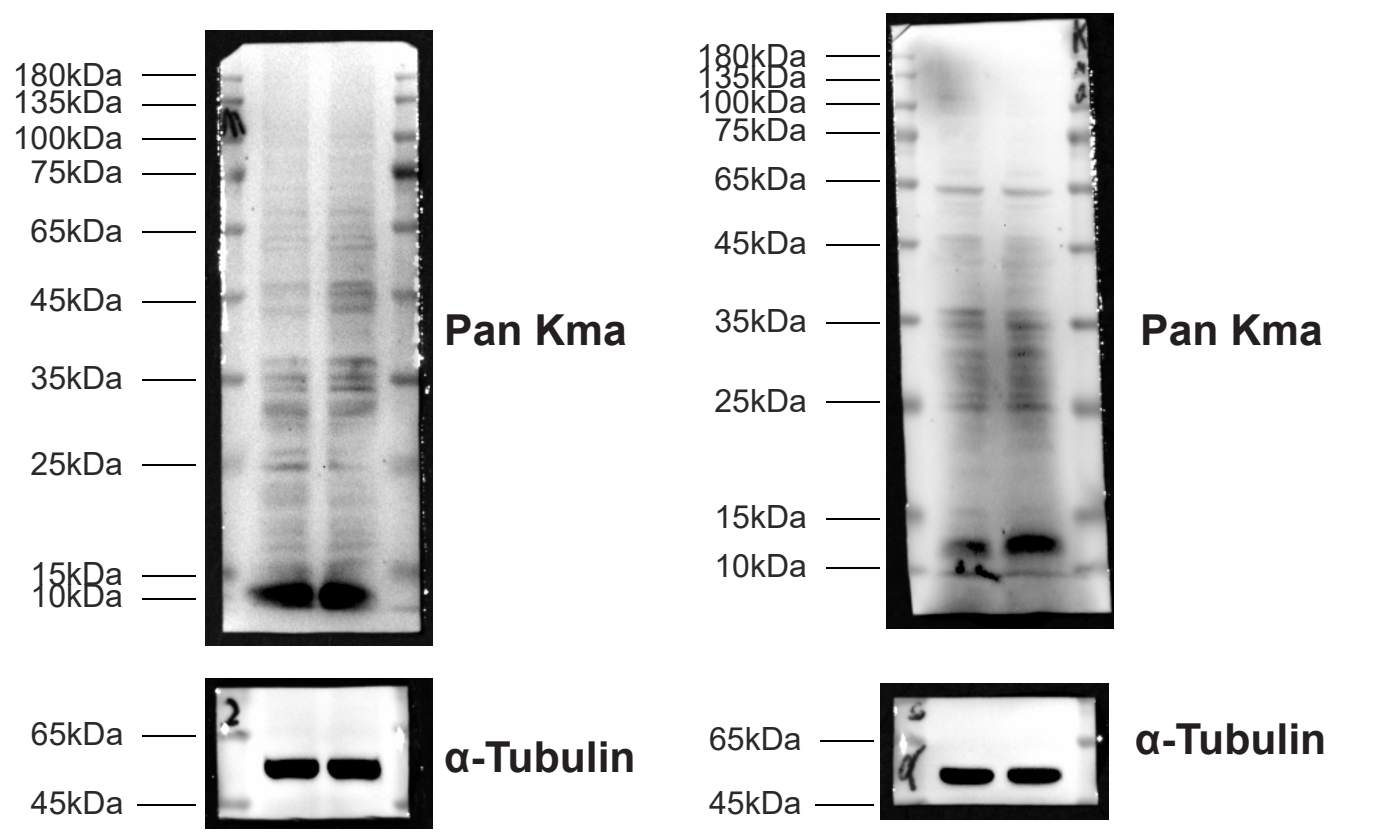

Figure S1G

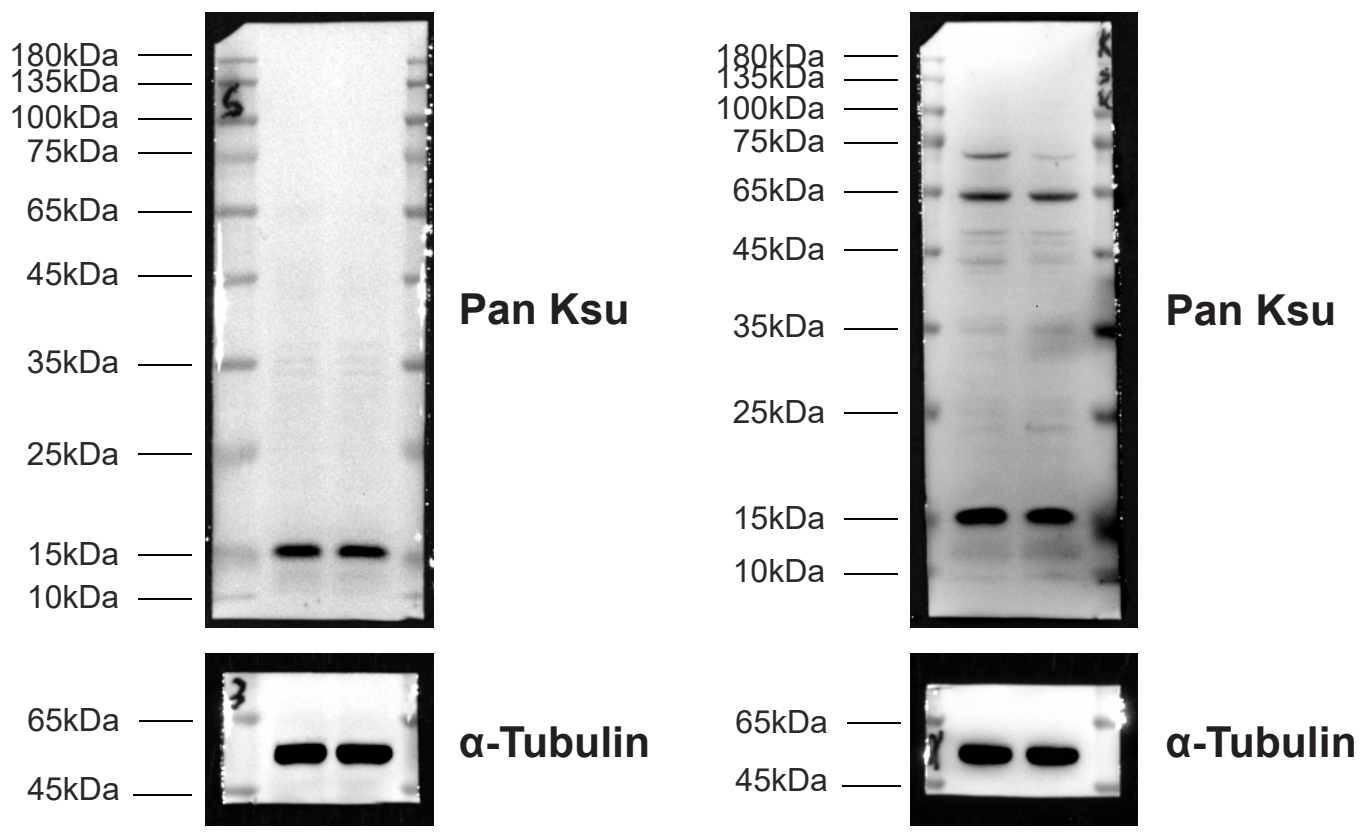

Figure S1O

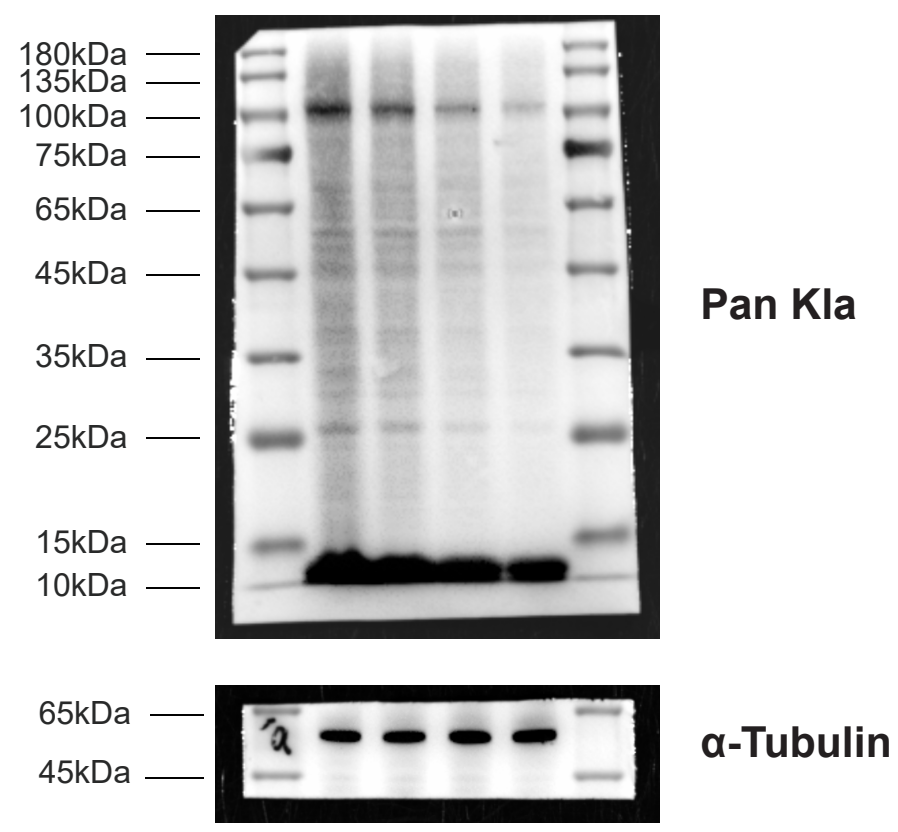

Figure S1P

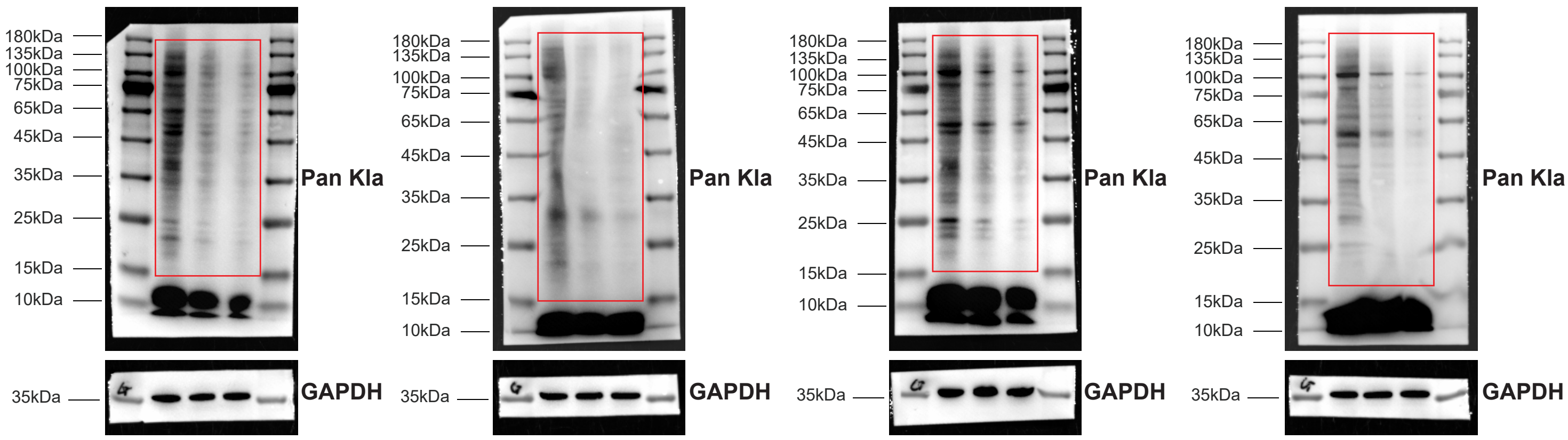

Figure S1Q

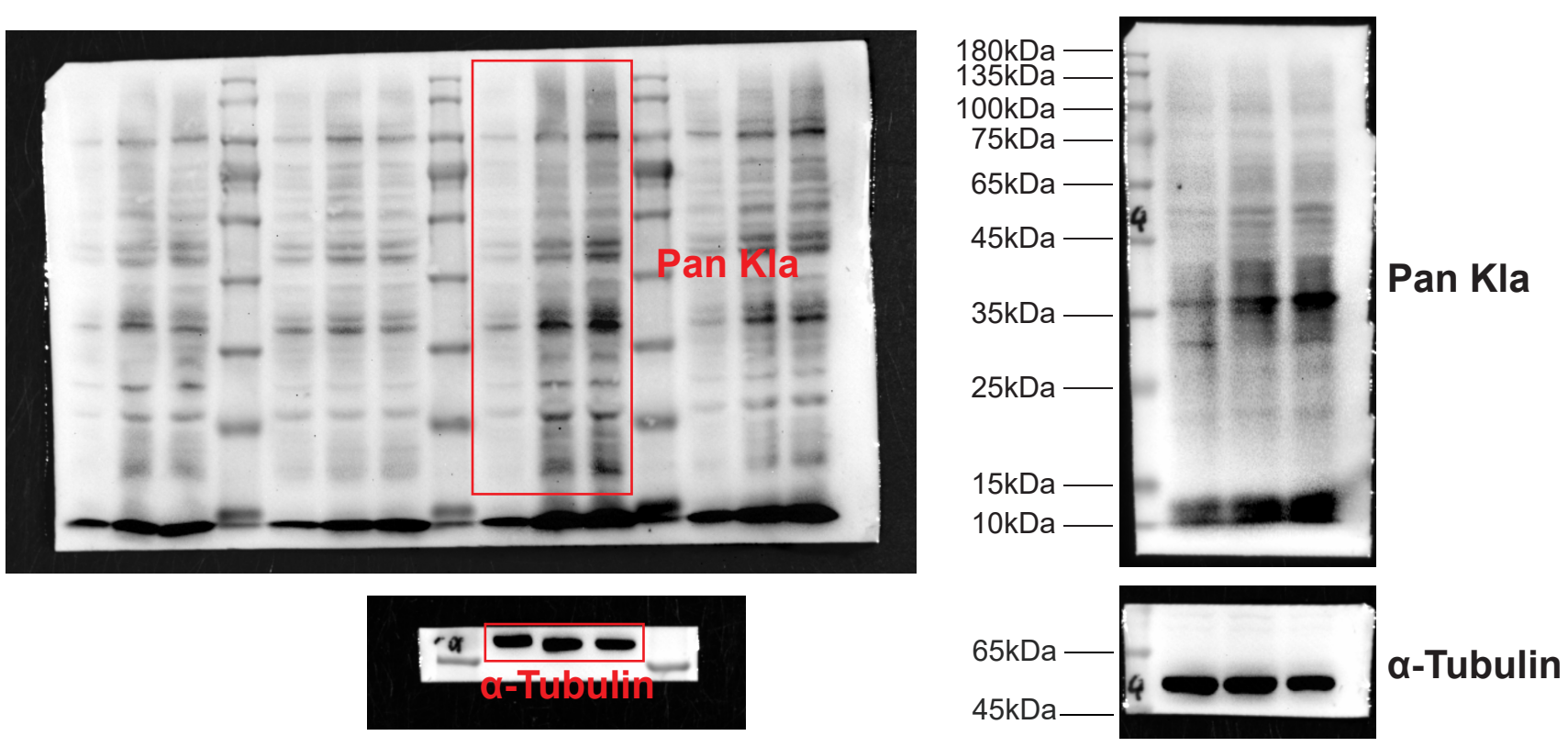

Figure S1R

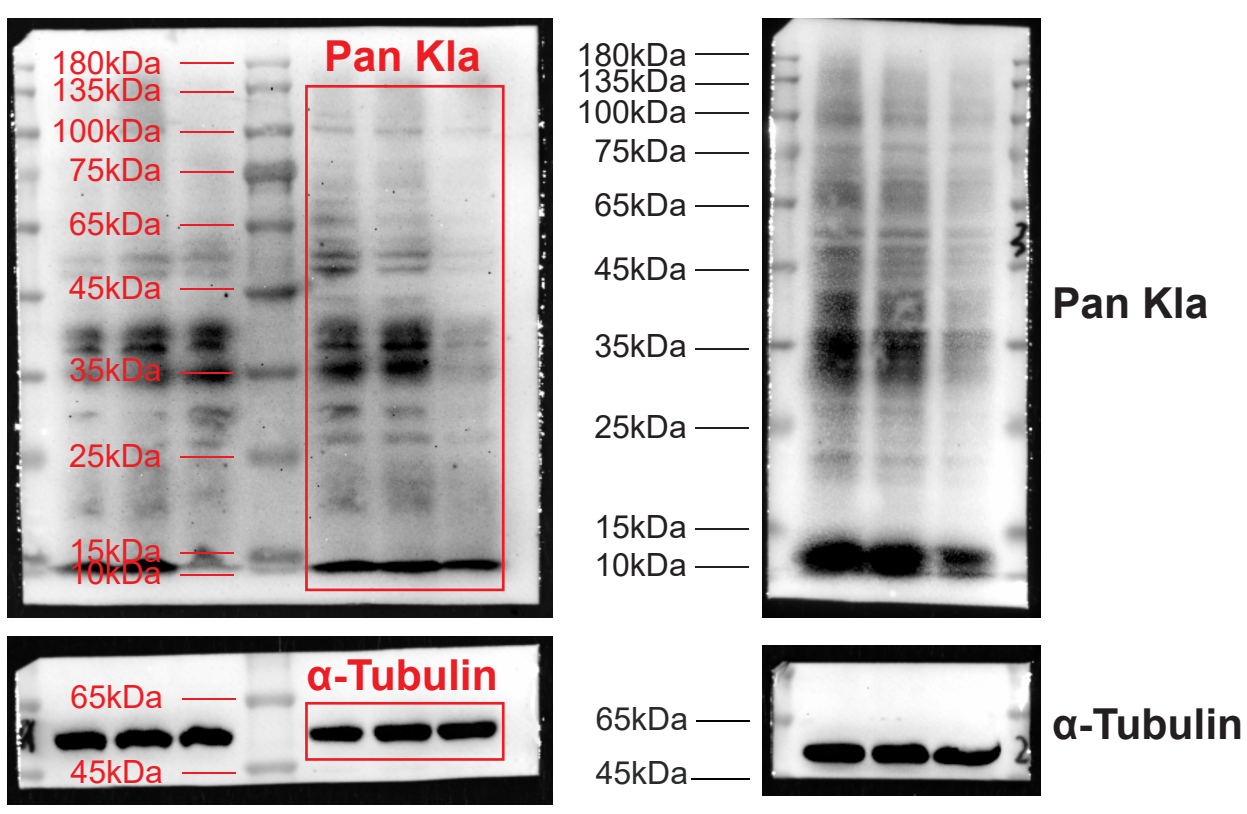

WB images in Figure S2

Figure S2D

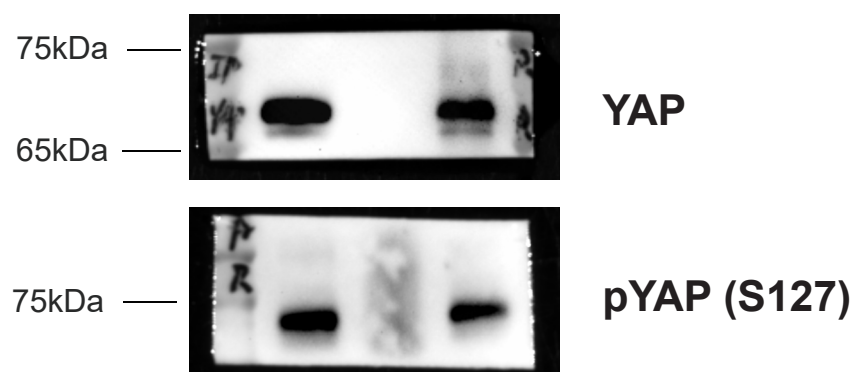

Figure S2E

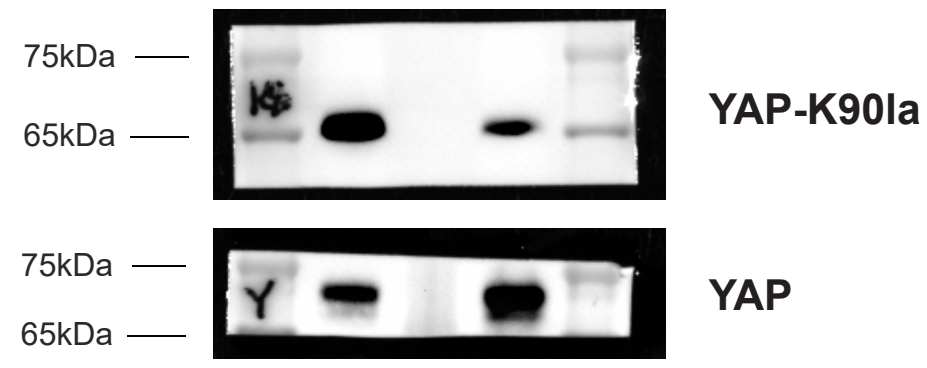

Figure S2F

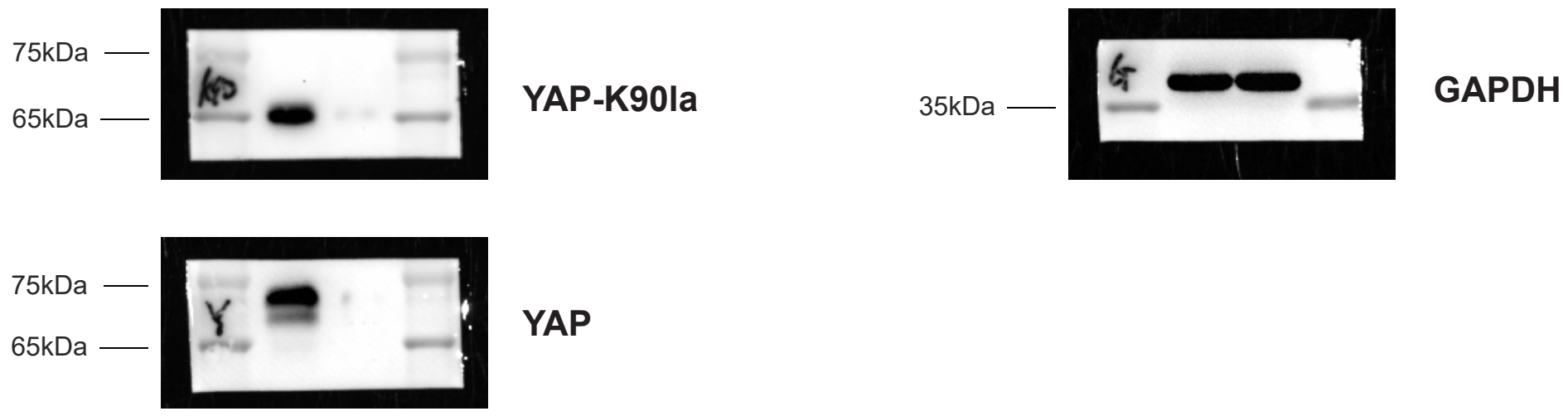

Figure S2M

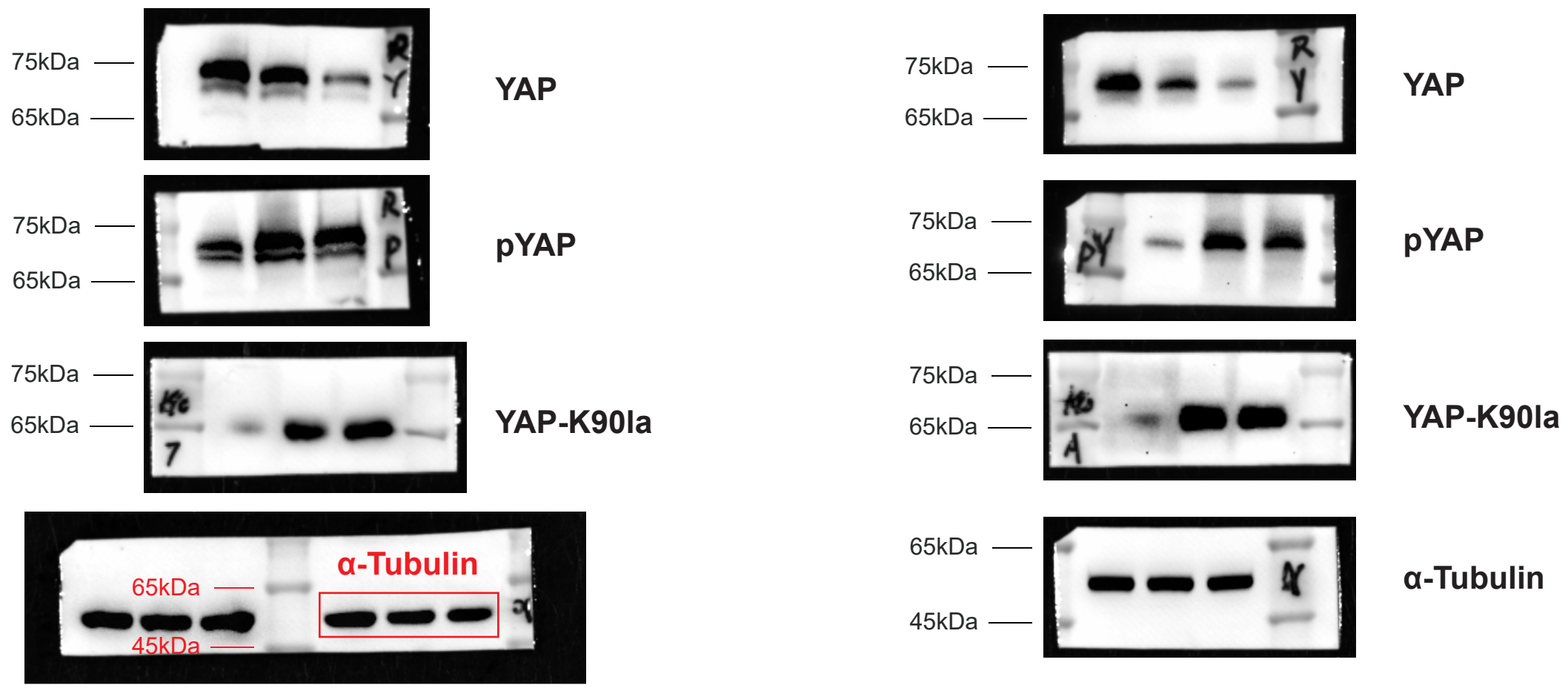

Figure S2N

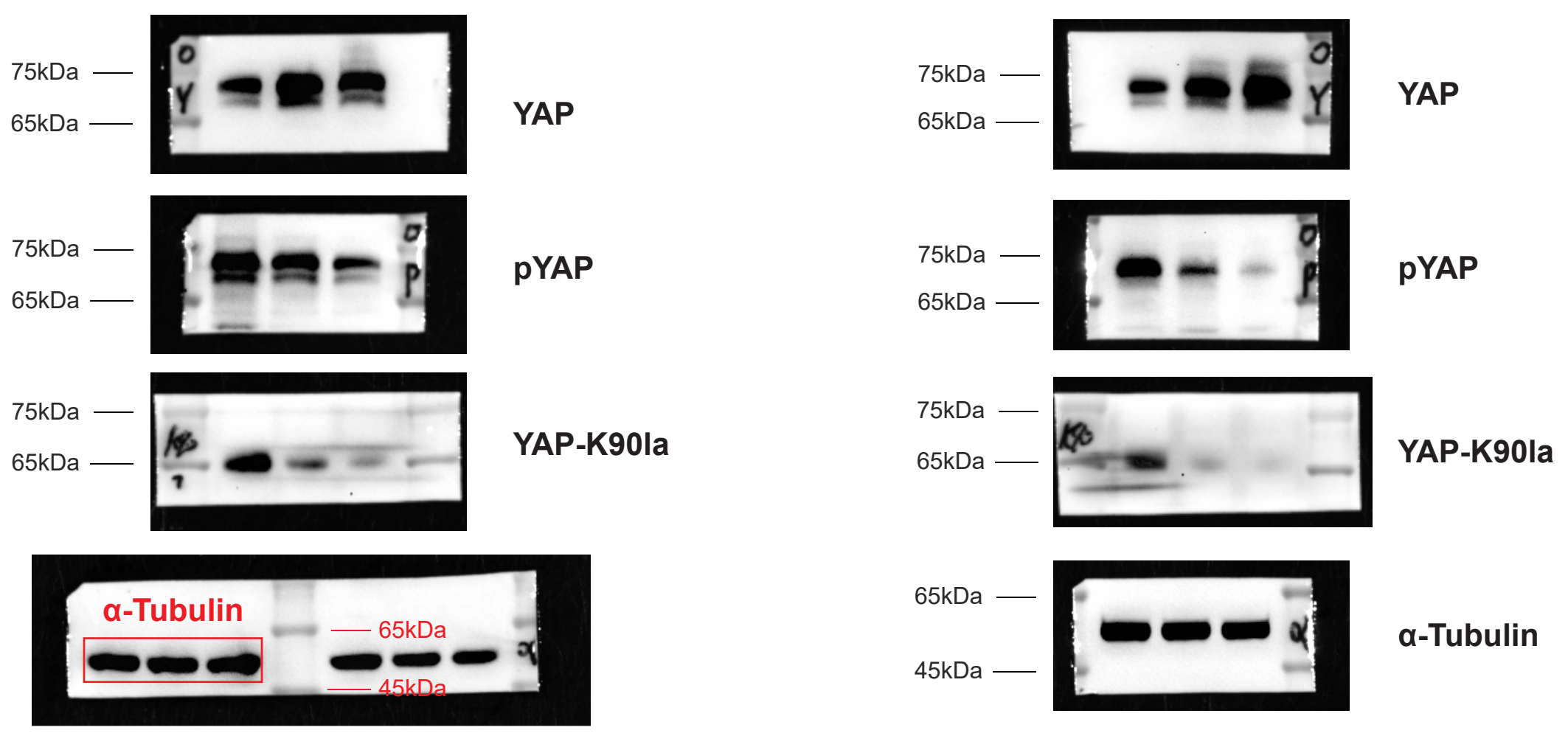

WB images in Figure S3

Figure S3A

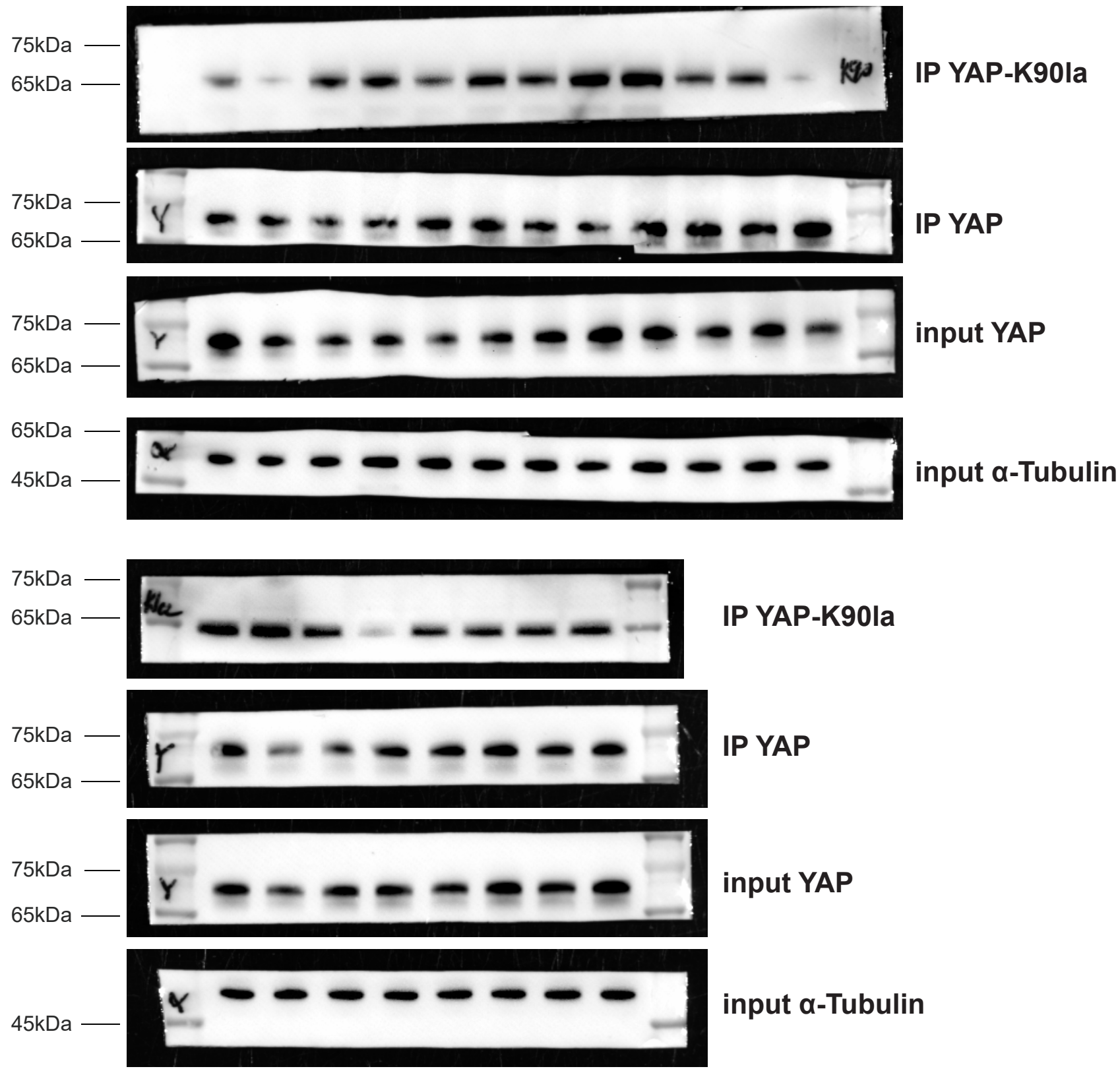

Figure S3B

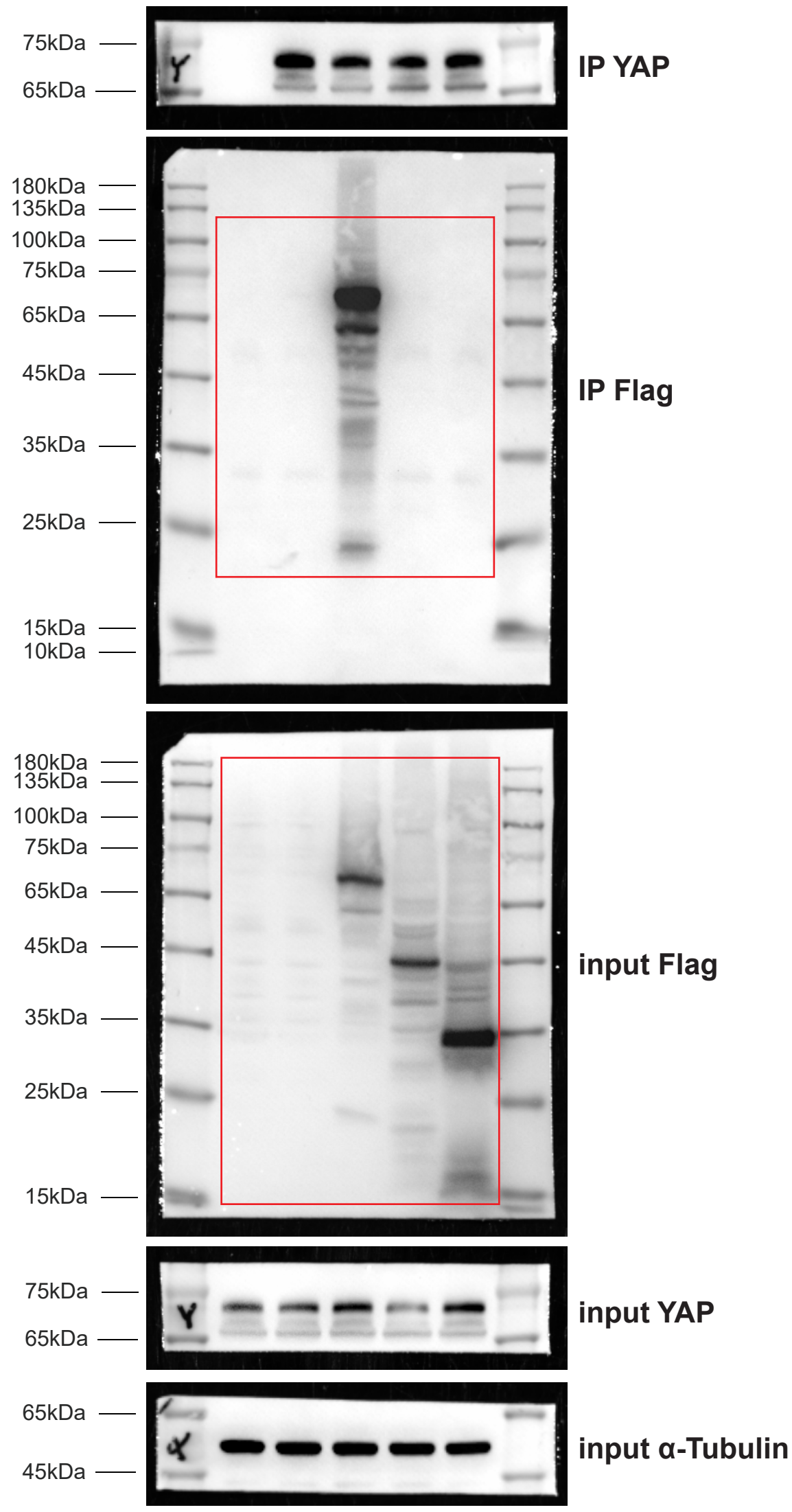

Figure S3C

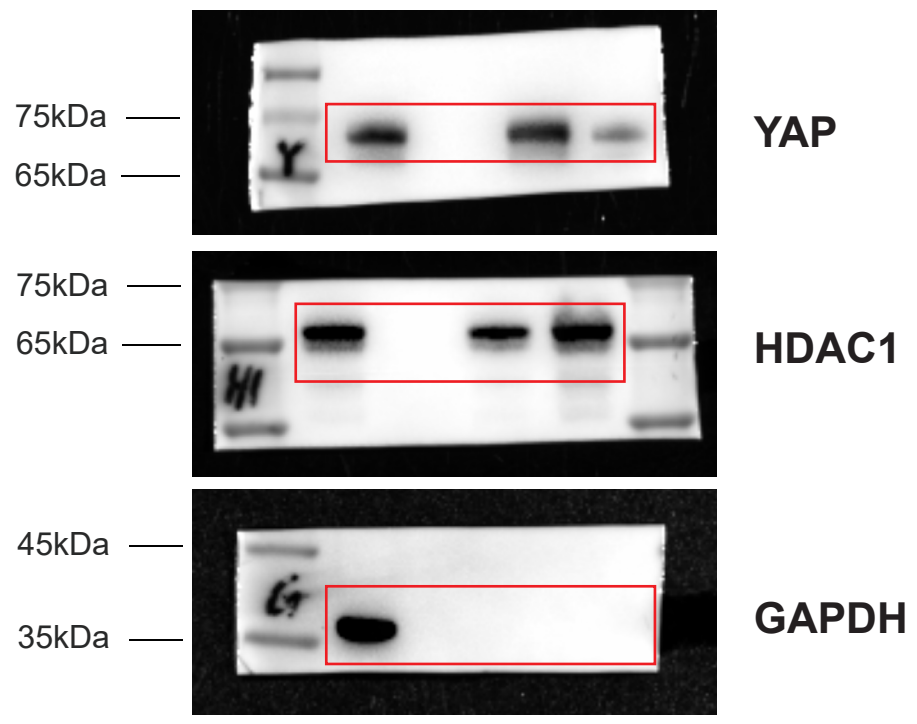

Figure S3F

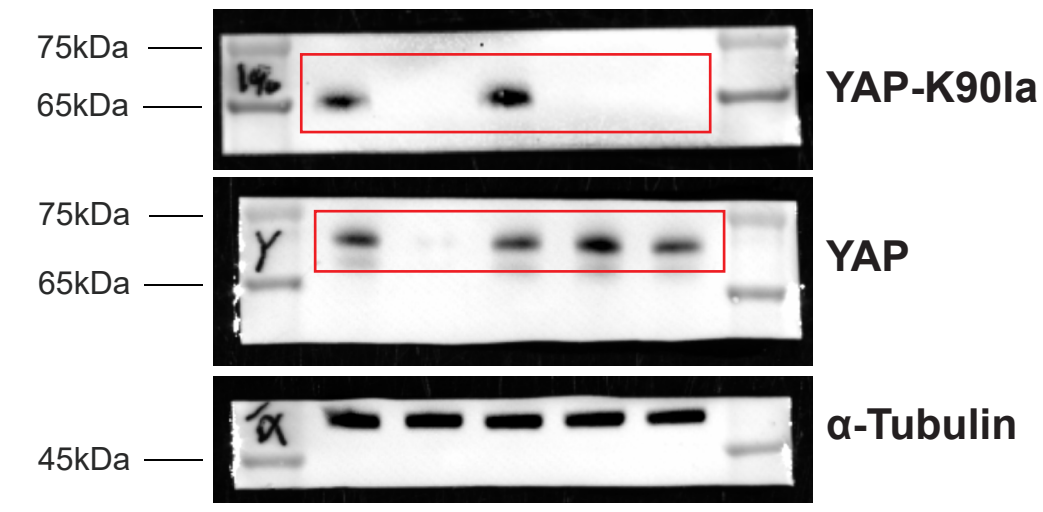

Figure S3E

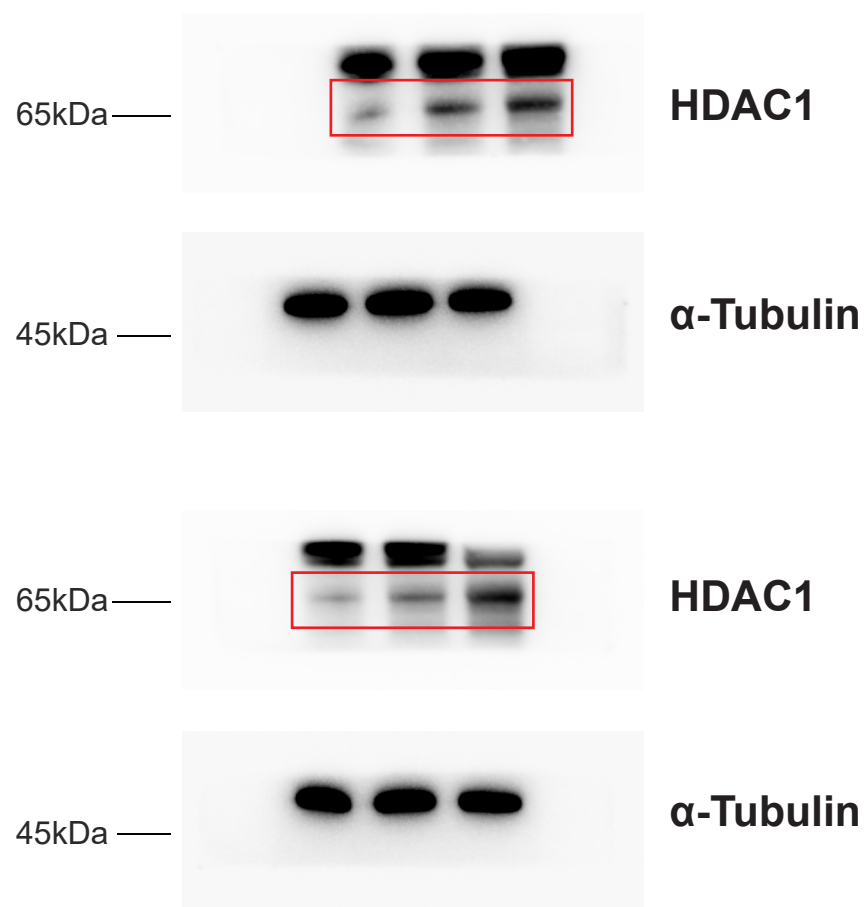

Figure S3G

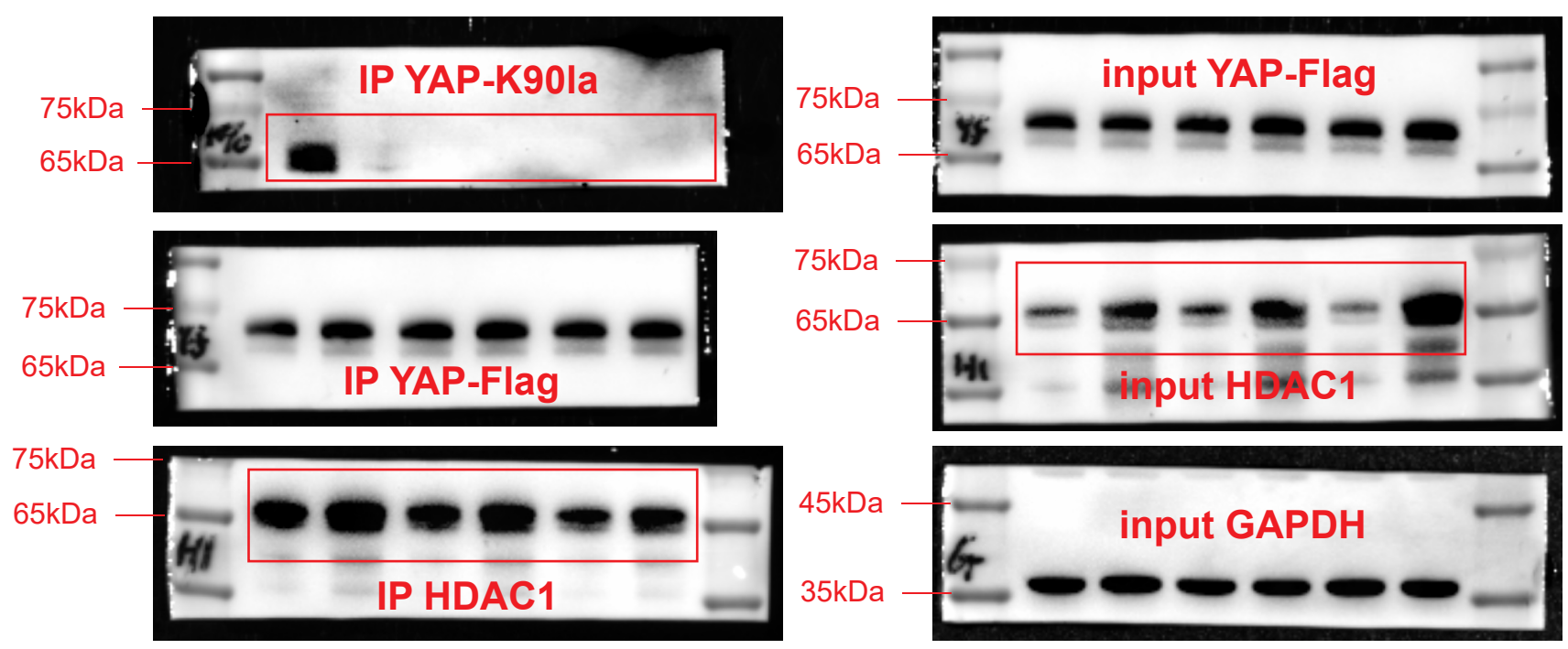

WB images in Figure S4

Figure S4G

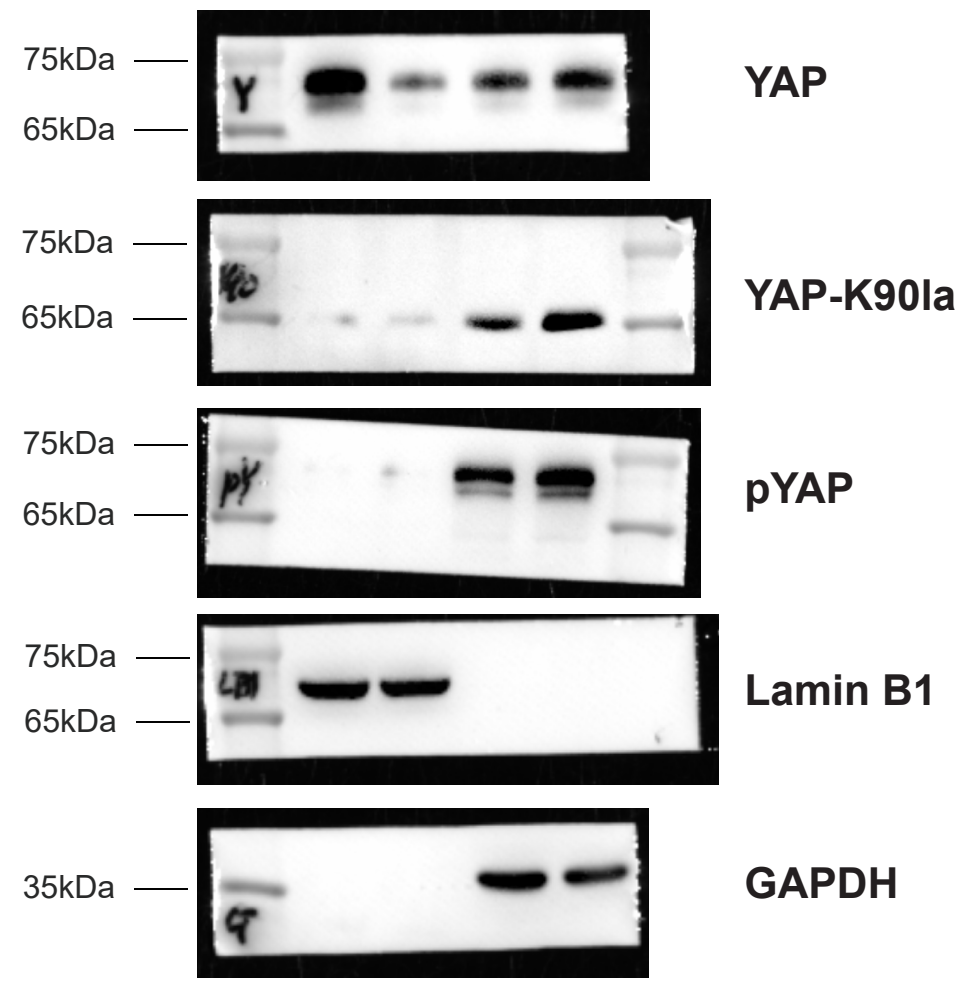

Figure S4H

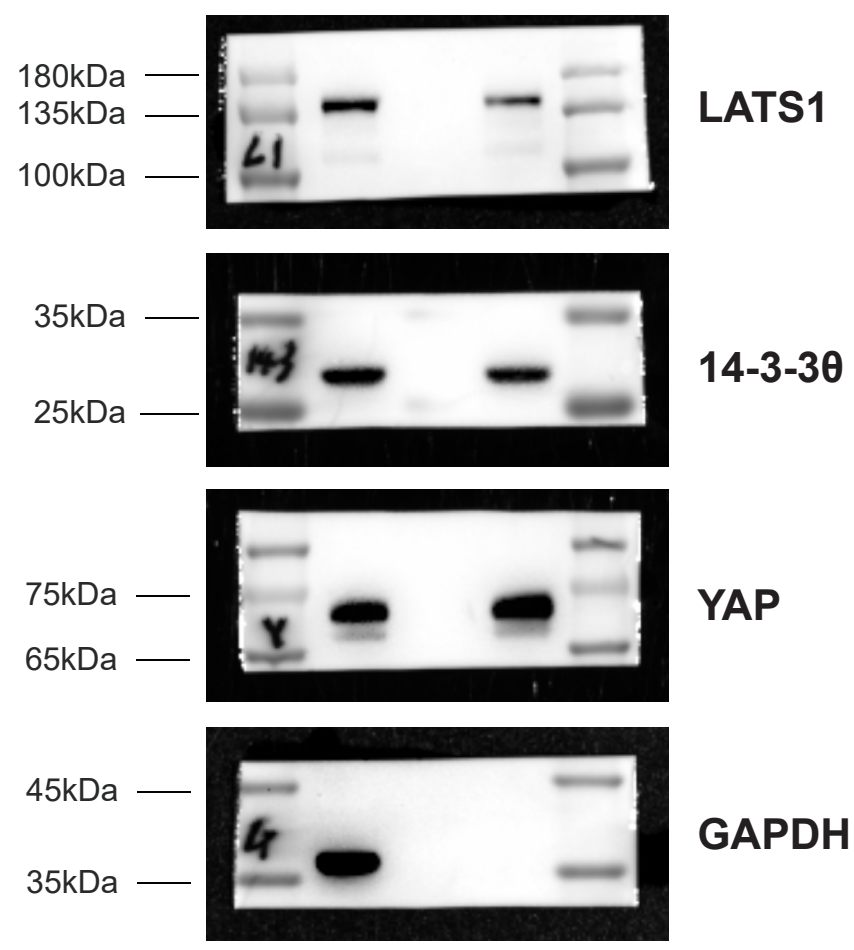

Figure S4I

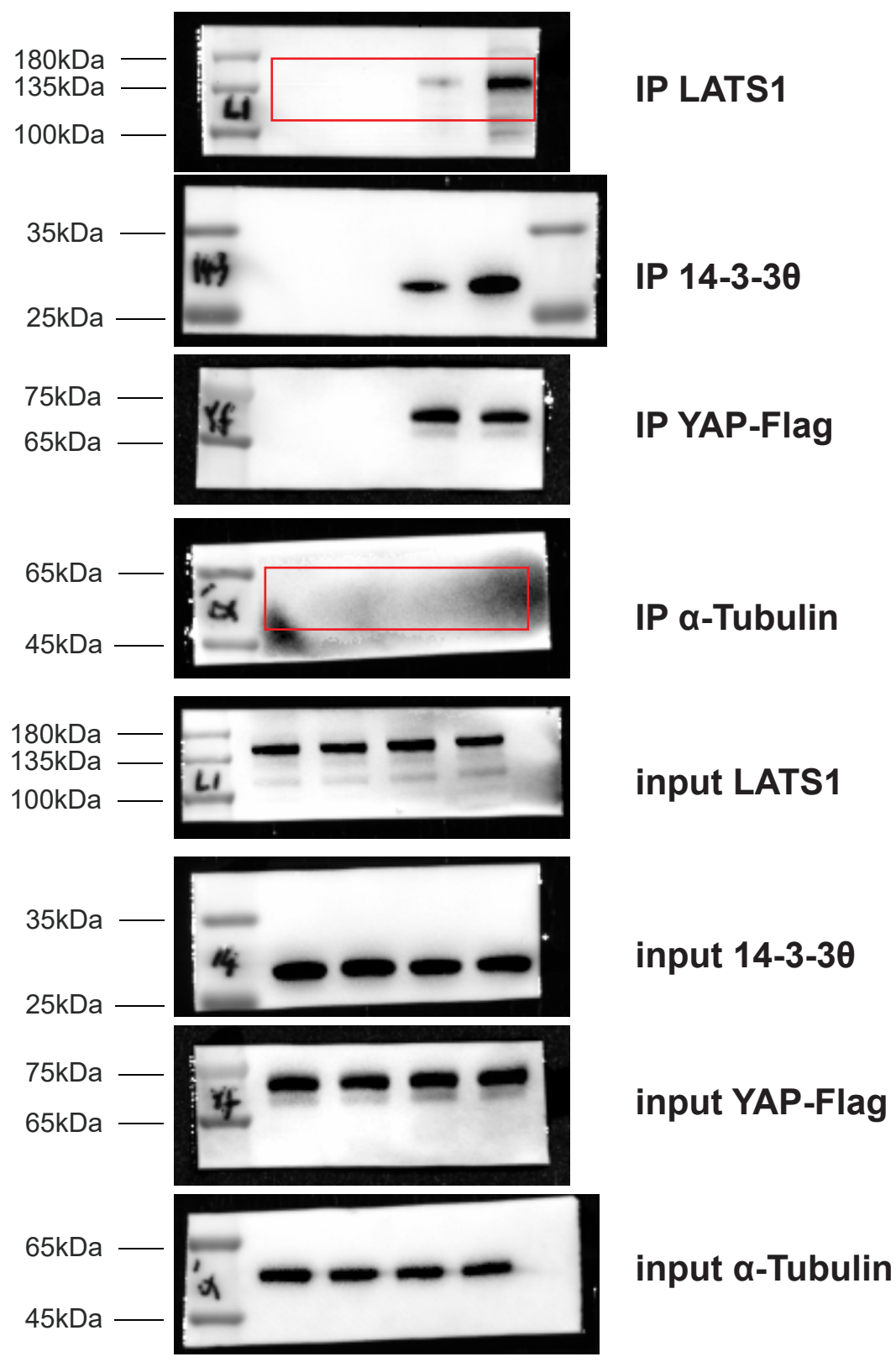

Figure S4J

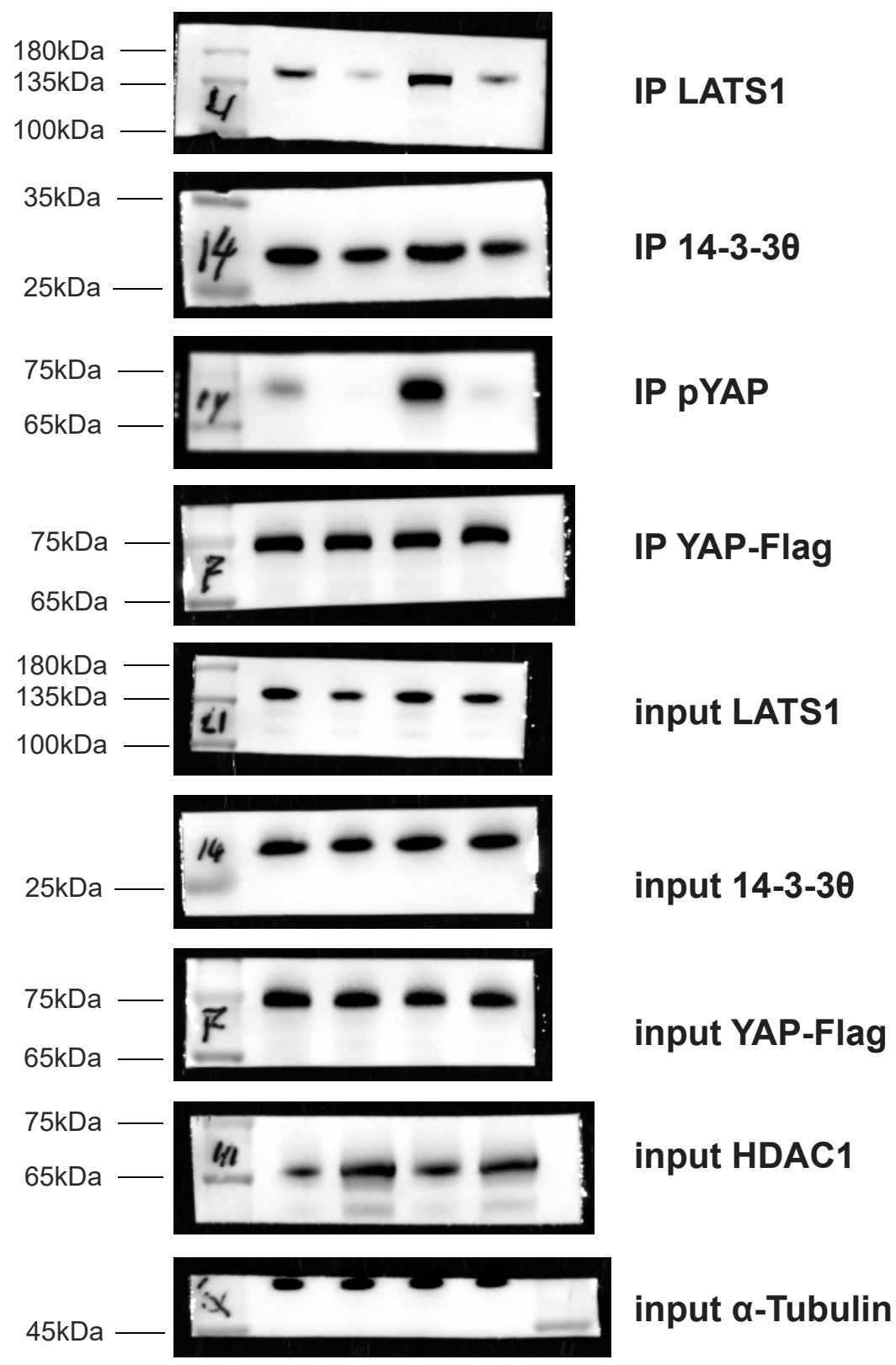

Figure S4K

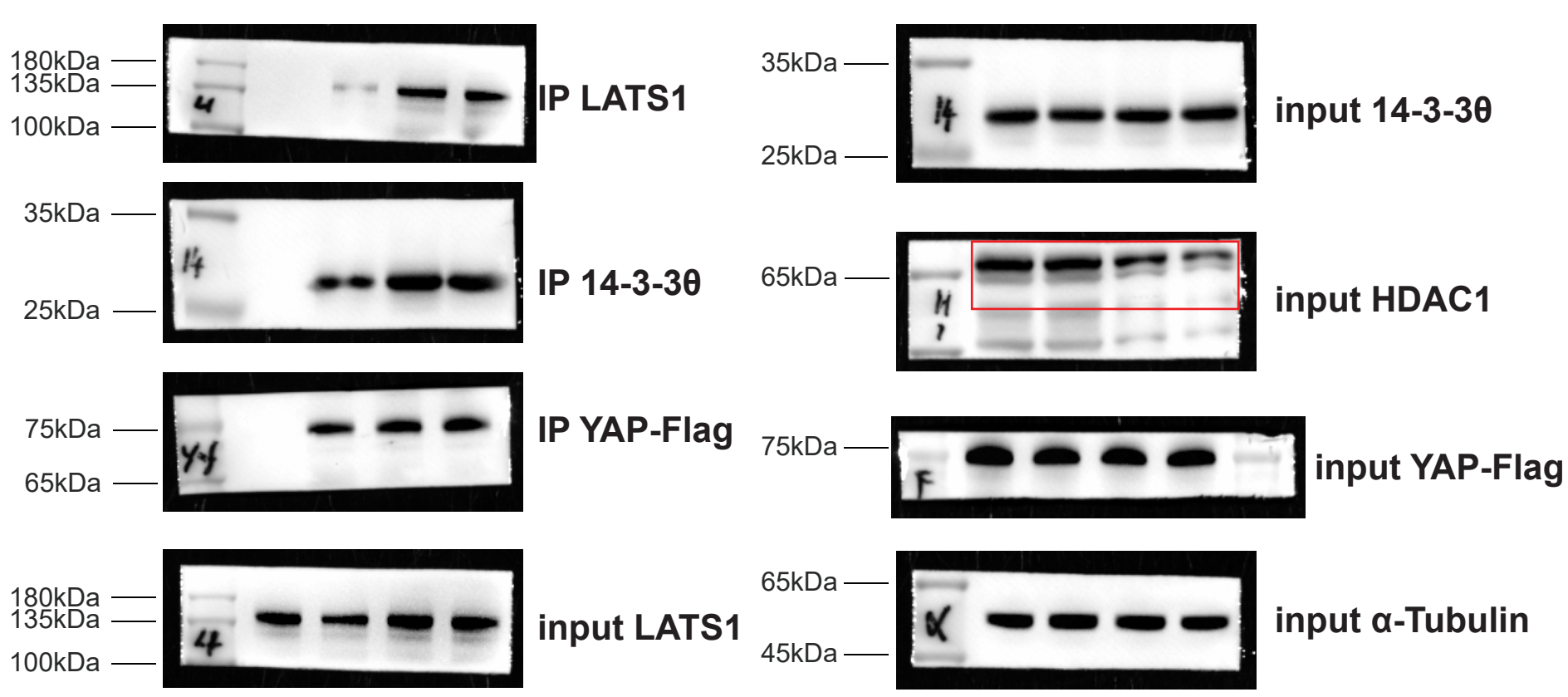

Figure S4L

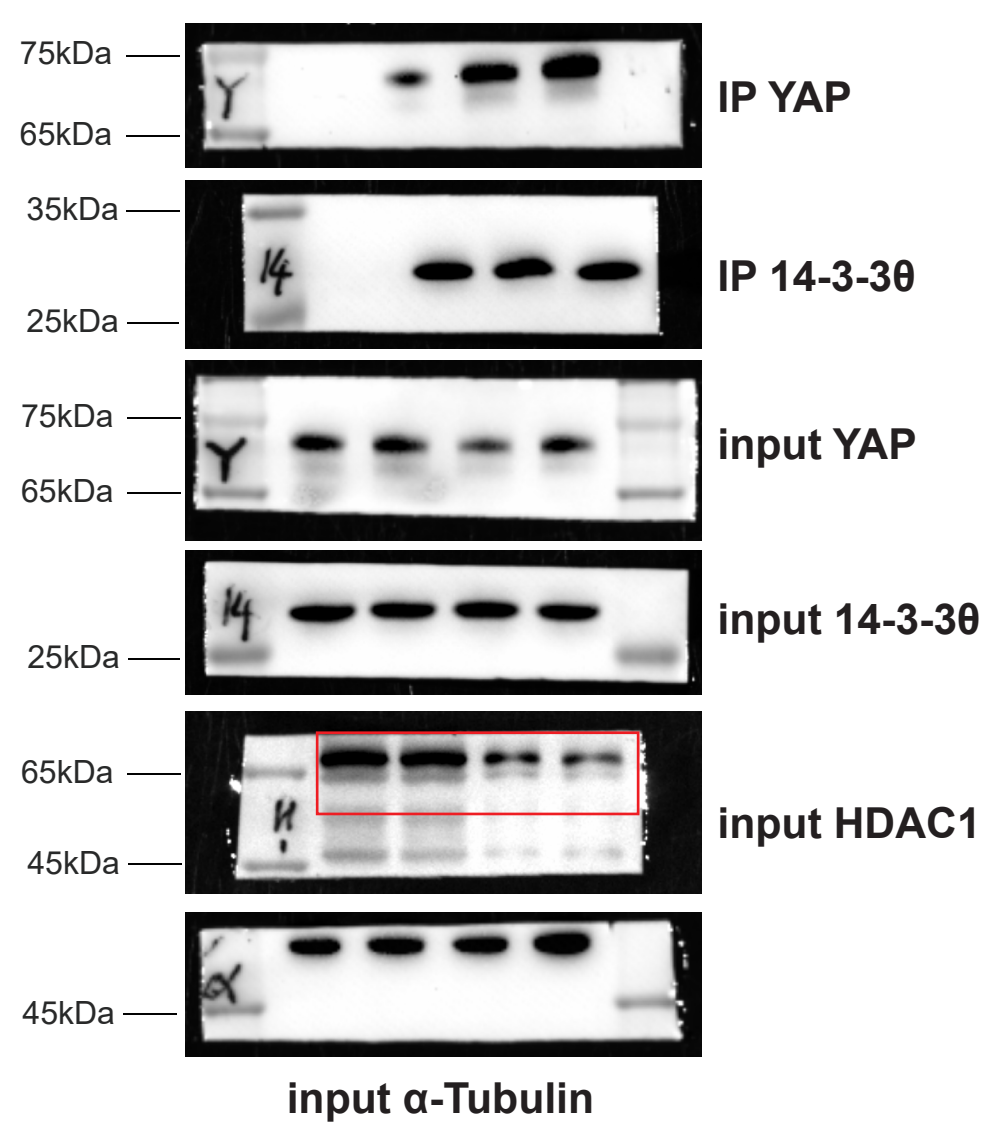

WB images in Figure S5

Figure S5D

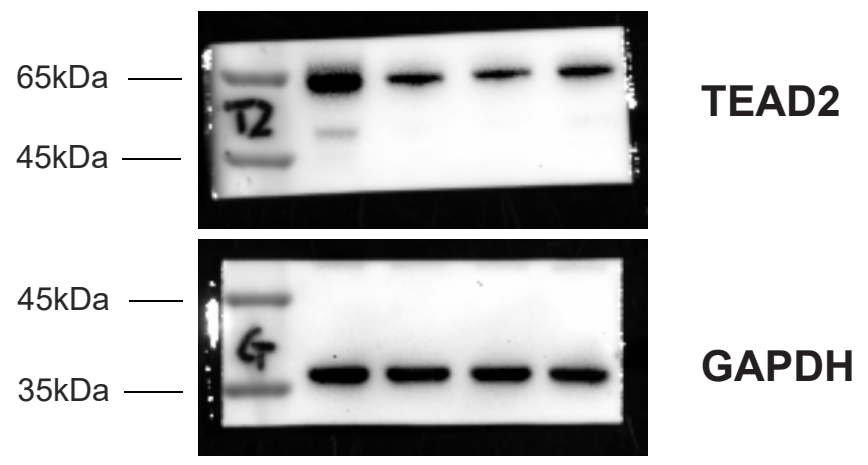

Figure S5E

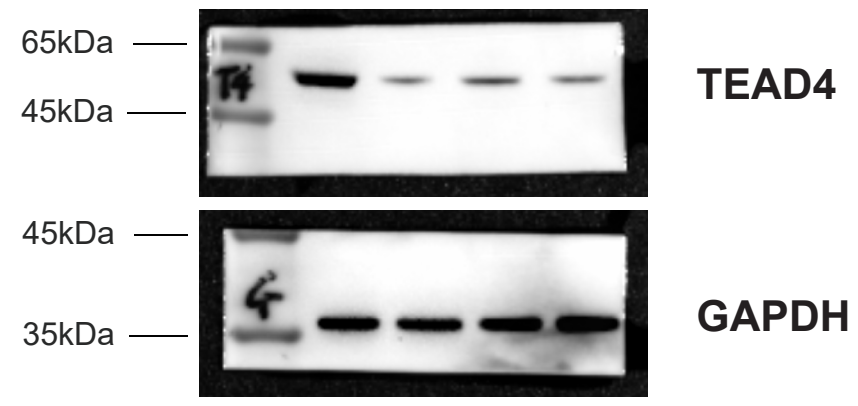

Figure S5H

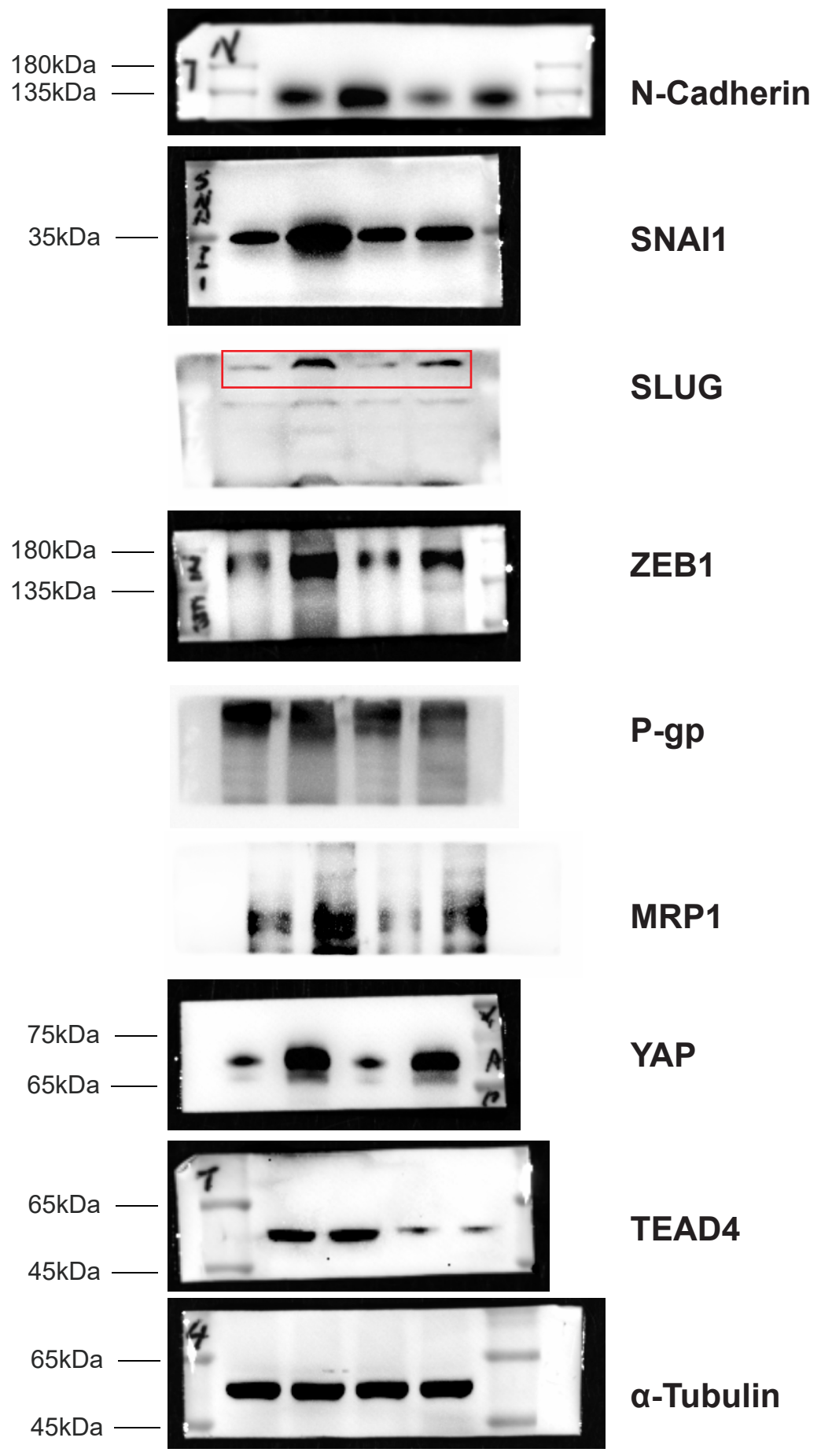

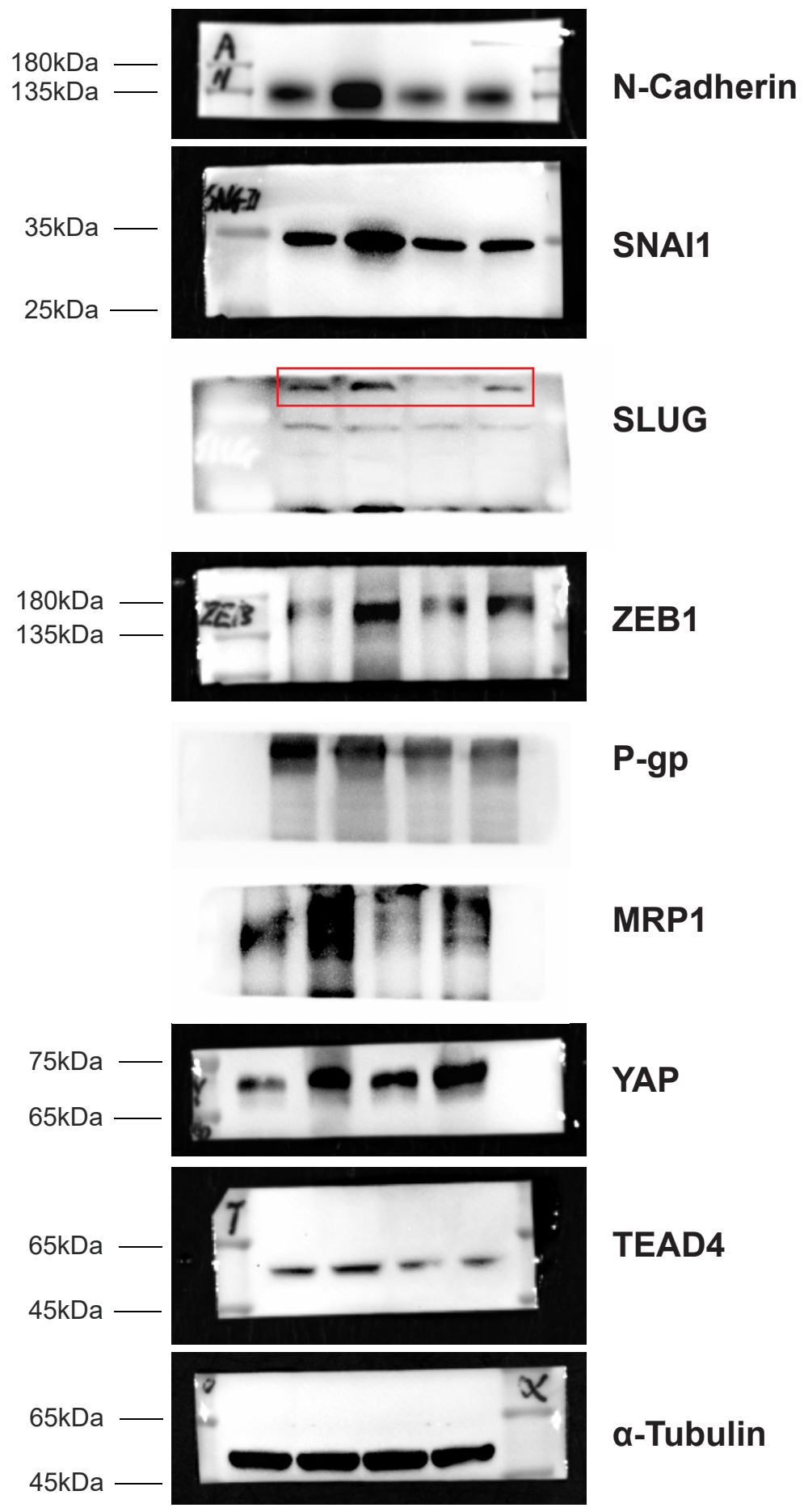

Figure S5L

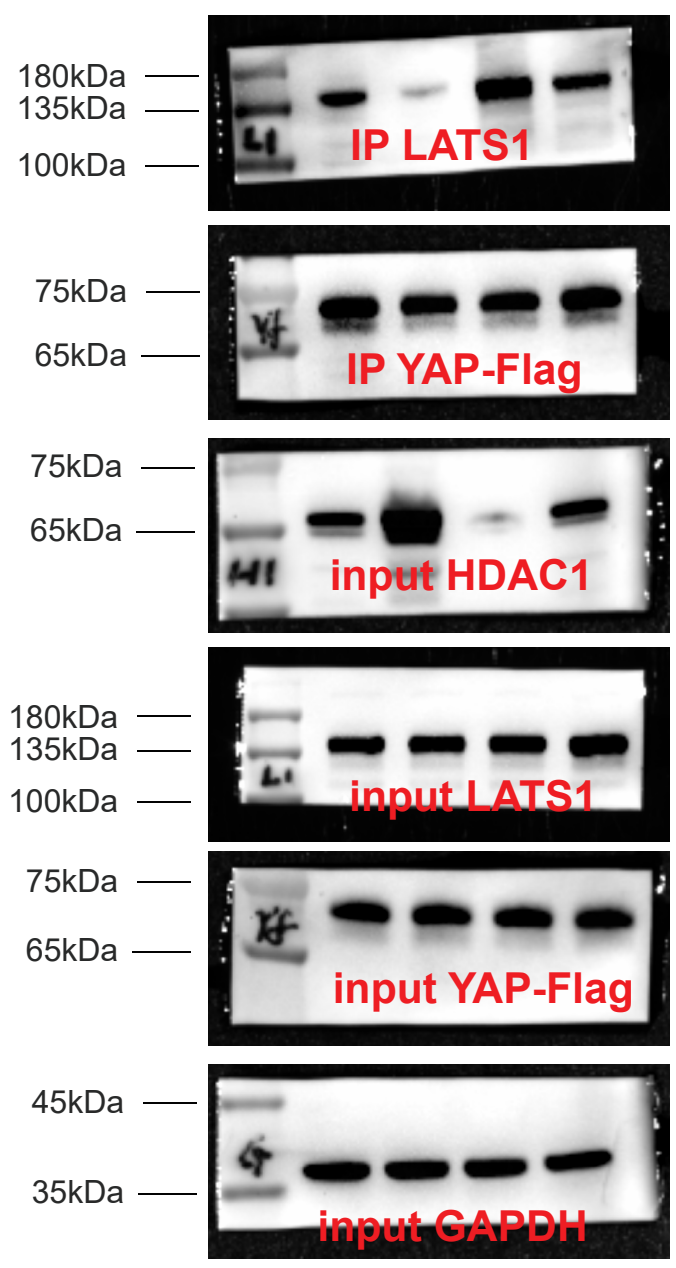

Figure S5M

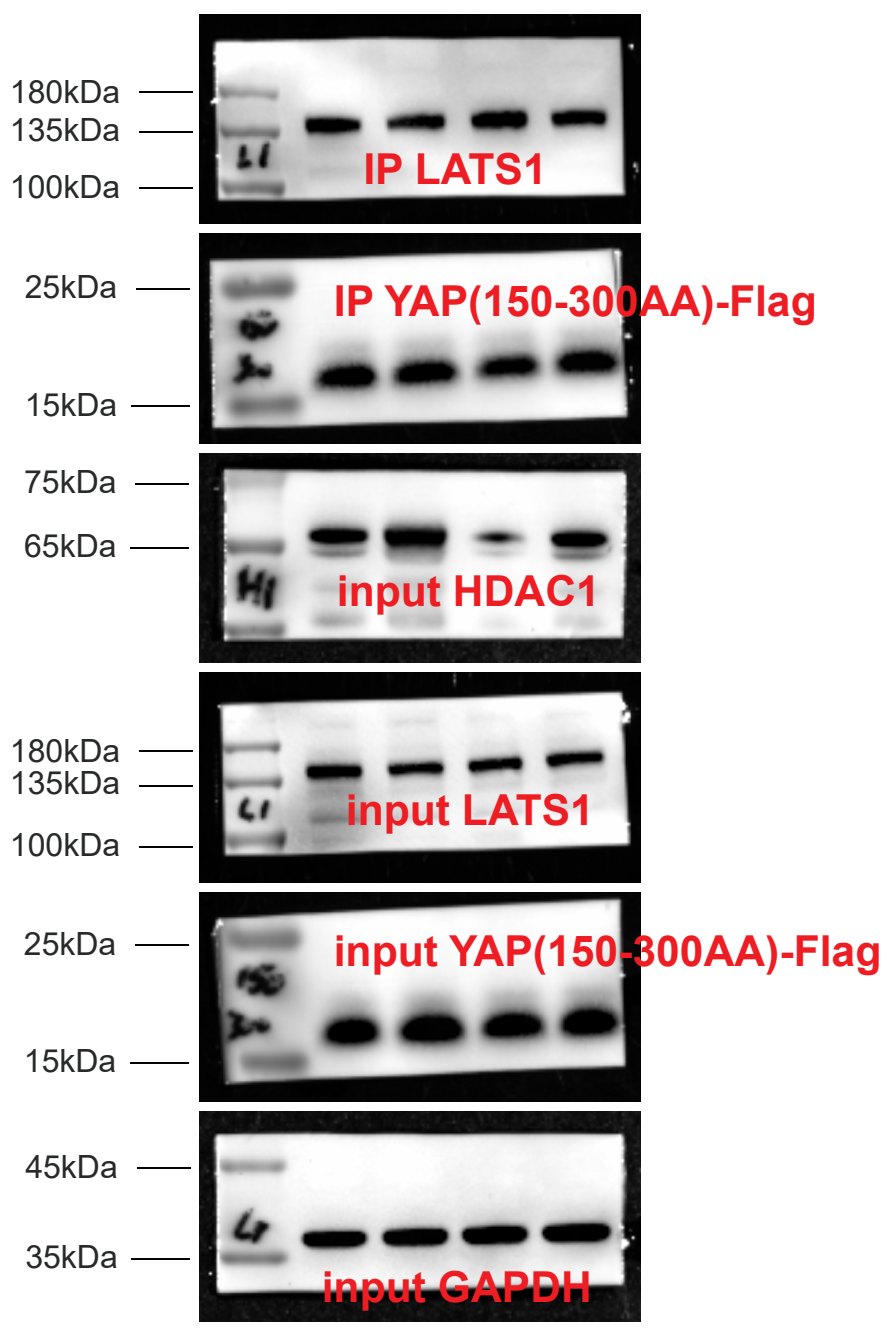

Figure S5N

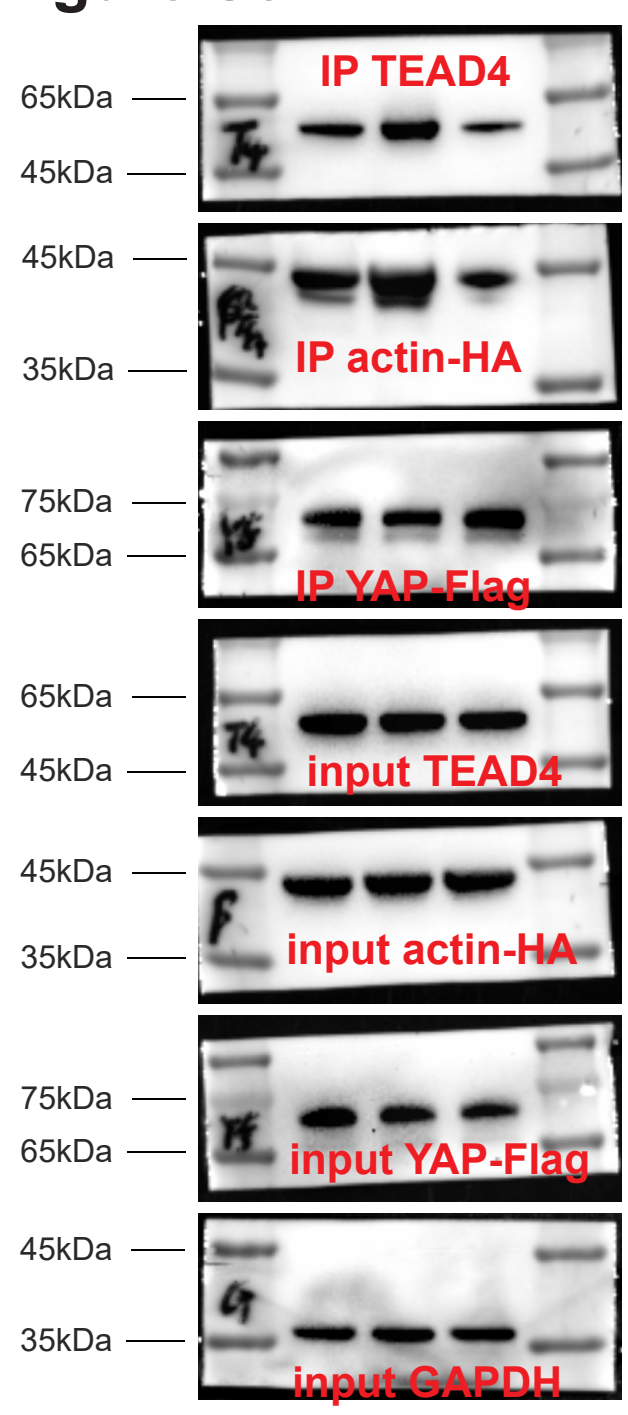

WB images in Figure S6

Figure S6A

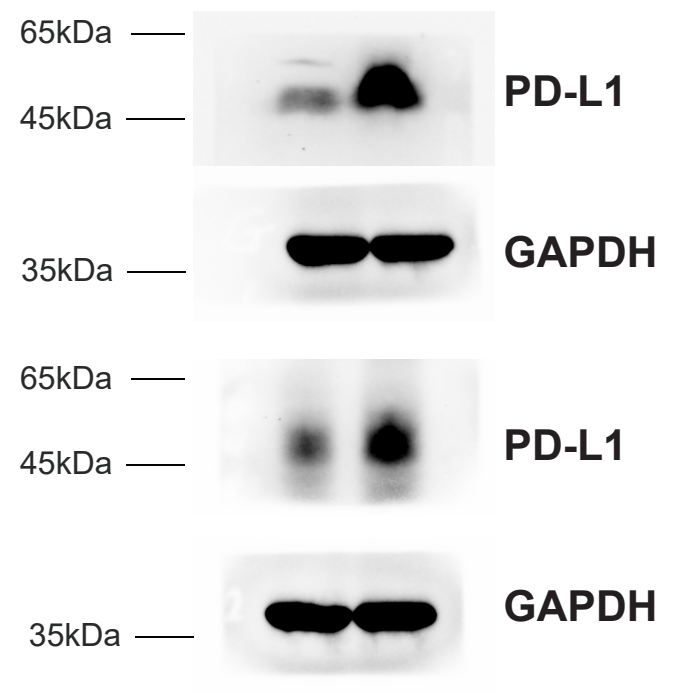

Figure S6B

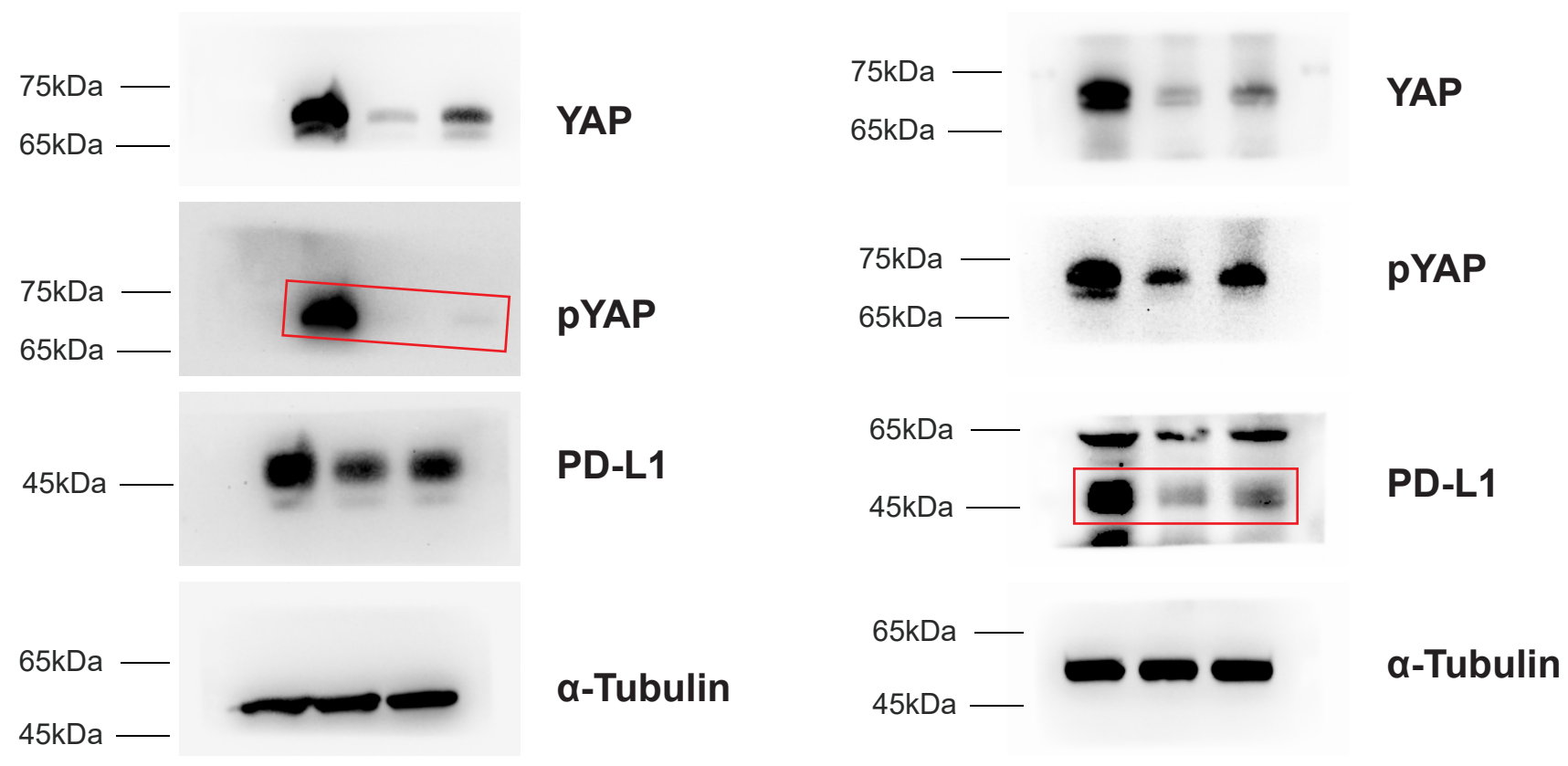

Figure S6C

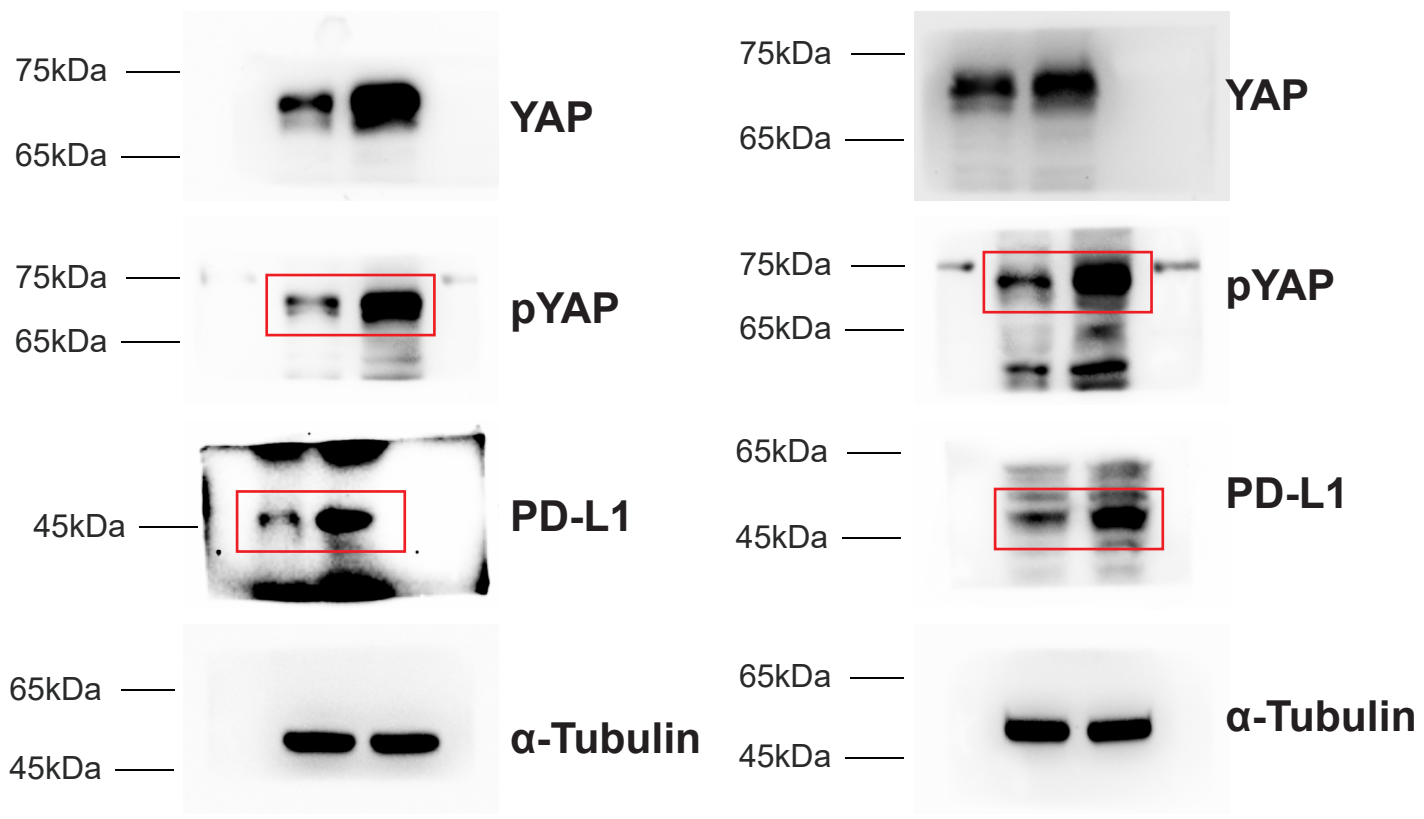

Figure S6D

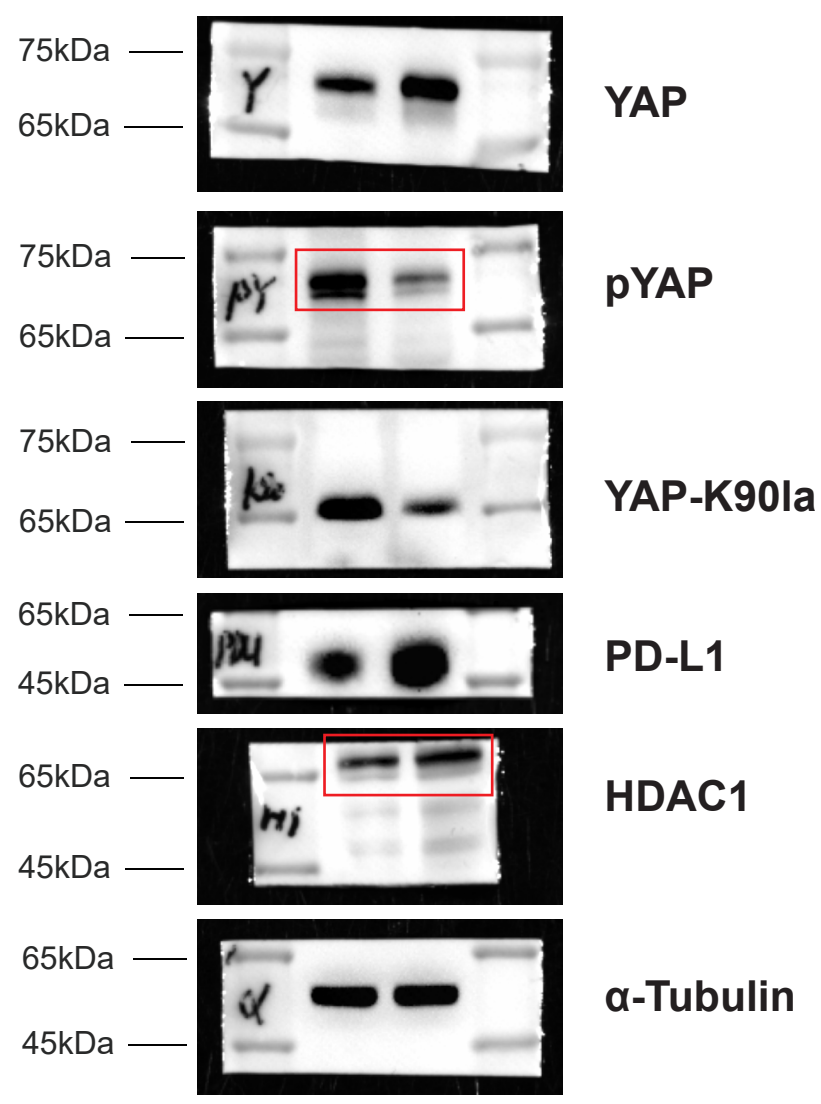

Figure S6E

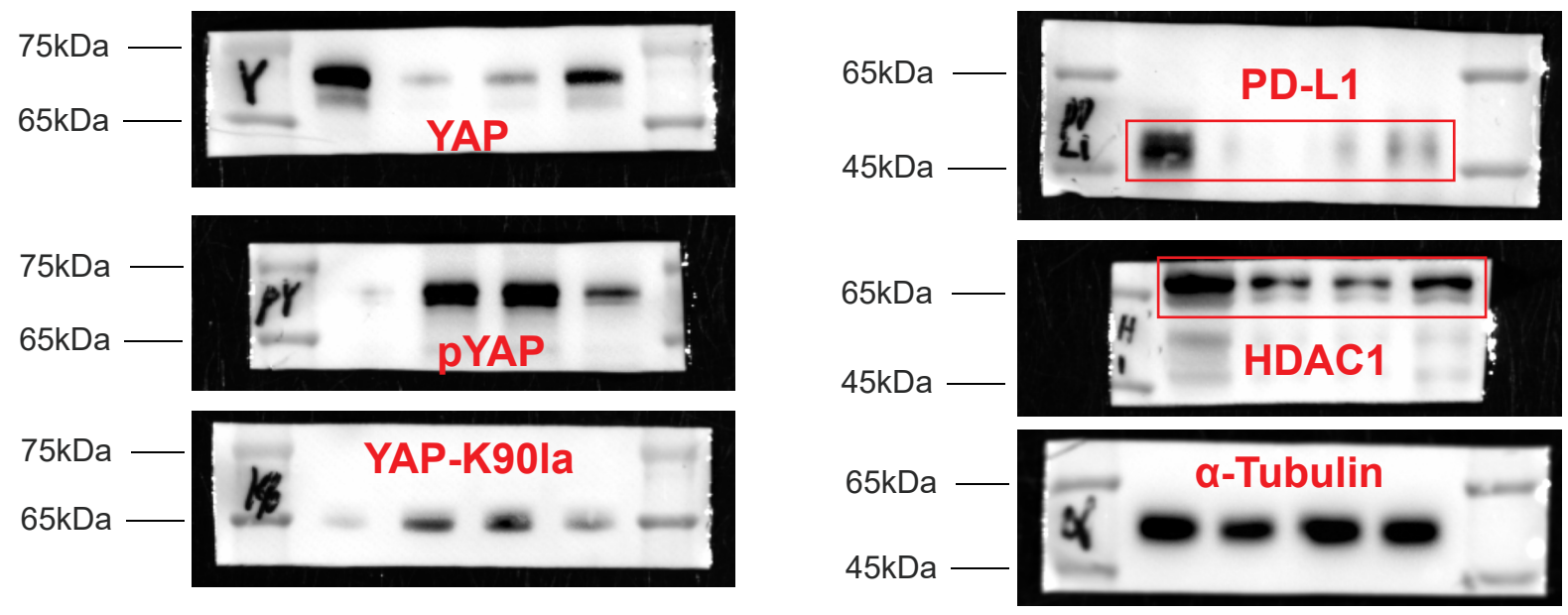

Figure S6G

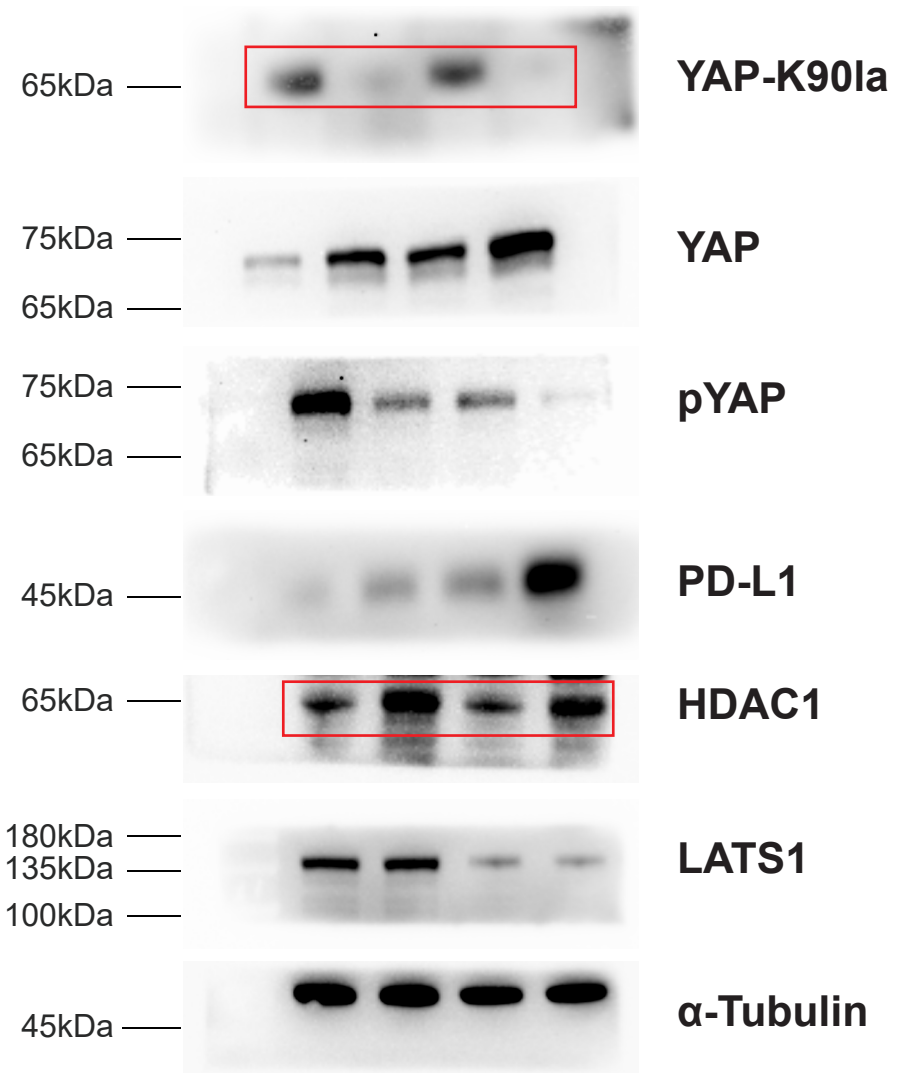

Figure S6H

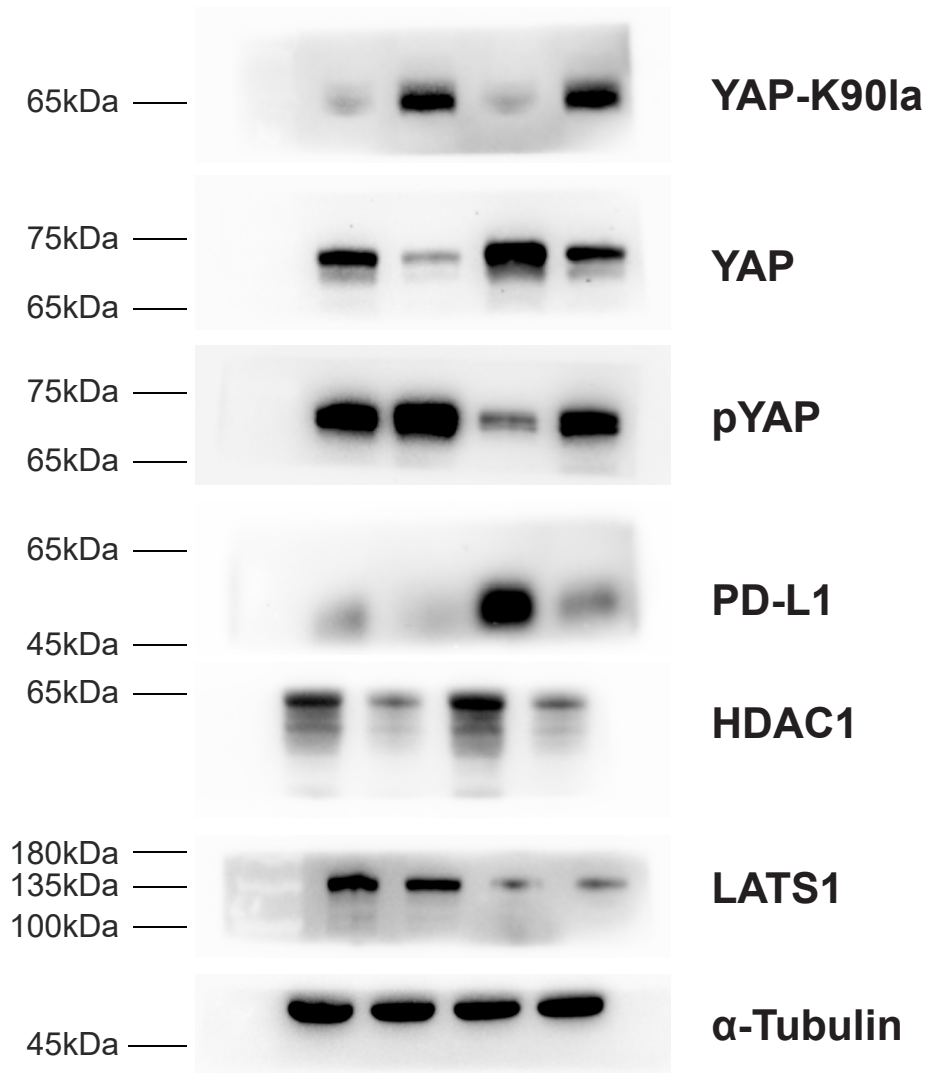

Supplement: Supplementary file 2 [file 41419_2026_9204_MOESM2_ESM.pdf]
